# Akamptisomerism Beyond Porphyrins: Bond Angle Reflection and Stereochemical Divergences in Corrole- and Porphyrin-Anchored B–O–B Bridges

**DOI:** 10.1021/acs.inorgchem.5c04528

**Published:** 2025-12-22

**Authors:** Karine N. de Andrade, Patrick L. L. Rocha, Daniella B. de Miranda, Natalia M. Raffaeli, Glaucio B. Ferreira, Rodolfo G. Fiorot

**Affiliations:** 1 Department of Organic Chemistry, Chemistry Institute, 28110 Universidade Federal Fluminense (UFF), Outeiro de São João Batista, Niterói, RJ 24020-141, Brazil; 2 Department of Inorganic Chemistry, Chemistry Institute, 28110 Universidade Federal Fluminense (UFF), Outeiro de São João Batista, Niterói, RJ 24020-141, Brazil

## Abstract

Main-group coordination-driven porphyrins (MGCPs) are an emerging class of complexes featuring central main-group elements. Bis-boron­(III) B_2_OF_2_-porphyrinoids, including porphyrins and corroles, exhibit a rich coordination chemistry with a (F)­B–O–B­(F) bridge bound to the N_4_ core. Porphyrins adopt a *transoid* bridge, while corroles favor a *cisoid* arrangement. These systems enable investigation of akamptisomerism — an underexplored conformational isomerism driven by restricted bond angle reflection (BAR) at the oxygen bridge. Density functional theory was used to investigate *cisoid*/*transoid* preference, the BAR mechanism, and stabilization of the *cisoid* isomer via noncovalent interactions. Geometric and natural bond orbital analyses revealed that *transoid* porphyrins, though more distorted, are stabilized by strong N → B interactions, whereas corroles favor *cisoid* geometry due to reduced distortion. Calculated *transoid*-BAR barriers were accessible for porphyrin (Δ*G* ^‡^ ≈ 25 kcal mol^–1^) but prohibitive for corrole (Δ*G* ^‡^ > 40 kcal mol^–1^), consistent across different theoretical levels. Activation strain and energy decomposition analyses showed that macrocycle-bridge interaction destabilization dominates barrier heights, with orbital interactions aiding BAR in porphyrins but hindering it in corroles. Substituent effects (Y in B_2_OY_2_ bridges) further stabilized *cisoid* geometries, though insufficient to allow *cisoid*-BAR. These findings offer insights into the coordination chemistry of B_2_OF_2_-porphyrinoids and a framework for rationalizing akamptisomerism in main-group macrocycles.

## Graphical abstract

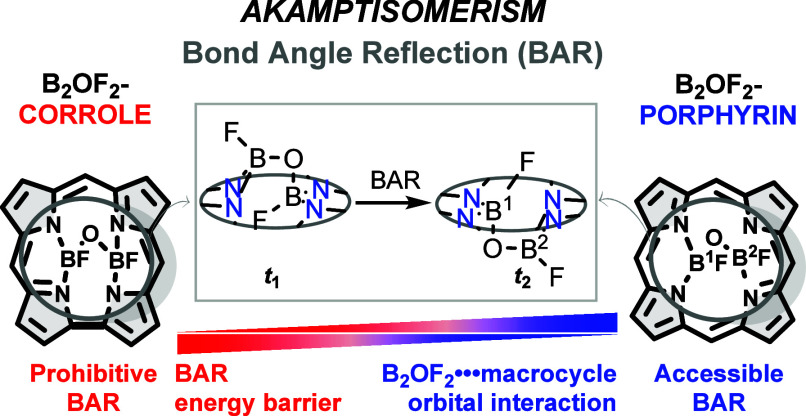

## 1 Introduction

Complexes derived from porphyrinoid macrocycles, such as porphyrin (**Por**) and its ring-contracted analogue corrole (**Cor**) (Figure 1a), play a central role in developing novel chromophores, therapeutic agents, and functional materials for technological applications.^1–6^ More recently, increasing attention has been given to the coordination chemistry of nonmetallic porphyrin complexes, particularly main-group coordination-driven porphyrins (MGCPs), in which the porphyrinoid core binds main-group elements, most commonly p-block atoms such as boron, silicon, and phosphorus.^7^ Among these, boron­(III) complexes stand out due to their remarkably rich and versatile coordination chemistry.^7,8^

**Figure 1:**
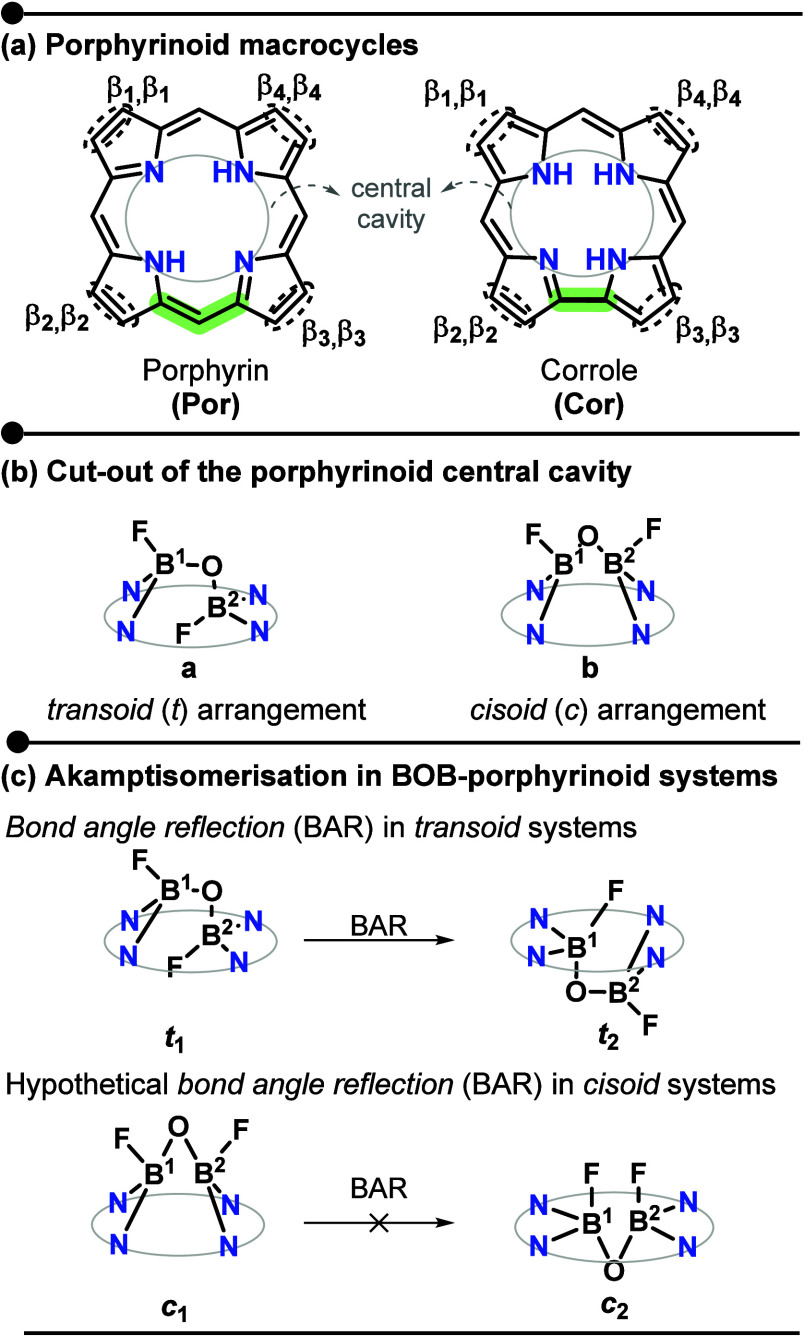
(a) Structures of porphyrin (**Por**) and its contracted analogue corrole (**Cor**), showing the β-substituents, the central N_4_ cavity, and highlighting in green the methinic unit responsible for the contraction in corrole. (b) Cutout of the central cavity of B_2_OF_2_-porphyrinoid complexes illustrating the two possible arrangements defined by the relative orientation of the fluorine atoms: *transoid* and *cisoid*. (c) Akamptisomerism via bond angle reflection (BAR) at the B_2_OF_2_ bridge in *transoid* B_2_OF_2_-porphyrin systems experimentally identified. Hypothetical *cisoid* pathways are shown for comparison.

Boron-based MGCPs can be obtained by reacting the porphyrinoid free base with BF_3_·OEt_2_ under basic conditions, producing a B–O–B bridge at the central cavity^9–11^ that may adopt either a *transoid* (*t*) or *cisoid* (*c*) arrangement (Figure 1b), depending on the macrocyle. In general, porphyrins preferentially form the *transoid* isomer,^12–15^ whereas B_2_OF_2_-corrole complexes favor a *cisoid* configuration.^16^ The incorporation of the B_2_OF_2_ bridge induces geometric distortions, relative to the free base. These distortions may occur in-plane, involving deviation of the N_4_ core from its ideal square geometry, or out-of-plane, producing macrocyclic deformations. As a result, *transoid* structures typically adopt a stepped (or wave-like) conformation, whereas *cisoid* forms tend to display doming.^12–15^

The contrasting stabilities of *cisoid* and *transoid* B_2_OF_2_ arrangements in corrole and porphyrin have been linked to the greater flexibility of the larger porphyrin cavity to accommodate a boron­(III) center.^15^ Yet, with the growing number of B_2_OF_2_-bridged complexes featuring diverse structural and electronic profiles, a deeper thermodynamic understanding has become increasingly relevant. Notably, this broader family has also revealed a newly recognized form of isomerism: akamptisomerism.^17–23^

Further enriching the coordination chemistry of these boron complex, Canfield and co-workers in 2018 discovered akamptisomerism—the last type of conformational isomerism, according to the authors.^23^ This process is characterized by the restricted bond angle reflection (BAR)^24^ of the central oxygen atom in the B_2_OF_2_ bridge anchored to a porphyrin macrocyle, identified in a *transoid* complex (Figure 1c). Despite the hypothetical possibility of a *cisoid* configuration, no *cisoid* isomers were experimentally identified in this case. For the *transoid* configurations, the computed thermal interconversion barrier exceeds 22 kcal mol^–1^, which make them a pair of isolable isomers.^23,25^ Furthermore, by anchoring this bridge in a low-symmetry porphyrin, the akamptisomers feature a diastereomeric relationship and, consequently, distinct physical and optoelectronic properties.^23^

To date, only our recent work has explored the versatility of akamptisomerism in porphyrinoid macrocycles, specifically investigating the influence of β- and *meso*-substituents on bond angle reflection processes and their impact on the absorption profiles of individual akamptisomers.^25^ In that study, we examined both the kinetic and thermodynamic aspects of BAR across 28 pairs of *transoid* akamptisomers, as well as their relative stabilities compared to the *cisoid* form. According to our calculations (B3LYP-D3/def2-QZVP//B3LYP-D3/6-31+G**), the diverse substitution patterns had a minor effect on the computed mean energy barrier with Δ*G* ^‡^ = 26.6 ± 2.1 kcal mol^–1^, with nearly isoenergetic akamptisomers. From a photophysical standpoint, one specific substitution pattern resulted in a distinct spectral difference between the individual isomers, showing a separation of approximately 321 meV in the absorption corresponding to the third excited state.^25^ This systematic evaluation provides insight into the overall profile of BAR and opens new horizons for applications in photoresponsive molecular systems. Excited-state evaluations of bond angle reflection (BAR) are currently in progress within our research group, with initial findings indicating promising prospects for phoswitch-type applications.

Herein, we aim to rationalize the influence of macrocycle size on the akamptisomerism phenomenon through density functional theory (DFT) calculations by directly comparing porphyrin and corrole frameworks. We seek to provide additional insights into the contrasting preferential stability of the Por­(B_2_OF_2_) complex for the *transoid* configuration and of [Cor­(B_2_OF_2_)]^−^ for the *cisoid* configuration, using ring deformation energy measurements, second-order perturbation theory analysis of the Fock matrix on a natural bonding orbital (NBO)^26^ basis. To elucidate the origin of the energy barrier heights associated with bond angle reflection processes for both **Por** and **Cor** complexes, we employed the activation strain model (ASM)^27^ and energy decomposition analysis (EDA).^28^ Finally, aiming to revert the stereochemical preference (*transoid* vs *cisoid*) and to explore new conformational isomers stabilized by noncovalent interactions between the bridge moiety atoms, a series of B_2_OY_2_ units were screened in corrole and porphyrin complexes. Different Y substituents were varied to identify Y•••Y interactions that could favor the *cisoid* form. We expect that the findings reported herein will provide essential insights and establish a novel framework for rationalizing trends and uncovering the origins of the unique coordination chemistry of bis-boron­(III) porphyrinoid complexes containing the B_2_OF_2_ bridge.

## 2 Computational Details

Density functional theory calculations were performed using the Gaussian09 program.^29^ Given the novelty of the investigated phenomenon, we adopted the same theoretical model used in Canfield and co-workers’ seminal work.^23^ B3LYP^30–32^ hybrid functional, corrected with the D3 empirical dispersion model,^33^ was employed in combination with the 6-31+G** basis set.^34,35^ The choice of functional was also motivated by its previous application in *in silico* studies of porphyrinoid geometry and conjugation, as well as other tetrapyrrolic aromatic compounds.^12,16,23,36,37^ Additionally, dispersion-corrected DFT methods have been reported to perform better in characterizing long-range interactions in free-base porphine (the nonsubstituted porphyrin), its metal derivatives, and various large organic molecules.^38,39^ To assess the results dependence on the level of theory and the robustness of our results, the def2-TZVP^40^ basis set was also tested in combination with the B3LYP-D3, CAM-B3LYP,^41^ and M06-2X^42^ functionals.

All geometries were fully optimized in the gas phase, and vibrational frequency calculations were performed to characterize stationary points and obtain thermodynamic data. Stable isomers were identified by the absence of imaginary frequencies, while transition states (first-order saddle points) were confirmed by the presence of a single imaginary frequency. Enthalpy and Gibbs free energy values were computed at 298 K and 1 atm using the default thermochemical equations in the Gaussian09 software. For the stable isomers, relative stability between the *cisoid* and *transoid* forms was further evaluated by natural bond orbital (NBO 6.0 version)^43^ analysis, performed at the same level of theory as the geometry optimizations, using the Gaussian09.

Two approaches have been reported for quantifying the in-plane distortion (Δ*d̅*_N•••N_) of B_2_OF_2_-anchored macrocycles: (i) comparing the perpendicular and parallel N•••N distances within the complexed structure itself,^13,14^ or (ii) comparing the N•••N distances in the N_4_ cavity between the free-base macrocycle and its B_2_OF_2_-complexed form.^12^ In this work, we adopted the latter. To this end, the free-base forms of both macrocycles were fully optimized: the neutral monobenzo-fused porphyrin (**PorBz**) and the anionic monobenzo-fused corrole (**CorBz**). The anionic state of corrole was used to ensure a consistent overall −1 charge between the free-base and complexed structures. Thus, the N•••N distances between the four nitrogen atoms in the central cavity of each optimized macrocycle structure (both complexed and free-base) were compared, and the in-plane distortion was calculated as follows:

$$△{\mathrm{d̅}}_{N\cdot \cdot \cdot N}={\mathrm{d̅}}_{N\cdot \cdot \cdot N}[B_{2}{\mathrm{OF}}_{2}‐\mathrm{complex}]-{\mathrm{d̅}}_{N\cdot \cdot \cdot N}[\mathrm{free}\mathrm{base}]$$ 1

Corrole prosesses two distinct N•••N distances; the values reported here correspond to their arithmetic mean. A schematic illustrating the projection procedure and the definition of Δ *d̅*_N•••N_ is provided in Figure S1 of the Supporting Information.

The electronic factors controlling the bond angle reflection energy barriers was investigated using the activation strain model (ASM).^44^ This fragmentation-based approach enables rationalization of the energy barrier relative to the reactant, which, in the case of akamptisomerism, corresponds to the stable isomer. Along the intrinsic reaction coordinate (IRC), the total electronic energy Δ*E*(ζ) is decomposed into strain energy Δ*E*_strain_(ζ) and interaction energy Δ*E*_int_(ζ), according to eq 2:

$$\Delta E\left(\zeta \right)=\Delta E_{\mathrm{int}}\left(\zeta \right)+\Delta E_{\mathrm{strain}}\left(\zeta \right)$$ 2

The strain energy reflects the energetic cost of deforming the reactants from their equilibrium geometries and offers insight into structural reorganization along the reaction pathway. The interaction energy accounts for the interaction between the distorted fragments and can be further decomposed by energy decomposition analysis (EDA) into three fundamental components:

$$\Delta E_{\mathrm{int}}\left(\zeta \right)=\Delta E_{\mathrm{elstat}}\left(\zeta \right)+\Delta E_{\mathrm{Pauli}}\left(\zeta \right)+\Delta E_{\mathrm{oi}}\left(\zeta \right)$$ 3

where Δ*E*_elstat_ represents electrostatic interactions, Δ*E*_Pauli_ is the Pauli (exchange) repulsion, and Δ*E*_oi_ corresponds to orbital interactions.^28^ The combined use of ASM and EDA is well-established in the literature and provides valuable insights into reaction mechanisms, catalytic activity, and conformational preferences.^44–49^ For bond angle reflection, B_2_OF_2_-porphyrinoid systems were partitioned into two fundamental fragments: the tetrapyrrolic macrocycle (neutral **Por** and anionic **Cor**) and the anchored bridge (neutral B_2_OF_2_). This fragmentation scheme was selected to elucidate the electronic factors governing the direct bond angle reflection of the B_2_OF_2_ bridge when bound to the tetrapyrrolic framework. The ASM analysis along the BÔB coordinate (i.e., the bridge angle) was carried out using the PyFrag program^50^ in conjunction with the Gaussian09 package.

For the EDA calculations, the same fragments were considered to understand the interaction energy behavior between the macrocycle and the B_2_OF_2_ bridge. The calculations were performed using the GAMESS software (version 30 SEP 2020 - R2),^51,52^ based on the optimized structures of the systems and employing the same level of theory.

## 3 Results and Discussion

Akamptisomerization, that is, the bond angle reflection (BAR) of the B_2_OF_2_ bridge, remains underexplored in the literature. Herein, we investigate the macrocycle size influence on this phenomenon by comparing porphyrin and corrole, two systems in which the anchoring of the B_2_OF_2_ bridge is well documented.^8,10,12,18^ To the best of our knowledge, only our recent work has systematically evaluated BAR in porphyrin systems.^25^ Although we provided a comprehensive analysis of substituent effects and photophysical properties, the macrocycle size has not been addressed. To gain a deeper understanding of this phenomenon, we first present a general exploration of comparative BAR in porphyrin and corrole including the calculations of energy barriers and the thermodynamic stability of individual isomers. We then analyze the relevant factors to the relative stability of *transoid* and *cisoid* configurations to each macrocycle. Next, to assess the akamptisomerization feasibility, we investigate the origin of the BAR energy barrier in each macrocycle by using the activation strain model (ASM). Finally, we explore the preferential formation of the *cisoid* isomer in response to variations in the bridge composition.

The lower symmetry of corrole compared with porphyrin gives rise to two possible anchoring modes for the B_2_OF_2_ bridge. In the parallel mode, the B–O–B axis is aligned with the direct pyrrole–pyrrole connection of the macrocycle (the bold bond in the corrole structure; Figure 2a). In the perpendicular mode, the bridge is oriented orthogonally to this connection. Although experimental studies^16^ have identified the parallel mode in *cisoid* B_2_OF_2_-corrole complexes, both anchoring modes were evaluated computationally (see Section 2 of the Supporting Information). Our calculations show that the parallel arrangement is significantly more stable, by Δ*G* ≈ 18–20 kcal mol^–1^, across all tested functionals (B3LYP-D3, M06-2X, and CAM-B3LYP). This stability correlates with a smaller tetragonal in-plane distortion of the macrocyclic cavity, quantified by the metric Δ*d*_N•••N_, which measures the change in the N•••N separation aligned with the B•••B axis upon bridge anchoring.^12,16^ As detailed in Figure S2b and Table S1, the parallel mode consistently produces a reduced Δ*d*_N•••N_ relative to that of the perpendicular mode, indicating that it better preserves the natural geometry of the free-base macrocycle. Given this clear energetic and geometric preference, all pathway analyses presented in the main text were conducted using the parallel anchoring mode.

**Figure 2:**
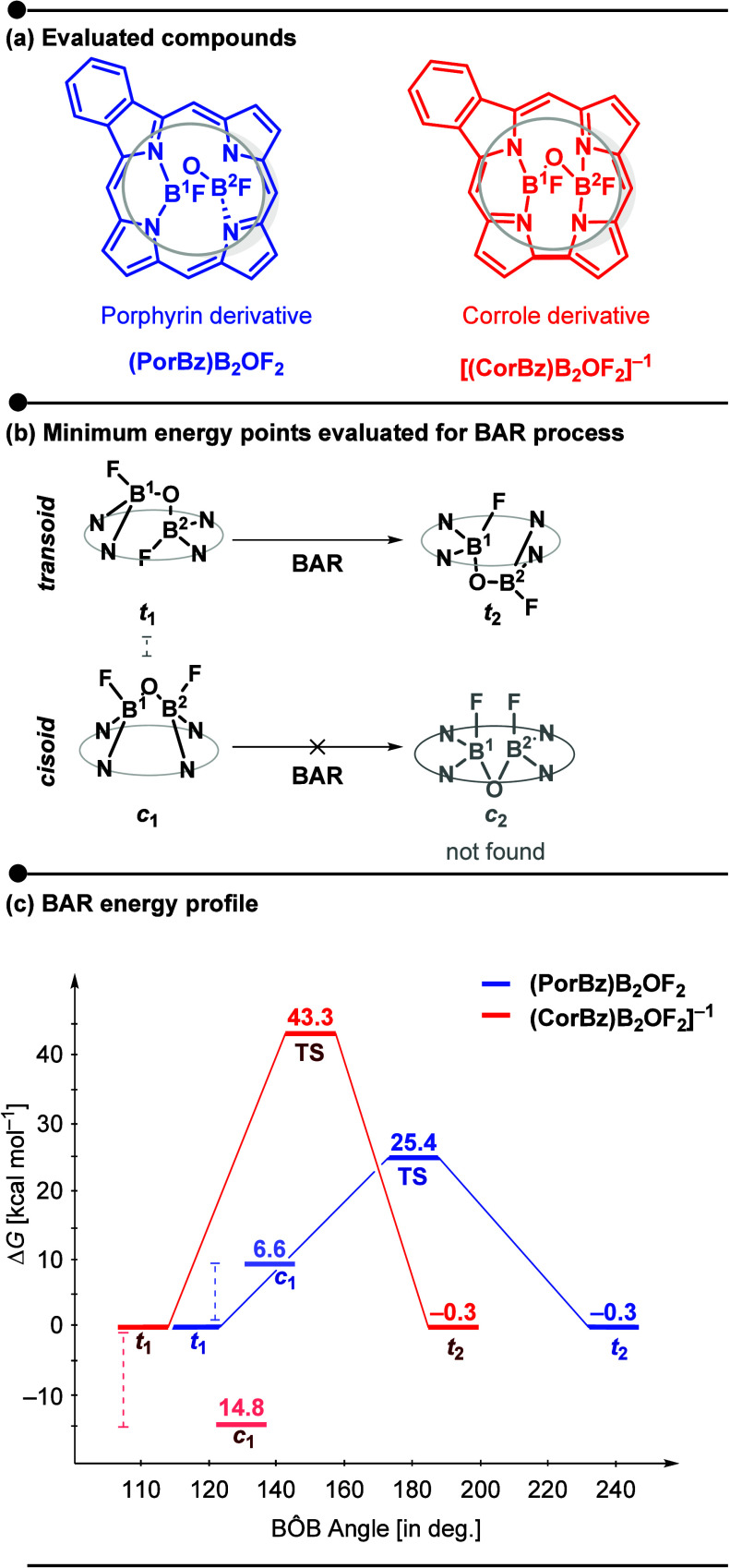
(a) Evaluated porphyrinoid complexes: (PorBz)­B_2_OF_2_ (porphyrin derivative, blue) and (CorBz)­B_2_OF_2_]^−1^ (corrole derivative, red). (b) Cut out representations of the B_2_OF_2_-bridged cavities explored in the bond angle reflection (BAR) analysis, showing the processes connecting the *transoid* (***t*_1_** → ***t*_2_**) and *cisoid* (***c*_1_** → ***c*_2_**) arrangements. The ***c*_2_** isomer was not a minimum and optimizations converged to ***c*_1_**. (c) Gibbs free energy change (kcal mol^–1^) for BAR in porphyrin (blue) and corrole (red), showing activation barriers (Δ*G* ^‡^, TS—transition states) and reaction free energy changes (Δ*G*, ***t*_2_**), all referenced to the *transoid**t***_**1**_ minimum. Relative energies of ***c*_1_** are also given as Δ*G* = *G**c***_**1**_ *– G**t***_**1**_). Calculations were performed at the B3LYP-D3/6-31+G** level.

The B_2_OF_2_-BAR can theoretically occur in both corrole and porphyrin macrocycles, yielding the two pairs of *cisoid* (***c***_**1**_ and ***c***_**2**_) and *transoid* (***t***_**1**_ and ***t*_2_**) configurations. To investigate this process, we employed monobenzo-fused pyrrole macrocycles as models: (PorBz)­B_2_OF_2_ for porphyrin and [CorBz­(B_2_OF_2_)]^−^ for corrole. This structural modification reduces the symmetry of the macrocycles, enabling a clear differentiation between the corresponding akamptisomers. Figure 2b shows cut out representations of the B_2_OF_2_-bridged porphyrinoid cavities, highlighting the minimum-energy structures explored for the *transoid* (***t***_**1**_ and ***t*_2_**) and *cisoid* (***c***_**1**_ and ***c*_2_**) arrangements. Figure 2c presents the computed energy profiles for the BAR processes of the monobenzo-fused porphyrin (blue) and corrole (red).

For the corrole (red), the *cisoid* (***c***_**1**_) configuration was computed to be 14.8 kcal mol^–1^ more stable than the *transoid* (***t***_**1**_ and ***t*_2_**) configurations, aligning with the experimentally characterized products—Figure 2c, ***c***_**1**_ in light red.^16^ In contrast, but also agreeing with experimental observation,^12,15^ the *transoid* porphyrin (blue) is 6.6 kcal mol^–1^ more stable than the *cisoid* (***c***_**1**_), as showed in Figure 2c, ***c***_**1**_ in light blue. Despite several attempts, the hypothetical ***c***_**2**_ isomer could not be optimized as a minimum point on the potential energy surface (PES). Regardless of the macrocycle, all optimizations of ***c***_**2**_ converged to the ***c***_**1**_ configuration, where both boron atoms are positioned above the pseudoplane of the macrocycle, creating a domed shape. This is in agreement with the previous observations by Canfield and co-workers^23^ for the quinoxalineporphyrin system, as well as with our group’s work on several *meso*- and β-substituted porphyrin derivatives.^25^

The *transoid* isomers (***t***_**1**_ and ***t*_2_**) were obtained for both macrocycles, along with the transition states connecting these two isomers—Figure 2c, TS in blue and red. For the porphyrin, the calculated bond angle reflection barrier is Δ*G* ^‡^ = 25.4 kcal mol^–1^, consistent with the experimental observation of the akamptisomerism phenomena.^23^ However, for corrole, the calculated value revealed a prohibitive barrier with Δ*G* ^‡^ = 43.4 kcal mol^–1^, causing the interconversion to be kinetically limited. These energetic trends—both in isomer stability and in the BAR barriers—were consistent across different levels of theory, including B3LYP-D3, M06-2X, and CAM-B3LYP combined with the def2-TZVP basis set (Table S2, Supporting Information). In addition, solvent effects were evaluated using implicit solvation in dichloromethane (CH_2_Cl_2_), matching the experimental conditions, and the same qualitative trends were confirmed (Table S3).

In the next section, we focus on the relative stability of the *transoid* and *cisoid* configurations in both macrocycles, aiming to identify structural and energetic factors that account for their stability differences.

### *Transoid* vs *Cisoid* Relative Stability

The *transoid* isomer is thermodynamically favored in porphyrins, whereas the *cisoid* is preferred in corroles—a trend previously reported in the literature by computational simulations and in good agreement with X-ray crystallographic data.^8,15,16^ Porphyrins possess a larger cavity (calculated free-base area: 8.6 Å^2^) compared to corrole (free base: 7.3 Å^2^), which may facilitate the accommodation of a boron atom within the macrocycle pseudoplane in *transoid* structures. The literature suggests that the preference for the *transoid* configuration is associated with a larger N•••N distance in this geometry: the greater the cavity size, the more stable the *transoid* structure.^15^ This trend may be related to the coordination environment of larger ligands, which can sustain greater tetragonal in-plane distortion and thus accommodate a boron atom in the pseudoplane of the macrocycle.

Figure 3 presents optimized geometric parameters of the bridge anchored to the N_4_ cavity of monobenzo-fused porphyrin and corrole macrocycles in both *transoid* and *cisoid* configurations, along with the mean tetragonal in-plane distortion of the cavity, (Δ*d*_N•••N_), the deformation electronic energy (Δ*E*_def_), and the Gibbs free energy of the *cisoid* configuration relative to the *transoid* arrangement (Δ*G*). This distortion is calculated relative to the free-base macrocycle and reflects how the N_4_ cavity is elongated to accommodate the B_2_OF_2_ bridge. The calculation method for Δ*d*_N•••N_ is detailed in Scheme 1 of the Supporting Information file.

**Figure 3:**
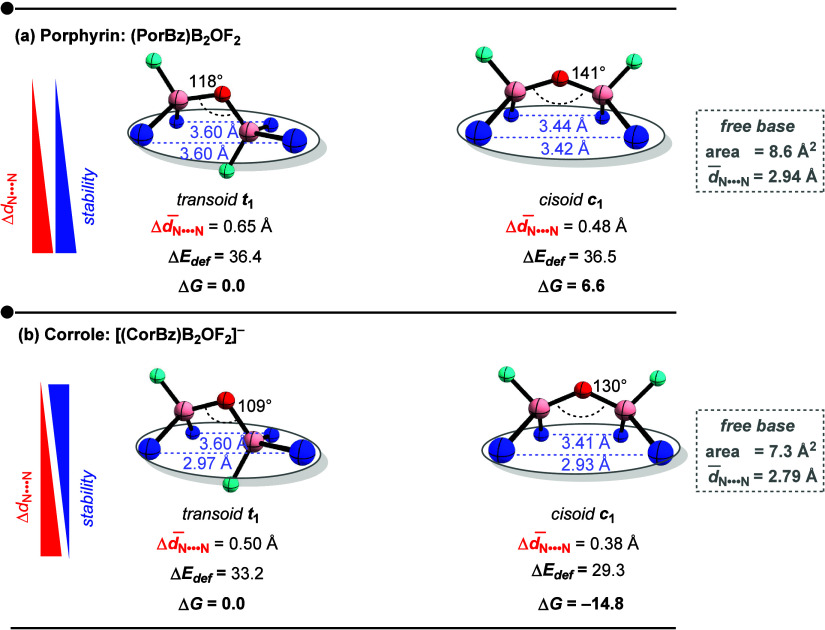
Geometric parameters of the central cavity for *transoid**t***_**1**_ and *cisoid**c***_**1**_ configurations of (a) porphyrin and (b) corrole B_2_OF_2_ complexes, including N•••N distances and the BÔB angle. Tetragonal in-plane distortion was computed relative to the optimized free-base macrocycles by using eq 1. Deformation electronic energy (in kcal mol^–1^) is reported as Δ*E*_def_ = *E*_deformed ring_ – *E*_free base_. All geometric and energetic data correspond to optimized structures at the B3LYP-D3/6-31+G** level. Color atoms: nitrogen (blue), boron (pink), fluorine (cyan), and oxygen (red).

Unexpectedly, for porphyrin (Figure 3a), the more stable configuration (*transoid*) exhibits a larger tetragonal in-plane distortion (Δ *d̅*_N•••N_ = 0.65 Å) than the *cisoid* configuration (0.48 Å). Conversely, corrole (Figure 3b) follows the expected trend: the *cisoid* arrangement shows lower distortion (0.38 Å) than its *transoid* geometry (0.50 Å), consistent with the general preference for reduced distortion and enhanced stability.

In addition to tetragonal in-plane distortion, we calculated the ring deformation energies Δ*E*_def_, defined as the electronic energy difference between the isolated macrocyclic ring frozen in the geometry it adopts within the complex and the fully optimized free-base macrocycle:

$$\Delta E_{\mathrm{def}}=E_{\mathrm{deformed}\;\mathrm{ring}}-E_{\mathrm{free}\;\mathrm{base}}$$ 4

It is important to note that Δ*E*_def_ quantifies the energetic cost required to deform the macrocycle to accommodate the B_2_OF_2_ bridge but does not by itself describe the type of out-of-plane distortion adopted. Structurally, the *transoid* B_2_OF_2_-porphyrin complexes exhibit a wave-like (stepped) deformation, whereas the *cisoid* B_2_OF_2_-corrole complexes are domed. The deformation energies follow the expected trend: in corrole, the *transoid* isomer has a larger deformation energy (33.2 kcal mol^–1^) than that of the *cisoid* isomer (29.3 kcal mol^–1^), supporting the greater stability of the latter.

In the case of the porphyrin complexes, however, the ring deformation energies are essentially identical for the *cisoid* and *transoid* configurations, despite the distinct in-plane and out-of-plane distortions required for each geometry. The relative stability of the *transoid* structure therefore most likely arises from the geometry of the B_2_OF_2_ bridge itself—specifically, a preferential bond angle at the oxygen center.

To gain a deeper understanding of the B_2_OF_2_ bridge, we performed natural bond orbital (NBO) analysis. This allowed us to evaluate the donor–acceptor interaction energies (*E*_NBO_) between atom (or groups) pairs. Also known as second-order perturbation stabilization energy, *E*(2), this interaction corresponds to the stabilization resulting from the superposition of the electronic densities of the charge donor and acceptor orbitals.^26^ Higher *E*(2) (or *E*_NBO_) values indicate stronger covalent interactions and more pronounced stabilization. This analysis has been widely employed to investigate structural and reactivity trends across a variety of compounds, highlighting its utility in probing preferred conformations of oxygen-containing molecules and elucidating the anomeric effect in such systems.^53,54^ In addition, NBO analysis provides spatial representations of the natural orbitals, offering insights into the nature of the bonding interactions. Section 3 of the Supporting Information summarizes the NBO results and their correlations with interatomic distances for the *transoid* and *cisoid* forms in both macrocyclic frameworks.

For both macrocycles, the average NBO energy (*E̅*_NBO_) of the two O–B bonds showed only a minor contribution compared to the N–B interactions, indicating that O–B bonding is not the decisive factor to the configuration preference (Tables S4 and S5 as well as Figures S4 and S5, Supporting Information). The N–B interactions were consistently stronger in the *transoid* than in the *cisoid* configuration for both macrocycles, with stabilization energies 43.2 kcal mol^–1^ higher in porphyrin and 38.0 kcal mol^–1^ higher in corrole. When normalized by the number of atoms, this corresponds to average Δ*E*_NBO_ differences of 0.9 kcal mol^–1^ per atom for porphyrin and 0.8 kcal mol^–1^ per atom for corrole.

These NBO results suggest that although *transoid*-porphyrin presents higher in-plane distortion, its larger cavity and stronger N–B interactions favor the *transoid* configuration, which allows one boron atom to remain in-plane and maximizes N → B overlap. In corrole (the contracted porphyrin), *cisoid* stability appears to arise mainly from geometric factors related to tetragonal in-plane distortion, even though the *transoid* form also benefits from stabilizing N–B interactions. While the *transoid*-BAR process exhibits a prohibitive energy barrier in corrole, it remains energetically accessible in porphyrin—the macrocycle in which this type of isomerism was first discovered.^23^ Following, we investigated the origin of these barriers in both macrocyclic systems using activation strain analysis, decomposing the electronic energy into strain and interaction contributions.

### Bond Angle Reflection in *Transoid* Isomers

Interconversion between akamptisomers occurs through direct bond angle reflection (BAR) at the oxygen atom of the B_2_OF_2_ bridge. For the *cisoid* geometry, this pathway is not feasible, as the ***c***_**2**_ isomer does not correspond to a minimum-energy point on the PES. We therefore focused on the viable process involving the *transoid* isomers ***t*_1_** and ***t*_2_**. Despite the structural similarities, our results suggest that akamptisomerization is kinetically limited in corrole (Δ*G* ^‡^ = 43.4 kcal mol^–1^) but accessible in porphyrin, with a considerably lower barrier (Δ*G* ^‡^ = 25.4 kcal mol^–1^). In this section, we rationalize this energy profile.

The BAR is expected to proceed through a linear transition state, with the BÔB bridge angle approaching 180°, aligning the atoms diagonally across the porphyrin N_4_ cavity. However, in the corrole transition state, the smaller cavity size prevents this linearization (Figure 4a). In this case, the BÔB angle at the TS is 150°, suggesting that the BAR may involve two simultaneous and coupled B–O bond rotations.

**Figure 4:**
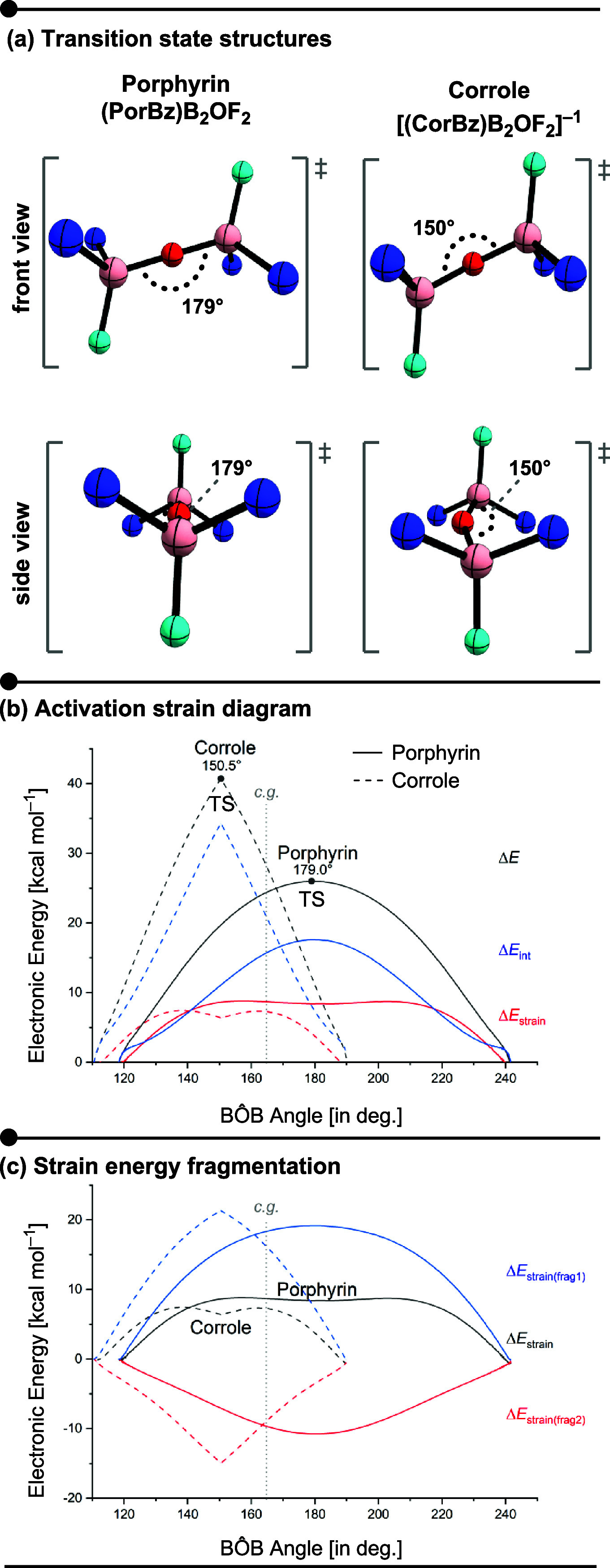
(a) Optimized transition-state geometries for the B_2_OF_2_ bond angle reflection in porphyrin and corrole, showing front and side views of the B–O–B angle (B3LYP-D3/6-31+G**). (b) Activation strain diagram relative to the bond angle reflection for the B_2_OF_2_ bridge (fragment 1) and the tetrapyrrolic macrocycle (fragment 2). Black curves: total electronic energy (Δ*E*); red: distortion energy (Δ*E*_strain_); blue: interaction energy (Δ*E*_int_). (c) Decomposition of the distortion energy (Δ*E*_strain_) for each fragment: black for the total distortion, blue for fragment 1, and red for fragment 2. Solid lines correspond to porphyrin; dashed lines correspond to corrole. The consistent geometry (c.g.) was defined at BÔB = 164.7° (gray vertical line). Energies were obtained at the B3LYP-D3/6-31+G** level along the IRC. Nitrogen (blue), boron (pink), fluorine (cyan), and oxygen (red).

The isolability of the individual akamptisomers depends on the height of the BAR barrier, opening opportunities for applications such as molecular switches.^25^ Understanding the factors that influence this phenomenon is crucial, particularly for expanding its application to different macrocycles—not only to contracted porphyrins such as corrole but also expanded analogues. To this end, the interaction–distortion model was employed to elucidate the structural and electronic factors that determine the activation barrier associated with the *transoid* isomer ***t***_**1**_. In this model, the electronic energy (Δ*E*) along the intrinsic reaction coordinate (IRC) is decomposed into interaction (Δ*E*_int_) and distortion energy (Δ*E*_strain_), where the B_2_OF_2_ bridge is considered fragment 1 and the tetrapyrrole macrocycle is considered fragment 2. The resulting activation strain diagram (ASD) for porphyrin (solid lines) and corrole (dashed lines) is shown in Figure 4b.

It is important to highlight that, while porphyrin exhibits a smooth energy profile for the bond angle reflection process, corrole shows a cusp at the transition-state point, which could indicate an irregular and nondifferentiable profile. However, this is possibly due to choosing the BÔB angle as the reaction coordinate. The entire reaction coordinate, together with its decomposition into interaction and strain energy contributions obtained from the intrinsic reaction coordinate (IRC) calculation—which naturally includes the concerted structural changes occurring during BAR—is provided in the Supporting Information (Figure S6). A smoothed profile for the BAR process is also shown.

According to the ASD, both interaction and strain contributions exhibit positive values along the entire BAR process, thus acting as destabilizing factors. The strain curves (in red) display a similar profile for both macrocycles, with a total contribution of less than 10 kcal mol^–1^. Figure 4c shows the strain decomposition in terms of the contribution of each fragment—i.e., Δ*E*_strain_ = Δ*E*_strain_(B_2_OF_2_) + Δ*E*_strain_(macrocycle). Interestingly, while the deformation of the bridge is always destabilizing (as it goes from the relaxed geometry approaching to a linear-like transition state at the oxygen atom), the macrocycle distortion along the BAR is stabilizing for both macrocycles, although more pronounced to corrole. Despite its intriguing behavior, the strain contribution is relatively small and follows a similar trend for both systems. Thus, the BAR energy barrier appears to be primarily governed by the interaction between the fragments—specifically, the bridge•••macrocycle interaction.

For a reliable comparison of akamptisomerism in each macrocycle, we used the average bond angle observed in the transition states as a consistent geometry (c.g.) for analysis—represented by the vertical gray line at BÔB = 164.7°—to qualitatively assess the factors influencing the BAR process. At this geometry, it becomes evident that the more destabilizing interaction energy is a key contributor to the higher activation energy observed for corrole. Notably, this destabilization reaches a magnitude comparable to the total electronic energy required for porphyrin akamptisomerization.

The electronic interaction energy (Δ*E*_int_) was further analyzed through energy decomposition analysis (EDA). To establish a correlation with the overall akamptisomerism energy profile, two key geometries were selected: (i) the transition state of each macrocycle, providing direct insight into the energy barrier of the isomerization process, and (ii) the consistent geometry, which allows a direct comparison between macrocycles under the same bridge angle.

Δ*E*_int_ corresponds to the sum of the electrostatic (Δ*E*_elstat_), orbital interaction (Δ*E*_oi_), and Pauli repulsion (Δ*E*_Pauli_) energies. In this context, the electrostatic terms refer to a quasi-classical interaction between the charge distributions of the isolated fragments: although the electron densities are calculated quantum mechanically, they are kept frozen during this step, resulting in an interaction that behaves in a classical electrostatic manner.^26,55^ The Pauli repulsion term arises from the antisymmetrization and renormalization of the wave function to comply with the Pauli exclusion principle and contributes in a destabilizing manner to the total interaction energy. Finally, the orbital interaction term represents the covalent contribution to bonding and encompasses donor–acceptor charge transfer, polarization, and electron-pair bonding.^55^ This orbital contribution (Δ*E*_oi_) can be further decomposed into exchange (Δ*E*_ex_), polarization (Δ*E*_pol_), and dispersion (Δ*E*_disp_) energies, according to the following expression:

$$\Delta E_{\mathrm{oi}}=\Delta E_{\mathrm{ex}}+\Delta E_{\mathrm{pol}}+\Delta E_{\mathrm{disp}}$$ 5

Table S6 shows the absolute energy of ***t***_**1**_ and the relative energies of **TS** and c.g., referenced to ***t***_**1**_, for both macrocycles. To allow for a consistent comparison between porphyrin and corrole, the energies were also normalized by the total number of atoms in each system, and the results are summarized in Tables S7 and S8, following the same approach adopted in the NBO analysis. In the discussion below, we focus on the normalized relative energy values at the consistent geometry (c.g., BÔB = 164.7°), providing a unified reference for comparing both macrocycles. The EDA results for the fragmentation of the interaction energy (Δ*E*_int_) and orbital interaction (Δ*E*_oi_) are shown in Figure 5, with the Δ*E*_int_ components (calculated using eq 3) represented in gray, and the Δ*E*_oi_ contributions (from eq 5) highlighted in purple.

**Figure 5:**
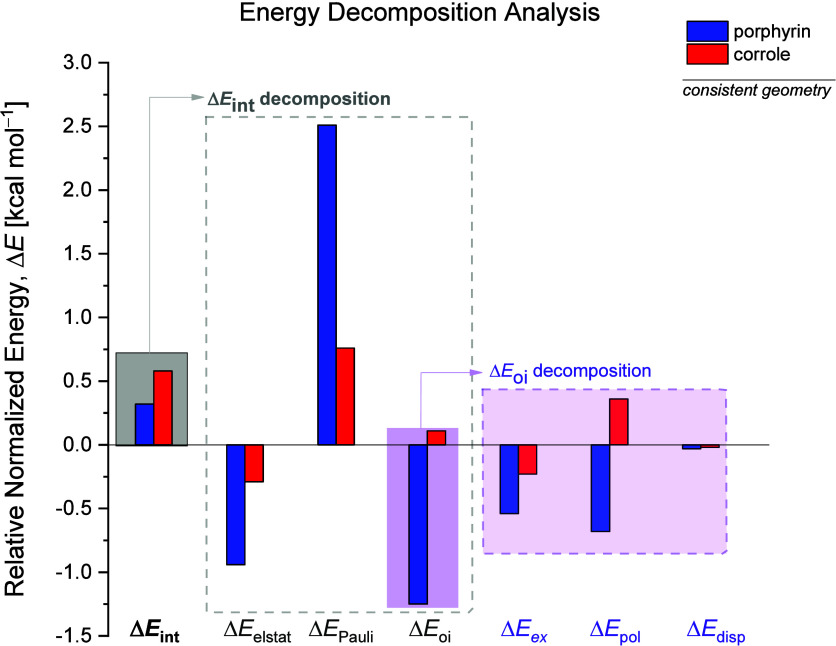
Energy decomposition analysis (EDA) of (PorBz)­B_2_OF_2_ (blue) and [(CorBz)­B_2_OF_2_]^−1^ (red) at the consistent geometry (BÔB = 164.7°). Fragment 1 corresponds to the B_2_OF_2_ bridge, and fragment 2 corresponds to the tetrapyrrolic macrocycle. The total interaction energy (Δ*E*_int_) is decomposed into electrostatic (Δ*E*_elstat_), Pauli repulsion (Δ*E*_Pauli_), and orbital interaction (Δ*E*_oi_) terms (gray). The orbital term is further decomposed into exchange (Δ*E*_ex_), polarization (Δ*E*_pol_), and dispersion (Δ*E*_disp_) contributions (purple). All energies are reported in kcal mol^–1^ relative to the ***t*_1_** isomer and were computed at the B3LYP-D3/6-31+G** level.

The porphyrin c.g. exhibits a normalized interaction energy relative to ***t***_**1**_ of 0.32 kcal mol^–1^ (Figure 5, blue bar in the highlighted gray box), while corrole shows a higher value of 0.58 kcal mol^–1^ (red bar). Decomposition of Δ*E*_int_ reveals that porphyrin has a more destabilizing Pauli repulsion than corrole, with Δ*E*_Pauli_ values of 2.51 and 0.76 kcal mol^–1^, respectively. The difference between these values can be expressed as ΔΔ*E*, being ΔΔ*E* = Δ*E*(porphyrin) – Δ*E*(corrole), yielding ΔΔ*E*_Pauli_ = 1.76 kcal mol^–1^, highlighting a stronger destabilizing contribution in the porphyrin system. Notably, however, the absolute Pauli repulsion in corrole ***t***_**1**_ is higher than that in porphyrin ***t***_**1**_ likely due to its contracted framework (Tables S5 and S6, Supporting Information). The relative and normalized values instead reflect the difference between the final state (c.g.) and the reference point (***t***_**1**_): porphyrin ***t***_**1**_ exhibits the smallest absolute Pauli repulsion, leading to a larger relative increase, whereas corrole shows higher absolute values in both states, resulting in a smaller relative Pauli contribution.

Nonetheless, this destabilization is effectively counterbalanced by more stabilizing electrostatic and orbital interactions. In both systems, the electrostatic component is stabilizing; however, its magnitude in corrole is approximately half of that in porphyrin (Δ*E*_elstat_ = −0.94 kcal mol^–1^ for porphyrin and Δ*E*_elstat_ = −0.29 kcal mol^–1^ for corrole), resulting in ΔΔ*E*_elstat_ = −0.65 kcal mol^–1^. Thus, electrostatic stabilization is more pronounced in porphyrin. The most significant energy difference between the two systems lies in the orbital interaction component. This term is stabilizing in porphyrin (Δ*E*_oi_ = −1.25 kcal mol^–1^) but slightly destabilizing in corrole (Δ*E*_oi_ = +0.11 kcal mol^–1^), resulting in ΔΔ*E*_oi_ = −1.36 kcal mol^–1^.

The trends in the orbital interaction energy can be understood through its further decomposition, as presented in eq 5 and detailed in Table S8. Figure 5 also illustrates this decomposition, emphasizing the individual components that comprise the orbital interaction term. For corrole, the primary destabilizing contribution arises from the polarization term (Δ*E*_pol_ = +0.36 kcal mol^–1^), whereas this term is stabilizing for porphyrin (Δ*E*_pol_ = −0.68 kcal mol^–1^). Polarization corresponds to the reorganization of the orbitals of one fragment in response to the presence of the other, leading to mixing between occupied and virtual orbitals.^55^

In the systems investigated here, the polarization energy reflects the energy released or required for the electronic densities of the macrocycle and the bridge to reorganize in the presence of one another. The positive polarization energy observed for corrole, in contrast to the negative value for porphyrin, indicates that the corrole macrocycle does not adapt in an energetically favorable way to the presence of the bridge. This behavior is consistent with the smaller cavity of the corrole, as previously discussed. As a result, the bridge is more effectively accommodated within the porphyrin cavity than within the contracted corrole framework, which is also reflected in the magnitude and sign of the covalent interaction term. Together, these results provide a qualitative picture that directly links macrocycle size to electronic adaptability: the reduced cavity of corrole limits its ability to reorganize electron density around the B_2_OF_2_ unit, thereby hindering stabilization and preventing accessible BAR processes.

A second factor contributing to the destabilizing Δ*E*_oi_ in corrole is the exchange energy. Although Δ*E*_ex_ is stabilizing in corrole (Δ*E*_ex_ = −0.24 kcal mol^–1^), its magnitude is 0.30 kcal mol^–1^ lower than that for porphyrin (Δ*E*_ex_ = −0.54 kcal mol^–1^). Like Pauli repulsion, exchange energy arises from antisymmetrization of the wave function. However, it is stabilizing because it reflects the exchange of electron spins required to satisfy the Pauli principle without inducing repulsion. The lower exchange stabilization observed in corrole reflects the structural differences between the systems, particularly the smaller cavity of the macrocycle. In porphyrins, the larger cavity allows for more effective accommodation of the bridge and better orbital overlap between the bridge and macrocycle. This enhanced overlap increases the likelihood of parallel-spin electron correlation, thereby making exchange stabilization more efficient.

Energy decomposition analysis results for the transition states (**TS**), shown in Tables S5 and S6, reveals the same trend observed in the c.g. analysis. The activation strain analysis (Figure 4b) indicates that the higher overall barrier in corrole is mainly associated with a more destabilizing interaction energy. Normalized EDA of both c.g. and **TS** confirms that this destabilization in corrole arises from a destabilizing covalent interaction term (Δ*E*_oi_), which is stabilizing in porphyrin, as well as from less effective electrostatic (Δ*E*_elstat_) stabilization compared to porphyrin. Since the total electronic energy—and thus the barrier for bond angle reflection—is directly influenced by Δ*E*_int_ (as shown in eq 2), the enhanced interaction energy in corrole directly contributes to its higher total electronic energy.

The *transoid*-BAR in corrole is kinetically limited due to a high energy barrier. Additionally, this *transoid* structure is thermodynamically less stable compared to its *cisoid* isomer, as previously discussed. Therefore, akamptisomerization could occur through the *cisoid* arrangement, if ***c*_2_** isomer is stable. Thus, we investigated modifications on the bridge components to enhance the preference for the *cisoid* configuration and potentially enabling the identification of the hypothetical *cisoid* akamptisomer—the ***c***_**2**_ form. To achieve this, we focused particularly on introducing favorable noncovalent interactions.

### *Cisoid* Preference: Stabilizing Bridge Interactions

In the *cisoid**c***_**2**_ form, both boron atoms lie within the macrocycle pseudoplane, which may result in increased in-plane distortion to accommodate these atoms. To address this potential structural limitation, we investigated the stabilization of this geometry by modifying bridge constituents, aiming to overcome the distortion through favorable interaction energies. Specifically, we explored the role of long-range noncovalent interactions, which—depending on their nature—can exert stabilizing or destabilizing effects and are known to influence the structural organization of molecules and supramolecular assemblies by favoring specific spatial arrangements.^56^ To this end, a new set of systems featuring the B_2_OY_2_ bridge was selected, aiming to introduce stabilizing Y_1_•••Y_2_ interactions, with Y_1_ = Y_2_ = −F (**a**) serving as the reference system, in the same porphyrin (**1**) and corrole (**2**) frameworks: (PorBz)­B_2_OY_2_ and [(CorBz)­B_2_OY_2_]^−^.

To explore the potential for stabilizing hydrogen bonds between Y_1_•••Y_2_ groups, we selected the following systems: Y_1_ = Y_2_ = −OH (**b**); Y_1_ = −NH_2_ and Y_2_ = −CO_2_H (**c**); and Y_1_ = −N­(^*t*^Bu)_2_ with Y_2_ = −CO_2_H (**d**). Compound **b** has been previously identified experimentally in *transoid*-like porphyrin derivatives.^57^ For potential π•••π interactions, we considered Y_1_ = Y_2_ = −Ph (**e**) and Y_1_ = Y_2_ = −C≡C–C≡C–Ph (**f**). Compound **e** has been reported in combination with −OH groups (Y_1_ = −OH and Y_2_ = −Ph) in *transoid* configurations,^57^ whereas **f** was chosen based on the formation of a *cisoid **c***_**2**_-like structure previously observed in porphyrins bearing the same alkynyl bridging unit, where substitution of the phenyl group with a fullerene (−C_60_) led to stabilization of this configuration.^37^

Unfortunately, after several attempts, it was not possible to obtain the ***c***_**2**_ form as a minimum energy structure even upon modification of the Y substituents. Along the optimization procedure, as observed for **a** (Y_1_ = Y_2_ = −F), all ***c***_**2**_ structures converged to ***c***_**1**_. The obtained results for porphyrin (**1a**–**f**) and corrole (**2a**–**f**) are summarized in Table 1, where the reported free energy differences (Δ*G*) are calculated relative to the ***t***_**1**_ isomers at different levels of theory.

**Table 1:** Gibbs Free Energy (kcal mol^–1^) of the *Cisoid **c***_**1**_ Stereoisomer Relative to ***t*_1_** (Δ*G* = *G**t***_**1**_ – *G**c***_**1**_) for (PorBz)­B_2_OY_2_ (**1a**–**f**) and [(CorBz)­B_2_OY_2_]^−1^ (**2a**–**f**) with Different Y Substituents^a^

	6-31+G**	def2-TZVP		
Evaluated system	B3LYP-D3	B3LYP-D3	M06-2X	CAM-B3LYP
Porphyrin (PorBz)B_2_OY_2_				
**1a**	6.6	6.5	6.9	7.2
**1b**	4.8	4.7	4.8	4.8
**1c**	2.6	2.5	2.9	3.7
**1d**	4.5	4.6	6.5	7.4
**1e**	0.2	–0.7	–0.9	–1.2
**1f**	10.0	9.0	9.6	9.2
Corrole [(CorBz)B_2_OY_2_]^−^				
**2a**	–14.8	–16.3	–15.3	–14.4
**2b**	–16.1	–15.5	–16.8	–14.7
**2c**	–21.9	–22.6	–22.5	–21.3
**2d**	–21.1	–20.0	–19.9	–18.4
**2e**	–25.1	–26.2	–26.5	–25.1
**2f**	–12.3	–14.1	–13.9	–13.2

a Optimization of the ***c*_2_** structure did not yield a minimum and always converged to ***c*_1_**. **a**: Y_2_ = Y_2_ = −F; **b**: Y_1_ = Y_2_ = −OH; **c**: Y_2_ = −NH_2_, Y_2_ = −CO_2_H; **d**: Y_1_ = −N­(^*t*^Bu)_2_, Y_2_ = −CO_2_H; **e**: Y_1_ = Y_2_ = −Ph; **f**: Y_1_ = Y_2_ = −C≡C–C≡C–Ph.

Despite the inability to locate ***c***_**2**_ as a stable structure, general stabilization of the *cisoid* (***c***_**1**_) form was observed for **1a**–**e** and **2a**–**e**, with a stronger effect in the corrole derivatives (**2a**–**e**). This trend may arise either from stabilizing noncovalent interactions, which are geometrically accessible only in the *cisoid* (***c***_**1**_) isomer, or from steric and distortion effects that reduce the stability of the *transoid* (***t***_**1**_) configuration.

The **2e** system stands out due to the most significant stabilization of the *cisoid* form, with a Δ*G* value of approximately −26 kcal mol^–1^. For porphyrin, the **1e** *cisoid* and *transoid* isomers become almost isoenergetic, with Δ*G* values ranging from −1.2 to −0.7 kcal mol^–1^ across different theoretical levels. In this case, no evident stabilization arises from direct ring interactions, as the substituents are positioned too far from the macrocycle center (approximately 6 Å) to contribute significantly through noncovalent interactions (as shown in Figure S7 in the Supporting Information file). When other geometric parameters were evaluated—particularly the angle of the anchored bridge and the tetragonal in-plane distortion—it was found that the *transoid*/*cisoid* preference is possibly correlated with the tetragonal in-plane distortion Δ*d*_N•••N_ of the macrocyclic cavity. The *transoid* arrangement for **e** (Y_1_ = Y_2_ = −Ph) exhibits a greater distortion compared to the *cisoid* form for both macrocycles. Specifically, Δ*d*_N•••N_ values are 0.72 (**1e**) and 0.59 (**2e**) Å for the *transoid* configuration and 0.50 (**1e**) and 0.40 (**2e**) Å for the *cisoid* configuration. These trends are summarized in Figure S7a, which illustrates the structural distortion of the *transoid **t***_**1**_ and *cisoid **c***_**1**_ isomers. A similar behavior has been reported in phthalocyanine- and porphyrazine-type porphyrinoid systems, where both *transoid* and *cisoid* configurations were isolated and experimentally characterized. In those cases, the observed stereochemistry was correlated with the degree of cavity distortion.^15^

Another noteworthy system is **f** (Y_1_ = Y_2_ = −C≡C–C≡C–Ph), which stands out by exhibiting the opposite trend to **e**. Compared to the reference system **a**, **f** shows a relative stabilization of the *transoid* isomer, with ΔΔ*G* = 3.4 (**1f**) kcal mol^–1^ and 2.5 (**2f**) kcal mol^–1^, where ΔΔ*G* = Δ*G*(**f**) – Δ*G*(**a**). Although **f** and **e** exhibit similar tetragonal in-plane distortion, the alkynyl bridge in **f** enables a T-shaped π•••π interaction between the macrocycle pseudoplane and the phenyl substituent in the *transoid* form, at a short distance of 3.2 Å (see Figure S7b, in the Supporting Information file). This favorable noncovalent interaction overcomes the destabilizing effect of the distortion, favoring the *transoid* configuration.

Finally, an unprecedented form of conformational isomerism was identified for system **1c** (Y_1_ = −NH_2_, Y_2_ = −CO_2_H) in porphyrins. This phenomenon involves two distinct *cisoid* structures that interconvert via the reflection of the BÔB bond angle above the porphyrin pseudoplane (Figure 6). The interconversion arises from the possibility of forming two alternative hydrogen bonds: (Y_2_)­COOH•••O­(bridge) or (Y_2_)­COOH•••NH_2_(Y_1_). This flexibility enables BÔB angle reflection, yielding a second *cisoid* isomer (Δ*G* = 7.3 kcal mol^–1^) through a conformational transition with an associated low barrier of Δ*G* ^‡^ = 7.9 kcal mol^–1^. Although endergonic, this process is kinetically accessible, resulting in a fluxional equilibrium in which both *cisoid* forms coexist, albeit biased toward the more stable ***c***_**1**_ structure. Since this isomer does not correspond to the typical ***c*** **_2_** configuration—i.e., it does not involve the BAR across the pseudoplane with the −BY group positioned in-plane—we designated it as the *c*_***x***_ isomer.

**Figure 6:**
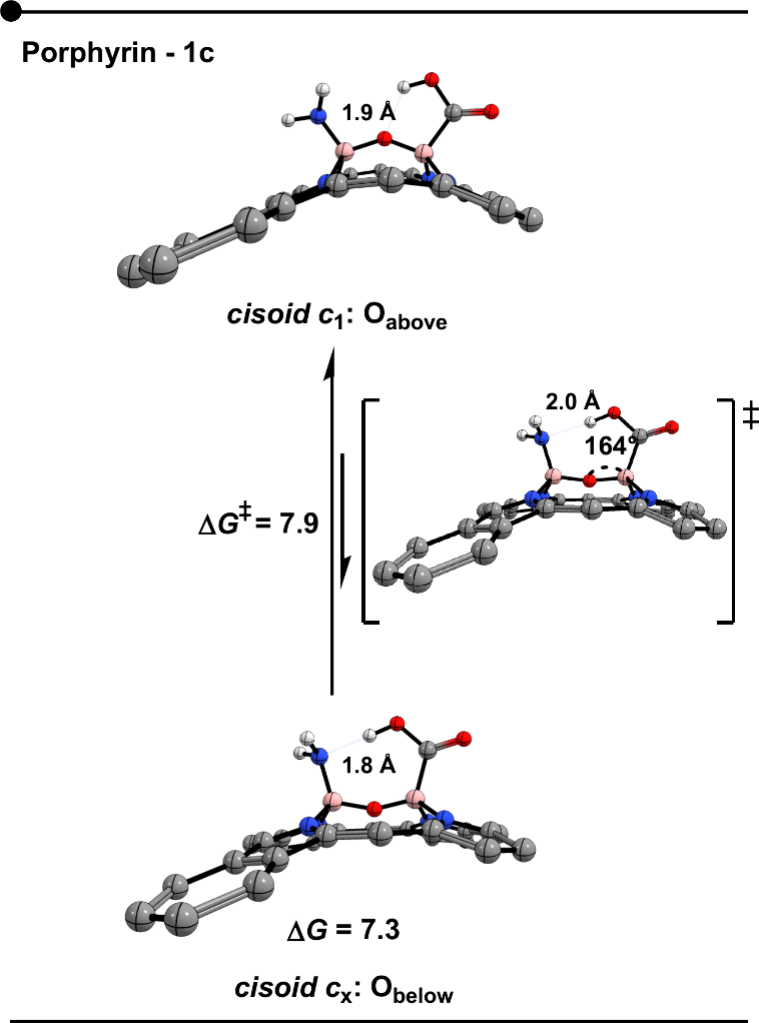
Optimized structures (B3LYP-D3/6-31+G**) of porphyrins **1c** structures (B (Y_1_ = −NH_2_, Y_2_ = −CO_2_H). Energy reported is in kcal mol^–1^.

To the best of our knowledge, this is the first theoretical evidence of a conformational process occurring above the porphyrin pseudoplane. Although the energy barrier is low—preventing the isolation of individual conformers—this dynamic behavior opens a potential pathway for designing new building blocks for the development of more robust materials and boron-based complexes. These findings pave the way for future studies exploring conformational control in porphyrinoid frameworks and their applications in molecular devices and responsive systems, even though it does not involve the *cisoid*-BAR process, which, according to our system, remains inaccessible.

## 4 Conclusions

Porphyrinoid macrocycles are useful in the development of functional materials with broad applications ranging from biological systems to advanced technologies. Among them, main-group coordination-driven porphyrins have gained attention due to the incorporation of *p*-block elements, such as boron. Boron-bridged complexes featuring the B_2_OF_2_ bridges have recently drawn interest for exhibiting a novel form of conformational isomerism—akamptisomerism—which arises from bond angle reflection (BAR) of the B–O–B moiety in low-symmetry porphyrins. The incorporation of this boron bridge has been reported in several porphyrinoid scaffolds, suggesting the potential for applications as molecular switches. In this work, we employed density functional theory to investigate BAR processes in porphyrin and its contracted analogue, corrole, to understanding how macrocyclic size influences: the preferred isomeric configuration (i.e., the orientation fluorine in the B_2_OF_2_ unit), the electronic and structural factors that controls akamptisomerism, and its feasibility in corrole.

Regarding the anchoring of the B_2_OF_2_ bridge, our B3LYP-D3/6-31+G** calculations, which are consistent with experimental evidence, showed a preference for the *transoid* isomer in porphyrin, with Δ*G* = 7 kcal mol^–1^ compared to that of *cisoid*. In contrast, corrole favors the *cisoid* isomer by Δ*G* ≈ 15 kcal mol^–1^. Geometric analysis suggests that preference for the *cisoid* structure, for corrole, is associated with reduced tetragonal in-plane distortion. In porphyrin, however, the *transoid* isomer presents higher distortion. Natural bond orbital (NBO) analysis revealed that in porphyrin orbital overlap and N → B donor interactions stabilize the *transoid* configuration despite its higher distortion. Although the same interaction is also present in corrole, it is insufficient to overcome the geometric constraints imposed by the contracted macrocycle, making the *cisoid* form more stable.

To assess the feasibility of BAR processes, we focused on the *transoid* isomer since one of the *cisoid* akamptisomers could not be located as a minimum on the potential energy surface of either macrocycle. Across different functionals and basis set calculations, porphyrin consistently exhibited an accessible BAR barrier (Δ*G* ^‡^ 25–28 kcal mol^–1^), agreeing with previous observations, whereas corrole displayed a prohibitively high barrier (*transoid*-BAR Δ*G* ^‡^ > 43 kcal mol^–1^), rendering the process inaccessible. Activation strain analysis indicated that the barrier is primarily controlled by the bridge•••macrocycle interaction rather than strain, and energy decomposition analysis revealed that the destabilization in corrole originates from the orbital interaction term. Specifically, while the polarization contribution stabilizes the transition state in porphyrin, it becomes destabilizing in corrole, showing that its electronic structure does not adapt favorably to the B_2_OF_2_ bridge and thereby prevents akamptisomerism.

Finally, to identify a potential BAR process in the *cisoid* configuration, a distinct set of bridge substitutions was explored to overcome the intrinsic instability of the *cisoid* form by introducing stabilizing noncovalent interactions. Replacing the fluorine atoms with groups capable of hydrogen bonding or π-interactions generally increased the stability of the *cisoid* form compared to that of its *transoid* counterpart. However, this stabilization was still insufficient to locate both *cisoid* isomers on the potential energy surface, thus preventing the observation of a *cisoid*-BAR process.

Our findings deepen our understanding of how macrocycle size, particularly cavity dimensions, determines both the configuration of the anchored B–O–B bridge and the overall conformational behavior, achieved through the direct comparison of porphyrin with its ring-contracted analogue, corrole. Within this framework, we rationalized the newly identified conformational isomerism, akamptisomerism, demonstrating that the macrocycle plays a decisive role in enabling or suppressing bond angle reflection (BAR) processes. In particular, the reduced cavity of corrole prevents energetically accessible BAR processes. This qualitative structure–reactivity relationship provides a general guideline for predicting akamptisomerism across diverse porphyrinoid architectures. Beyond advancing structural and electronic insights, recognizing akamptisomerism as a conceptually rich stereochemical feature may also inspire the rational design of future functional materials.

## Supplementary Material



## Data Availability

Requests for materials should be addressed to rodolfofiorot@id.uff.br.

## Notes

Details of stereodescriptor determinations, additional electronic and geometric information, and the Cartesian coordinates for all the evaluated structures (PDF)
