# Supplementary material for: Akamptisomerism Beyond Porphyrins: Bond Angle Reflection and Stereochemical Divergences in Corrole- and Porphyrin-Anchored B–O–B Bridges
Source: Inorg Chem. 2025 Dec 22;65(1):416–27. doi: 10.1021/acs.inorgchem.5c04528 (PMC12801324; doi:10.1021/acs.inorgchem.5c04528)
Supplement: Supplementary file 1 [file ic5c04528_si_001.pdf]

SUPPORTING INFORMATION MATERIAL

Akamptisomerism Beyond Porphyrins: Bond Angle Reflection and Stereochemical Divergences in Corrole- and Porphyrin-Anchored B–O–B Bridges

Karine N. de Andrade[1], Patrick L. L. Rocha[1], Daniella B. de Miranda[2], Natalia M. Raffaeli[1], Gláucio B. Ferreira[2], Rodolfo G. Fiorot[1,*]

[1] Department of Organic Chemistry, Chemistry Institute, Universidade Federal Fluminense (UFF), Outeiro de São João Batista, 24020-141, Niterói, RJ, Brazil

[2] Department of Inorganic Chemistry, Chemistry Institute, Universidade Federal Fluminense (UFF), Outeiro de São João Batista, 24020-141, Niterói, RJ, Brazil

*Corresponding author: Rodolfo G. Fiorot - rodolfofiorot@id.uff.br

TABLE OF CONTENTS

1. Tetrahedral in-plane distortion 2
2. Corrole anchoring modes: parallel *vs*. perpendicular 3
2. BAR processes: influence of theoretical level 5
3. Relative stability: natural bond orbital (NBO) analysis 6
4. Intrinsic Reaction Coordinates (IRC) graphs 9
5. Energy decomposition analysis (EDA) 9
6. *Cisoid* preference: stabilizing bridge interactions 11
7. Matrices of optimized structures 12

1. Tetrahedral in-plane distortion

The in-plane distortion of the macrocycle was evaluated through calculations of tetragonal in-plane distortion ($\Delta\bar{d}_{N\cdots N}$), obtained from a direct comparison with the optimized free-base structures of each porphyrinoid, using the neutral monobenzo-fused porphyrin (**PorBz**) and the anionic form of monobenzo-fused corrole (**CorBz**) — Figure S1. The anionic corrole was chosen to maintain the overall –1 charge in both the free base and the B_2OF_2-complexed form. Since corrole displays two distinct N•••N distances, we report here the average values for both macrocycles, calculated as follows:

$$\Delta\bar{d}_{N\cdots N} = \bar{d}_{N\cdots N}[B_2OF_2\text{-complex}] - \bar{d}_{N\cdots N}[\text{free base}] \quad (1)$$

where $\bar{d}_{N\cdots N}$ corresponds to the average of the N•••N distances in free base and the B_2OF_2-anchored macrocycle:

$$\bar{d}_{N\cdots N} = \frac{dN1\bullet\bullet\bullet N4 + dN2\bullet\bullet\bullet N3}{2} \quad (2)$$

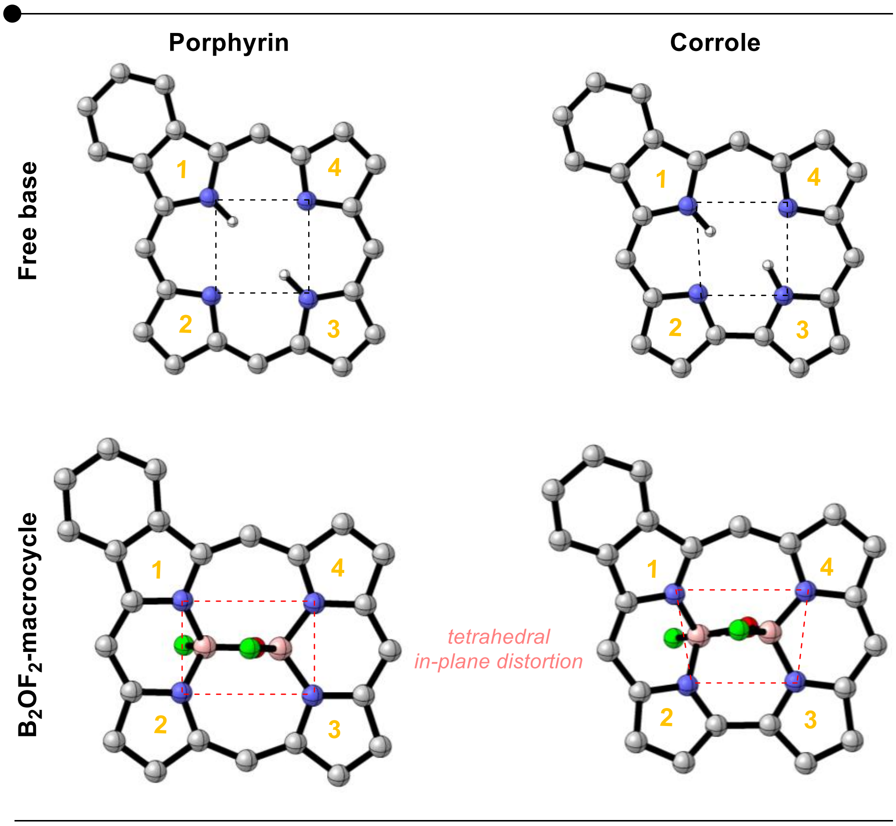

Figure S1: Structures optimized (*transoid*) at the B3LYP-D3/6-31+G* level for in-plane distortion analysis: free-base (referential) and B_2OF_2-anchored macrocycles.

2. Corrole anchoring modes: parallel *vs*. perpendicular

Corrole presents two distinct anchoring modes for the B_2OF_2 bridge: parallel, referred to as (Cor)‖B_2OF_2, and perpendicular, named (Cor)⊥B_2OF_2, relative to the interconnected pyrrole rings (Figure S2a).

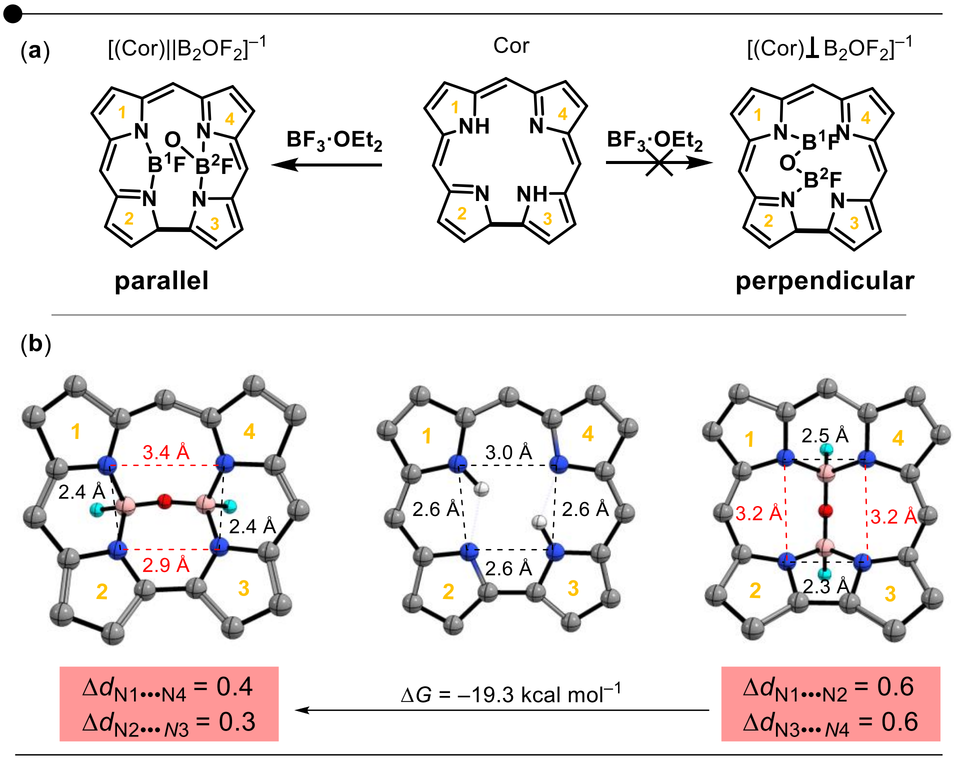

Figure S2: (**a**) Schematic representation of the parallel (Cor)‖B_2OF_2 and perpendicular (Cor)⊥B_2OF_2 anchoring of B_2OF_2 bridge. (**b**) Optimized structures at the B3LYP-D3/6-31+G** level, highlighting the N•••N distances (in Å) and the computed of the tetragonal in-plane distortion Δd(N•••N) = d(N•••N)[B_2OF_2] − d(N•••N)[free base]. Reported energy difference between both forms, calculated as ΔG = (Cor)‖B_2OF_2 – (Cor)⊥B_2OF_2.

The parallel arrangement is significantly more stable (by 19.3 kcal mol^{-1}) than the perpendicular one, which present a reduced tetragonal in-plane distortion of the macrocyclic cavity relative to the free-base structure. This distortion, as cited above, is quantified by $\Delta d_{N\cdots N)}$ (Å), defined as the difference between the distances of the pyrrole nitrogen atoms (N•••N) aligned with the B•••B axis in the macrocycle bearing the anchored B_2OF_2 bridge and the corresponding distances in the free-base macrocycle (Equation 3):

$$\Delta d_{N\cdots N} = d(N\cdots N)[B_2OF_2] - d(N\cdots N)[\text{free-base}] \quad (3)$$

SUPPORTING INFORMATION MATERIAL

As shown in Figure S2b, the parallel arrangement exhibits a smaller distortion, $\Delta d_{N\cdots N}$, compared to the perpendicular configuration. This trend was consistently observed across calculations performed at different levels of theory, using the def2-TZVP basis set combined with the B3LYP-D3, M06-2X, and CAM-B3LYP functionals (Table S1). In all cases, the parallel arrangement was energetically favored, with ΔG values ranging from 17.3 to 19.3 kcal mol^{-1}. Negative values of $\Delta d_{N\cdots N}$ indicate a contraction in the distance between the two evaluated nitrogen atoms, while positive values reflect an expansion. These distortions depend on the orientation of the B_2OF_2 bridge relative to the macrocycle.

Table S1: Geometric parameters evaluated for the parallel and perpendicular anchoring of the B_2OF_2 bridge in corroles. Interatomic distances (d) are reported in Å, and trapezoidal area, in Å^2. Gibbs free energy change $\Delta G = G_{\parallel} - G_{\perp}$ is given in kcal mol^{-1}.

	6-31+G**	def2-TZVP		
	B3LYP-D3	B3LYP-D3	CAM-B3LYP	M06-2X
		(Cor)∥B_2OF_2		
$d_{N1\cdots N2}$	2.43	2.43	2.43	2.43
$d_{N2\cdots N3}$	2.93	2.92	2.92	2.91
$d_{N3\cdots N4}$	2.43	2.43	2.42	2.43
$d_{N4\cdots N1}$	3.39	3.38	3.39	3.37
$\Delta d_{N1\cdots N2}$	–0.22	–0.21	–0.22	–0.23
$\Delta d_{N2\cdots N3}$	0.36	0.36	0.37	0.37
$\Delta d_{N3\cdots N4}$	–0.21	–0.20	–0.21	–0.22
$\Delta d_{N4\cdots N1}$	0.41	0.42	0.44	0.45
Area[a]	7.6	7.6	7.6	7.6
		(Cor)⊥B_2OF_2		
$d_{N1\cdots N2}$	3.20	3.20	3.22	3.18
$d_{N2\cdots N3}$	2.28	2.28	2.28	2.28
$d_{N3\cdots N4}$	3.20	3.21	3.22	3.18
$d_{N4\cdots N1}$	2.51	2.50	2.49	2.49
$\Delta d_{N1\cdots N2}$	0.55	0.56	0.58	0.53
$\Delta d_{N2\cdots N3}$	–0.28	–0.28	–0.27	–0.26
$\Delta d_{N3\cdots N4}$	0.57	0.57	0.59	0.54
$\Delta d_{N4\cdots N1}$	–0.47	–0.47	–0.46	–0.43
Area[a]	7.7	7.7	7.7	7.6
$\Delta G = G_{\parallel} - G_{\perp}$	17.3	19.3	18.8	18.4

[a] trapezoidal area, computed with $\frac{1}{2}(d_{N4\cdots N1}+d_{N2\cdots N3})d_{N1\cdots N2}$ in Å^2.

2. BAR processes: influence of theoretical level

The BAR processes were also evaluated at different theoretical levels, with full optimizations and frequency calculations performed at the levels listed in Table S2. These results show only a minor influence on the overall profile, consistently maintaining the trend identified for energy barrier and isomers stability.

Table S2: Energy variation (in kcal mol^{-1}) for the bond angle reflection processes at different theoretical levels. Reported values relative to $\boldsymbol{t_1}$ isomer.

	6-31+G**	def2-TZVP		
	B3LYP-D3	B3LYP-D3	M06-2X	CAM-B3LYP
	Porphyrin (PorBz)B_2OF_2			
TS ($\boldsymbol{t_1}$→$\boldsymbol{t_2}$)	25.4	26.5	27.7	27.1
$\boldsymbol{t_2}$	–0.3	–0.3	–0.4	–0.5
$\boldsymbol{c_1}$ [a]	6.6	6.5	7.2	6.9
	Corrole [CorBz(B_2OF_2)]$^-$			
**TS ($\boldsymbol{t_1}$→$\boldsymbol{t_2}$)**	43.4	44.3	46.8	45.0
$\boldsymbol{t_2}$	–0.9	–0.9	–1.2	–1.1
$\boldsymbol{c_1}$ [a]	–14.8	–15.6	–14.4	–15.3

[a] The $\boldsymbol{c_2}$ isomer converged to $\boldsymbol{c_1}$ without an associated energy barrier.

All results discussed in the main text were obtained in the gas phase. To examine the influence of the medium, single point calculations were performed at the gas-phase optimized geometries using the IEFPCM implicit solvation model with dichloromethane (CH_2Cl_2) as solvent, matching the experimental conditions typically used for B_2OF_2-porphyrinoid complexes. As summarized in Table S3, the qualitative trends remained unchanged at B3LYP-D3/6-31+G**: porphyrin retains a preference for the *transoid* isomer, with accessible energy barrier, whereas corrole remains stabilized in the *cisoid* form, for which the *transoid*-BAR pathway becomes prohibitively high in energy.

Table S3: Energy variation (in kcal mol^{-1}) for the bond-angle reflection processes obtained from single-point calculations with implicit solvation (IEFPCM, solvent = dichloromethane) on structures optimized at the B3LYP-D3/6-31+G* level.

	TS ($t_1 \rightarrow t_2$)	t_2	c_1
Porphyrin (PorBz)B_2OF_2	25.9	–0.4	5.1
Corrole [CorBz(B_2OF_2)]$^-$	44.8	–0.9	–15.5

3. Relative stability: natural bond orbital (NBO) analysis

Table S4. Average bond lengths and NBO energies ($\bar{E}_{NBO}$) for B–N and B–O bonds in (PorBz)B_2OF_2 and [(CorBz)B_2OF_2]$^{-1}$ (t_1 and c_1 isomers).

**Bond**	**Bond length (Å)**	**Average NBO Energy (kcal mol^{-1})**
	**Porphyrin**	
	**t_1 (preferred isomer)**	
N–B	1.56	236.3
O–B	1.39	39.8
	**c_1**	
N–B	1.60	193.2
O–B	1.37	49.3
	**Corrole**	
**Bond**	**Bond length (Å)**	**Average NBO Energy (kcal mol^{-1})**
	**t_1**	
N–B	1.54	246.2
O–B	1.40	35.6
	**c_1 (preferred isomer)**	
N–B	1.58	208.2
O–B	1.38	42.9

Table S5. Average bond lengths and NBO normalized* energies ($\bar{E}_{NBO}$) for B–N and B–O bonds in (PorBz)B_2OF_2 and [(CorBz)B_2OF_2]$^{-1}$ (t_1 and c_1 isomers).

**Bond**	**Bond length (Å)**	**NBO Normalized Energy (kcal mol^{-1})**
	**Porphyrin**	
	**t_1**	
N-B	1.564	5.0
O-B	1.391	0.8
	**c_1**	
N-B	1.596	4.1
O-B	1.368	1.0
	**Corrole**	
**Bond**	**Bond length (Å)**	**NBO Normalized Energy (kcal mol^{-1})**
	**t_1**	
N-B	1.536	5.5

O-B	1.399	0.8
	***c*₁**	
N-B	1.578	4.6
O-B	1.378	1.0

†Corrole: 45 atoms; porphyrin: 47 atoms.

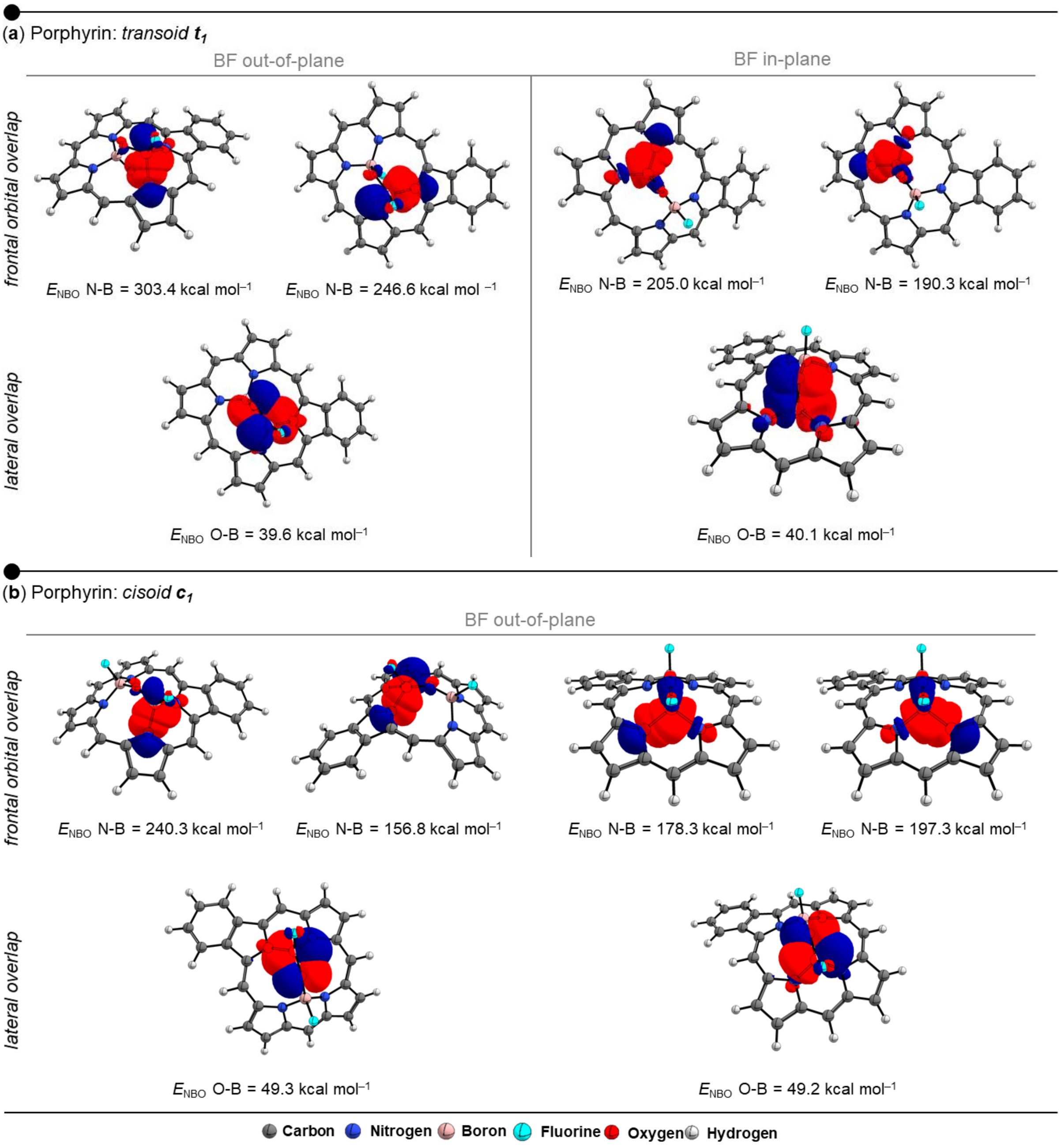

Figure S4. Natural Bond Orbitals computed at the B3LYP-D3/6-31+G** level of the $(PorBz)B_2OF_2$ akamptisomer: (**a**) *transoid* $\boldsymbol{t_1}$, and (**b**) *cisoid* $\boldsymbol{c_1}$. Surfaces plotted with an isovalue of 0.04.

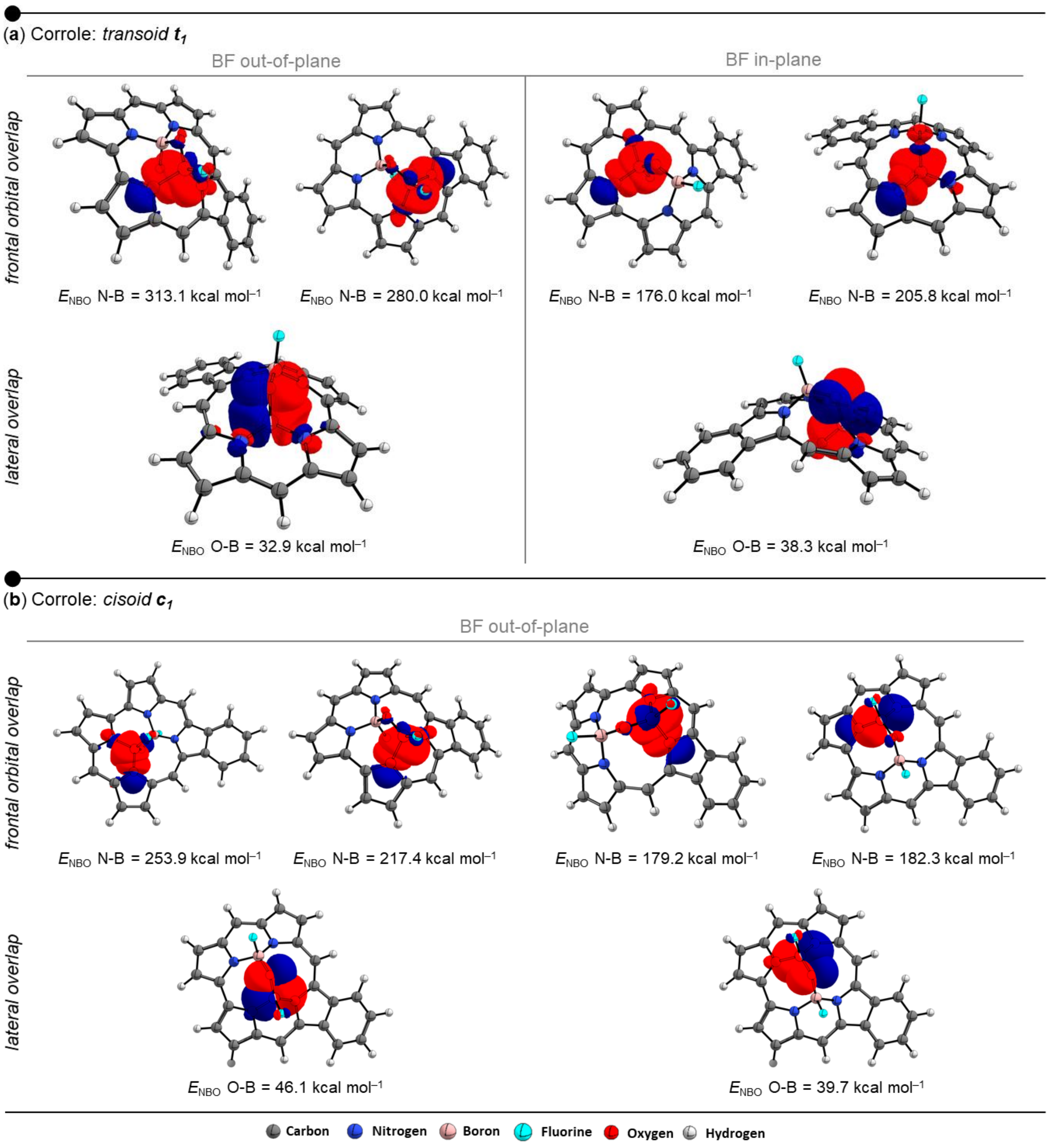

Figure S5. Natural Bond Orbitals computed at the B3LYP-D3/6-31+G** level of the $[(CorBz)B_2OF_2]^{-1}$ akamptisomer: (**a**) *transoid* $\boldsymbol{t_1}$, and (**b**) *cisoid* $\boldsymbol{c_1}$. Surfaces plotted with an isovalue of 0.04.

4. Intrinsic Reaction Coordinates (IRC) graphs

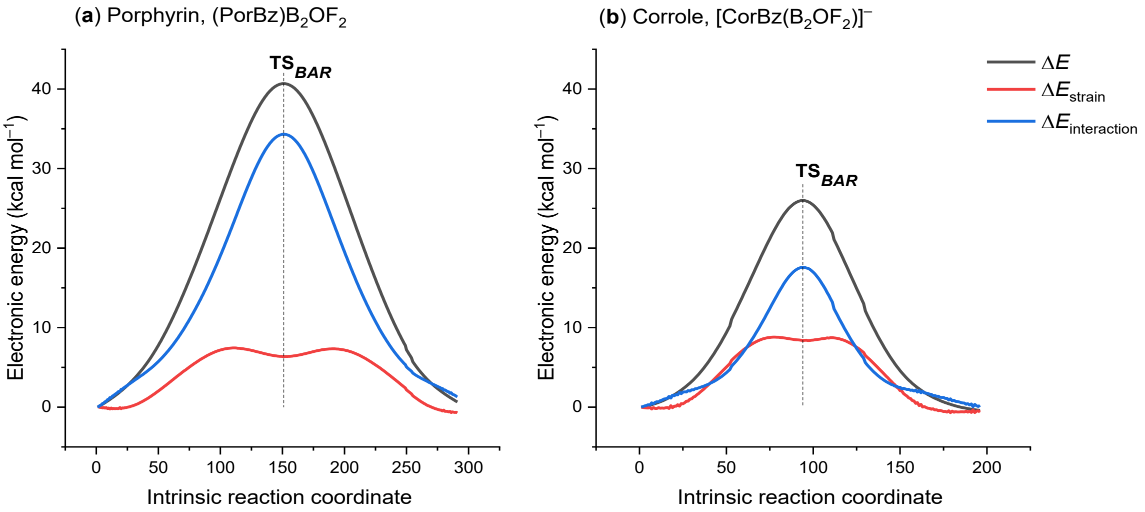

Figure S6: Activation strain diagram relative to the bond angle reflection for the B_2OF_2 bridge (fragment 1) and the tetrapyrrolic macrocycle (fragment 2) along the intrinsic reaction coordinate (IRC) of (**a**) porphyrin, and (**b**) corrole. Black curves: total electronic energy (ΔE); red: distortion energy (ΔE_{strain}); blue interaction energy (ΔE_{int}). The x-axis represents the dimensionless reaction coordinate as computed by default in Gaussian 09.

5. Energy decomposition analysis (EDA)

Table S6. Energy decomposition analysis (EDA) computed at the B3LYP-D3/6-31+G** level of $(PorBz)B_2OF_2$ and $[(CorBz)B_2OF_2]^{-1}$: absolute and relative energies, reported in kcal mol^{-1}.

				**Absolute Energy**				
	**Porphyrin**				**Corrole**			
	ΔE_{int}	$\Delta E_{eslstat}$	ΔE_{Pauli}	ΔE_{oi}		ΔE_{int}	ΔE_{elstat}	ΔE_{Pauli}	ΔE_{oi}
***t*₁**	–338.29	–564.34	1666.77	–1440.72	***t*₁**	–327.28	–628.95	1831.40	–1529.73
c.g.	–323.10	–608.57	1784.94	–1499.47	***c.g.***	–301.30	–642.08	1865.47	–1524.69
**TS**	–321.05	–611.02	1793.03	–1503.06	**TS**	–288.42	–650.51	1886.18	–1524.09
				**Relative Energy**				
	ΔE_{int}	ΔE_{elstat}	ΔE_{Pauli}	ΔE_{oi}		ΔE_{int}	ΔE_{elstat}	ΔE_{Pauli}	ΔE_{oi}
c.g.	15.19	–44.23	118.17	–58.75	***c.g.***	25.98	–13.13	34.07	5.04
**TS**	17.24	–46.68	126.26	–62.34	**TS**	38.86	–21.56	54.78	5.64

ΔE_{elstat} = Electrostatic energy; **ΔE_{oi}** = Orbital Interaction energy; **ΔE_{Pauli}** = Pauli repulsion; **ΔE_{int}** = Interaction energy.

Table S7. Normalized† energy decomposition analysis (EDA) computed at the B3LYP-D3/6-31+G** level of (PorBz)B_2OF_2 and [(CorBz)B_2OF_2]$^{-1}$: absolute and relative energies, reported in kcal mol^{-1}.

Absolute Energy									
	Porphyrin					Corrole			
	ΔE_{int}	ΔE_{elstat}	ΔE_{Pauli}	ΔE_{oi}		ΔE_{int}	ΔE_{elstat}	ΔE_{Pauli}	ΔE_{oi}
***t*1**	–7.20	–12.01	35.46	–30.65	***t*1**	–7.27	–13.98	40.70	–33.99
c.g.	–6.87	–12.95	37.98	–31.90	***c.g.***	–6.70	–14.27	41.45	–33.88
**TS**	–6.83	–13.00	38.15	–31.98	**TS**	–6.41	–14.46	41.92	–33.87
**Relative Energy**									
	ΔE_{int}	ΔE_{elstat}	ΔE_{Pauli}	ΔE_{oi}		ΔE_{int}	ΔE_{elstat}	ΔE_{Pauli}	ΔE_{oi}
c.g.	0.32	–0.94	2.51	–1.25	***c.g.***	0.58	–0.29	0.76	0.11
**TS**	0.37	–0.99	2.69	–1.33	**TS**	0.86	–0.48	1.22	0.13

†Corrole: 45 atoms; porphyrin: 47 atoms.

Table S8. Normalized* Energy Decomposition Analysis (EDA) computed at the B3LYP-D3/6-31+G** level of Orbital Interaction (ΔE_{oi}) contribution of (PorBz)B_2OF_2 and [(CorBz)B_2OF_2]$^{-1}$, reported in kcal mol^{-1}.

Relative Normalized Energy, ΔE_{oi}			
**Porphyrin**	$\boldsymbol{\Delta E_{ex}}$	$\boldsymbol{\Delta E_{pol}}$	$\boldsymbol{\Delta E_{disp}}$
c.g.	–0.54	–0.68	–0.03
**TS**	–0.57	–0.73	–0.03
**Corrole**	$\boldsymbol{\Delta E_{ex}}$	$\boldsymbol{\Delta E_{pol}}$	$\boldsymbol{\Delta E_{disp}}$
c.g.	–0.23	0.36	–0.01
**TS**	–0.35	0.50	–0.02

†Corrole: 45 atoms; porphyrin: 47 atoms.

6. *Cisoid* preference: stabilizing bridge interactions

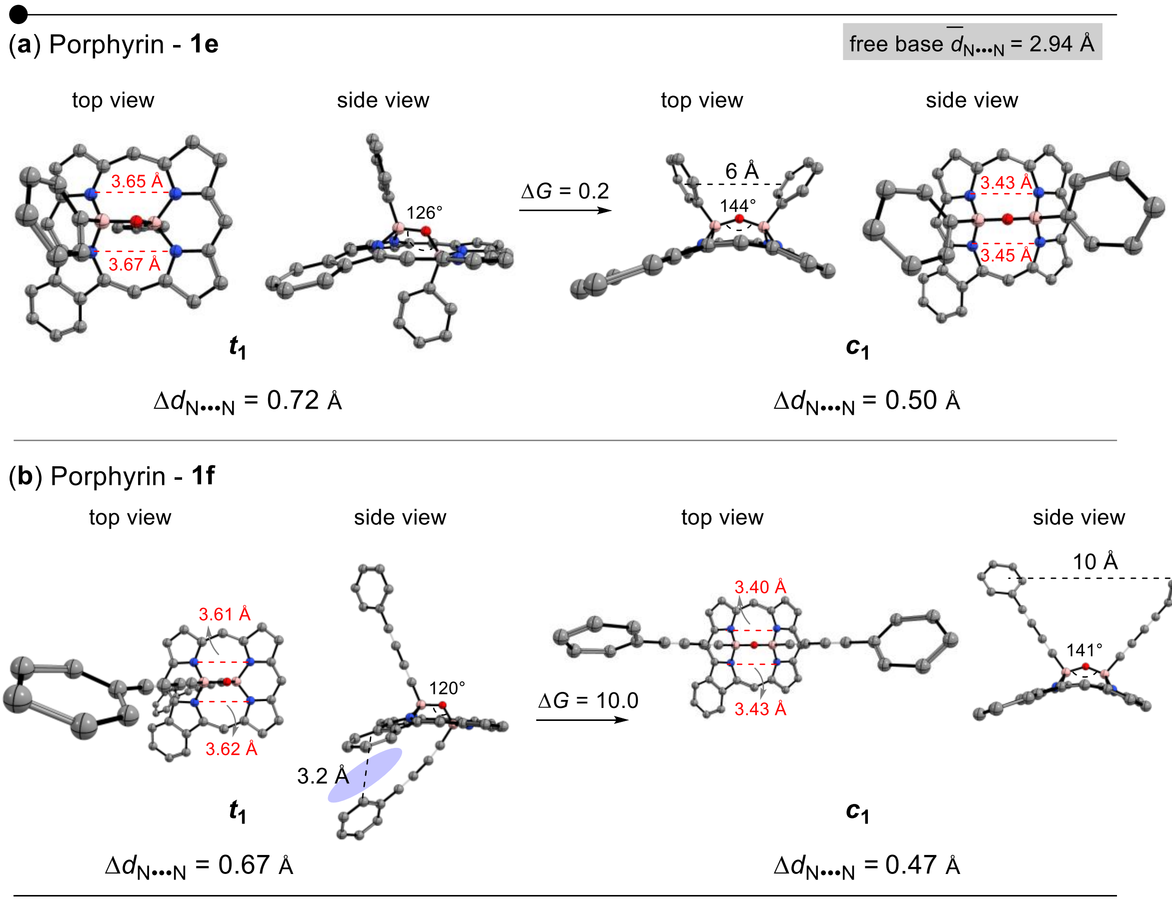

Figure S7: Optimized structures at the B3LYP-D3/6-31+G** level for porphyrins **1e** ($Y_1 = Y_2 =$ –Ph) and **1f** ($Y_1 = Y_2 =$ –C≡C–C≡C–Ph). Tetragonal in-plane distortion $\Delta d_{N\cdots N}$, in Å, represented relative to the free base. Energy reported in kcal mol^{-1}.

7. Matrices of optimized structures

Colour scheme: carbon (gray), hydrogen (white), nitrogen (blue), fluorine (cyan), oxygen (red).

Cor_C2v – Unsubstituted C_{2v} symmetry corrole reference structure

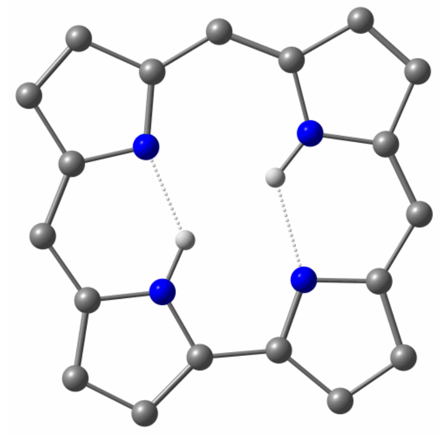

1	-3.940494000	-3.277317000	0.001221000
1	3.028492000	3.896923000	0.000885000
1	-5.137721000	-0.866177000	0.000880000
1	4.813960000	1.879774000	0.000736000
1	1.196168000	-4.960993000	0.000349000
1	-0.111418000	4.928380000	0.000372000
1	-2.743459000	4.344147000	0.000302000
1	3.599088000	-3.731989000	0.000118000
1	-1.333757000	-4.104456000	0.000548000
1	-4.142063000	1.774640000	0.000191000
1	4.374846000	-1.111400000	0.000274000
7	-1.853804000	-0.749307000	-0.000947000
7	1.691790000	0.919177000	-0.000421000
7	0.993254000	-1.625846000	-0.000362000
7	-0.764682000	1.665709000	-0.000296000
6	-3.459130000	-2.307381000	0.000687000
6	2.804729000	2.837982000	0.000496000
6	-4.073777000	-1.068242000	0.000527000
6	3.735129000	1.786454000	0.000427000
6	-2.033155000	-2.108068000	-0.000041000
6	1.501861000	2.266573000	-0.000071000
6	-3.052817000	-0.059901000	-0.000278000
6	3.006622000	0.557085000	-0.000065000
6	-1.008157000	-3.067139000	0.000070000
6	-3.145779000	1.340723000	-0.000141000
6	3.328229000	-0.820896000	0.000014000
6	0.386446000	-2.842913000	-0.000089000
6	0.128373000	2.678923000	-0.000124000
6	-2.018743000	2.196109000	-0.000186000
6	2.356290000	-1.840165000	-0.000099000
6	1.397662000	-3.895373000	0.000139000
6	-0.562961000	3.943016000	0.000162000
6	-1.917532000	3.641093000	0.000126000
6	2.617001000	-3.271961000	0.000019000
1	-0.972403000	-0.239221000	-0.000561000
1	0.961117000	0.204034000	-0.000561000

$E = -950.9987519$ a. u.
$H = -950.706041$ a. u.
$G = -950.765899$ a. u.

SUPPORTING INFORMATION MATERIAL

Cor_parallel – Unsubstituted *cisoid* corrole with parallel anchoring of the B-O-B bridge

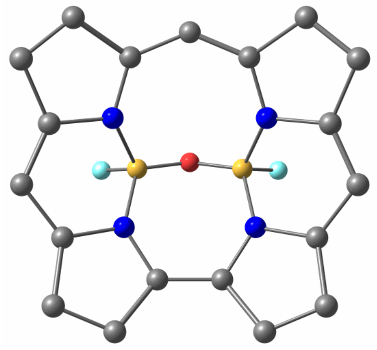

1	-4.196388000	-0.960696000	2.392807000
1	4.382039000	-0.889892000	-1.252421000
1	-2.434481000	-1.390049000	4.382285000
1	3.240917000	-1.306414000	-3.653226000
1	-4.196388000	-0.960696000	-2.392807000
1	4.382039000	-0.889892000	1.252421000
1	3.240917000	-1.306414000	3.653226000
1	-2.434481000	-1.390049000	-4.382285000
1	-4.068522000	-0.236639000	0.000000000
1	0.440523000	-1.062669000	4.483135000
1	0.440523000	-1.062669000	-4.483135000
7	-1.135238000	0.061694000	1.693901000
7	1.282414000	0.223642000	-1.464654000
7	-1.135238000	0.061694000	-1.693901000
7	1.282414000	0.223642000	1.464654000
6	-3.150564000	-0.679587000	2.415508000
6	3.392353000	-0.584847000	-1.565036000
6	-2.247426000	-0.910839000	3.429392000
6	2.804092000	-0.803906000	-2.799560000
6	-2.437486000	-0.112585000	1.292007000
6	2.408913000	0.016846000	-0.711802000
6	-0.966516000	-0.469718000	2.962502000
6	1.451586000	-0.349488000	-2.708019000
6	-2.996179000	-0.051105000	0.000000000
6	0.318724000	-0.612180000	3.503909000
6	0.318724000	-0.612180000	-3.503909000
6	-2.437486000	-0.112585000	-1.292007000
6	2.408913000	0.016846000	0.711802000
6	1.451586000	-0.349488000	2.708019000
6	-0.966516000	-0.469718000	-2.962502000
6	-3.150564000	-0.679587000	-2.415508000
6	3.392353000	-0.584847000	1.565036000
6	2.804092000	-0.803906000	2.799560000
6	-2.247426000	-0.910839000	-3.429392000
5	-0.025878000	1.085322000	1.249246000
5	-0.025878000	1.085322000	-1.249246000
8	-0.192256000	1.644411000	0.000000000
9	0.001814000	2.101952000	2.249115000
9	0.001814000	2.101952000	-2.249115000

E = – 1274.7632269 a. u.
H = – 1274.467812 a. u.
G = – 1274.530810 a. u.

Cor_perpendicular – Unsubstituted *cisoid* corrole with perpendicular anchoring of the B-O-B bridge

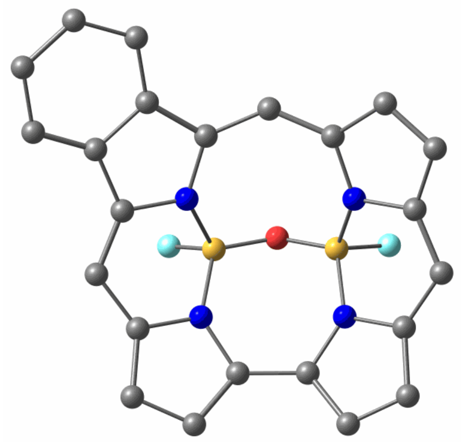

1	4.082315000	2.824442000	-1.332279000
1	-4.318390000	-1.850577000	-1.721293000
1	2.060437000	4.456611000	-0.621283000
1	-2.752147000	-3.963226000	-1.128785000
1	4.081370000	-2.825640000	-1.332448000
1	-4.315198000	1.853654000	-1.724116000
1	-2.747197000	3.964861000	-1.130811000
1	2.058882000	-4.457311000	-0.622246000
1	4.371034000	-0.000850000	-1.242319000
1	-0.284740000	4.190915000	-0.023680000
1	-0.286741000	-4.191033000	-0.024198000
7	1.482368000	1.252039000	0.034255000
7	-1.713880000	-1.140570000	0.184707000
7	1.481506000	-1.252599000	0.033727000
7	-1.716045000	1.142538000	0.188697000
6	3.149724000	2.554533000	-0.853229000
6	-3.435856000	-1.848627000	-1.095069000
6	2.125117000	3.385041000	-0.475722000
6	-2.635175000	-2.948489000	-0.768134000
6	2.735745000	1.216547000	-0.549366000
6	-2.823360000	-0.717344000	-0.504640000
6	1.037383000	2.574134000	0.027411000
6	-1.511527000	-2.467969000	-0.012865000
6	3.381612000	-0.000600000	-0.798586000
6	-0.254062000	3.111619000	0.108804000
6	-0.255466000	-3.111660000	0.107366000
6	2.735093000	-1.217454000	-0.549651000
6	-2.824134000	0.719878000	-0.503106000
6	-1.510873000	2.469192000	-0.010756000
6	1.036215000	-2.574634000	0.026432000
6	3.148751000	-2.555507000	-0.853579000
6	-3.433893000	1.851327000	-1.096173000
6	-2.632395000	2.950333000	-0.768907000
6	2.123811000	-3.385786000	-0.476471000
5	-1.271958000	-0.000376000	1.188389000
5	1.206150000	-0.000321000	0.999601000
8	0.009931000	0.000608000	1.676926000
9	2.281962000	-0.000921000	1.936386000
9	-2.212846000	-0.003557000	2.256486000

E = – 1274.7315636 a. u.
H = – 1274.436336 a. u.
G = – 1274.500028 a. u.

Cor_2a – Corrole akamptisomer c_1

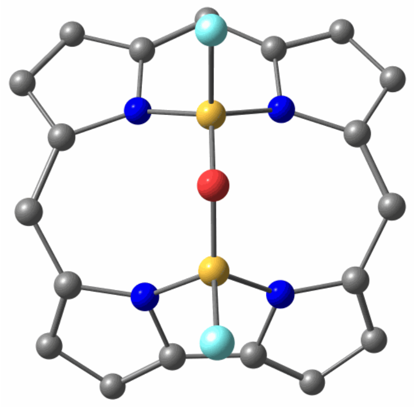

1	-0.124465000	-4.694685000	-0.679771000
1	2.324070000	4.273798000	-1.123032000
1	2.457083000	-4.573033000	-1.440930000
1	-0.301790000	4.865097000	-1.219936000
1	4.277952000	2.714809000	-1.339280000
1	5.400225000	0.319269000	-1.831115000
1	-1.797266000	-3.103798000	0.256394000
1	4.350717000	-2.378524000	-1.396381000
1	-2.644630000	3.206245000	-0.612076000
7	1.354380000	-1.854490000	0.111441000
7	0.390393000	2.001839000	0.287673000
7	-1.312286000	0.254475000	0.402426000
7	2.676730000	0.186693000	0.049689000
6	0.575637000	-3.888119000	-0.499999000
6	1.511271000	3.699061000	-0.699594000
6	1.894529000	-3.822453000	-0.899979000
6	0.157609000	4.002174000	-0.755048000
6	0.219863000	-2.621765000	0.095567000
6	1.647238000	2.415586000	-0.084412000
6	2.378498000	-2.525599000	-0.540393000
6	-0.542041000	2.899463000	-0.183449000
6	-1.125913000	-2.252002000	0.332996000
6	3.571144000	-1.855650000	-0.852515000
6	-1.899847000	2.506624000	-0.248861000
6	-1.803521000	-1.027775000	0.331752000
6	2.755532000	1.528073000	-0.206141000
6	3.681977000	-0.470220000	-0.633862000
6	-2.253580000	1.179689000	-0.023421000
6	-3.219408000	-0.907677000	-0.025508000
6	3.952341000	1.753384000	-0.965579000
6	4.527443000	0.520563000	-1.223029000
6	-3.488371000	0.467944000	-0.260913000
5	1.815120000	-0.680943000	1.050464000
5	-0.131953000	0.873888000	1.258519000
8	0.803072000	-0.025058000	1.719253000
9	2.737871000	-1.249270000	1.976260000
9	-0.763871000	1.539147000	2.348175000
6	-5.761917000	-0.066129000	-0.834703000
6	-5.498035000	-1.433343000	-0.603012000
6	-4.230296000	-1.860646000	-0.213030000
6	-4.760309000	0.887876000	-0.675059000
1	-4.036917000	-2.917079000	-0.048305000
1	-6.295052000	-2.161035000	-0.732290000
1	-6.758254000	0.242119000	-1.141171000
1	-4.961665000	1.939708000	-0.860401000

SUPPORTING INFORMATION MATERIAL

E = – 1428.4296969 a. u.
H = – 1428.084516 a. u.
G = – 1428.153640 a. u.

Cor_2a – Corrole akamptisomer (t_1)

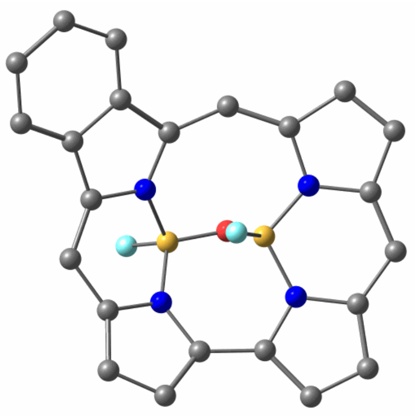

1	-0.113508000	4.972025000	0.096744000
1	-2.375303000	-4.167486000	-1.201140000
1	-2.745350000	4.926345000	-0.398705000
1	0.234614000	-4.824514000	-1.193086000
1	-4.692202000	-2.625822000	-0.697242000
1	-5.833750000	-0.212466000	-0.950371000
1	1.754036000	3.084935000	0.336955000
1	-4.633125000	2.558935000	-0.700799000
1	2.576398000	-3.242682000	-0.438445000
7	-1.411659000	1.975791000	0.108341000
7	-0.462640000	-2.034781000	0.431600000
7	1.326335000	-0.297395000	0.641430000
7	-2.677322000	-0.126132000	-0.071073000
6	-0.746560000	4.094256000	0.052684000
6	-1.574426000	-3.639721000	-0.699579000
6	-2.108883000	4.071617000	-0.207109000
6	-0.224843000	-3.976223000	-0.701806000
6	-0.290461000	2.731580000	0.246843000
6	-1.719610000	-2.379476000	-0.040238000
6	-2.535046000	2.692453000	-0.186838000
6	0.472583000	-2.919169000	-0.039375000
6	1.049928000	2.257919000	0.404308000
6	-3.743067000	1.999048000	-0.434435000
6	1.845492000	-2.540250000	-0.051433000
6	1.730545000	1.007473000	0.427970000
6	-2.842521000	-1.474561000	-0.159132000
6	-3.804809000	0.578019000	-0.390030000
6	2.239446000	-1.220525000	0.192374000
6	3.135751000	0.892775000	0.001063000
6	-4.211206000	-1.667893000	-0.547464000
6	-4.805227000	-0.412925000	-0.677738000
6	3.442388000	-0.489873000	-0.155017000
5	-1.328720000	0.495812000	0.161494000
5	0.051811000	-0.865672000	1.369637000
8	-0.874787000	0.144326000	1.464479000
9	-0.537911000	0.130292000	-0.997850000
9	0.468517000	-1.436955000	2.592347000
6	5.660971000	0.071383000	-0.896191000
6	5.363727000	1.440830000	-0.742606000
6	4.104111000	1.856014000	-0.311142000
6	4.700235000	-0.898272000	-0.614675000
1	3.884267000	2.915933000	-0.218528000

1	6.125268000	2.182628000	-0.969492000
1	6.647778000	-0.228252000	-1.239954000
1	4.924149000	-1.954173000	-0.742589000

E = – 1428.4065289 a. u.
H = – 1428.061361 a. u.
G = – 1428.130076 a. u.

Cor_2a_TS – Corrole transition state (*TS*)

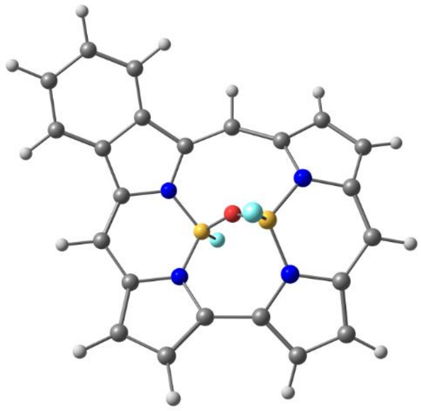

1	-4.497302000	-2.770049000	0.523311000
1	-5.825097000	-0.437289000	0.449118000
1	1.628964000	3.084223000	0.274029000
1	-4.798898000	2.334463000	0.103829000
1	2.805594000	-3.296555000	0.013457000
7	-1.503869000	2.006028000	-0.524336000
7	-0.320582000	-2.060009000	0.409024000
7	1.466286000	-0.291433000	0.735007000
7	-2.688026000	-0.240171000	-0.496620000
6	-0.933806000	3.913142000	0.480233000
6	-1.365948000	-3.799829000	-0.508420000
6	-2.316171000	3.892788000	0.375516000
6	-0.003939000	-4.119314000	-0.388082000
6	-0.402046000	2.643245000	0.018861000
6	-1.557274000	-2.461604000	-0.059084000
6	-2.682537000	2.617327000	-0.198951000
6	0.657346000	-2.977307000	0.159321000
6	0.984971000	2.202329000	0.217329000
6	-3.867230000	1.843081000	-0.160198000
6	2.039346000	-2.566351000	0.253527000
6	1.785597000	1.010328000	0.377043000
6	-2.739716000	-1.573234000	-0.157930000
6	-3.863578000	0.411663000	-0.239967000
6	2.418623000	-1.227207000	0.441226000
6	3.231996000	0.923754000	0.101980000
6	-4.104913000	-1.817529000	0.191505000
6	-4.796239000	-0.601923000	0.154673000
6	3.617760000	-0.457508000	0.119758000
5	-0.002351000	-0.672781000	0.945474000
5	-1.418022000	0.519107000	-0.874641000
8	-0.508301000	0.198454000	0.059645000
9	-0.380253000	-0.562694000	2.312920000
9	-1.102570000	0.307830000	-2.244529000
1	-2.132481000	-4.420338000	-0.954638000
1	0.474084000	-5.030882000	-0.723897000
6	4.176368000	1.900351000	-0.235811000
6	5.486403000	1.514140000	-0.522599000
6	5.860025000	0.156511000	-0.505204000

6	4.926528000	-0.833900000	-0.196071000
1	6.227012000	2.269877000	-0.771627000
1	6.884530000	-0.121270000	-0.739408000
1	5.212304000	-1.882633000	-0.195335000
1	3.892866000	2.948218000	-0.279498000
1	-3.004866000	4.643489000	0.742799000
1	-0.339104000	4.694641000	0.937564000

E = – 1428.3359062 a. u.
H = – 1427.992759 a. u.
G = – 1428.060884 a. u.
f = 215.807 *i*

Cor_2a – Corrole akamptisomer (t_2)

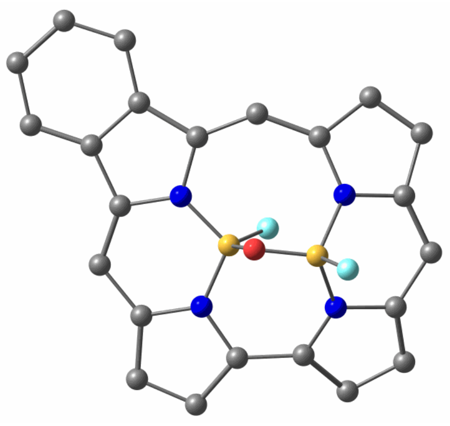

1	0.023510000	-4.545248000	-0.991531000
1	-2.299581000	4.604702000	-0.194037000
1	-2.635015000	-4.483255000	-1.361401000
1	0.318267000	5.195060000	-0.291566000
1	-4.315028000	2.777372000	-1.037466000
1	-5.543577000	0.405828000	-1.356895000
1	1.767313000	-3.068363000	-0.055132000
1	-4.533020000	-2.296335000	-0.954661000
1	2.748862000	3.370191000	-0.215771000
7	-1.428079000	-1.899643000	0.314119000
7	-0.329755000	2.007917000	0.091570000
7	1.434221000	0.279530000	0.100438000
7	-2.700442000	0.246202000	0.323304000
6	-0.666493000	-3.792398000	-0.630378000
6	-1.479206000	3.901680000	-0.128581000
6	-2.032218000	-3.762411000	-0.823375000
6	-0.115721000	4.209751000	-0.177470000
6	-0.256896000	-2.563405000	0.023176000
6	-1.608031000	2.488191000	0.046968000
6	-2.503798000	-2.522477000	-0.270843000
6	0.612496000	2.981833000	-0.051683000
6	1.134659000	-2.194740000	0.095188000
6	-3.700298000	-1.803804000	-0.462360000
6	1.992188000	2.603082000	-0.093975000
6	1.896483000	-1.004762000	0.108309000
6	-2.743684000	1.590237000	0.016797000
6	-3.764013000	-0.403392000	-0.258777000
6	2.381071000	1.257084000	-0.016877000
6	3.352615000	-0.856592000	0.009944000
6	-3.993676000	1.820643000	-0.646676000
6	-4.624123000	0.596026000	-0.817717000
6	3.650678000	0.543801000	-0.064944000
5	-1.700805000	-0.634914000	1.190381000

5	-0.033782000	0.534710000	0.146222000
8	-0.505112000	0.018744000	1.385726000
9	-2.383956000	-1.030492000	2.362422000
9	-0.531953000	-0.003217000	-1.101614000
6	5.999862000	0.032611000	-0.194419000
6	5.708938000	-1.344496000	-0.121694000
6	4.392004000	-1.793208000	-0.019021000
6	4.976487000	0.979854000	-0.166342000
1	5.203700000	2.041339000	-0.222030000
1	7.034075000	0.358130000	-0.271957000
1	6.522598000	-2.064943000	-0.143386000
1	4.177356000	-2.856733000	0.042233000

E = – 1428.4081236 a. u.
H = – 1428.062889 a. u.
G = – 1428.131438 a. u.

Por_1a – Porphyrin akamptisomer (c_1)

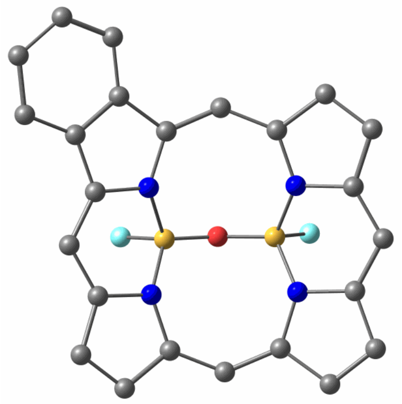

9	2.560873000	-1.283145000	1.899108000
5	-0.205873000	0.941651000	1.007852000
5	1.773544000	-0.708544000	0.874143000
8	0.812450000	0.117300000	1.395776000
9	-0.834648000	1.538851000	2.124271000
6	0.999133000	4.227639000	-0.408979000
6	1.351467000	2.882398000	-0.009287000
6	2.688716000	2.473591000	0.030768000
6	3.330987000	1.236141000	-0.145324000
6	4.675377000	1.164966000	-0.671334000
6	4.923933000	-0.138260000	-1.020094000
6	3.752867000	-0.885996000	-0.676160000
6	3.465173000	-2.245148000	-0.854706000
6	2.206993000	-2.752223000	-0.537795000
6	1.662869000	-4.066333000	-0.721321000
6	0.368852000	-4.022771000	-0.275917000
6	0.088416000	-2.666792000	0.152538000
6	-1.231919000	-2.253311000	0.410703000
6	-1.904573000	-1.035824000	0.304048000
6	-3.344541000	-0.977214000	0.028611000
6	-3.648684000	0.356987000	-0.322484000
6	-2.412871000	1.100307000	-0.208887000
6	-2.152242000	2.441056000	-0.460722000
6	-0.849291000	2.947760000	-0.362984000
6	-0.348660000	4.258589000	-0.653208000
7	0.189623000	2.143232000	0.045729000
7	2.817170000	-0.035414000	-0.125023000
7	1.236331000	-1.936974000	0.017631000
7	-1.414041000	0.242884000	0.204883000

1	1.715443000	5.024345000	-0.561024000
1	5.316144000	2.022995000	-0.826393000
1	5.809496000	-0.544043000	-1.490092000
1	2.188970000	-4.904756000	-1.157400000
1	-0.364837000	-4.817678000	-0.300820000
1	-0.941029000	5.087275000	-1.016608000
1	3.380005000	3.310378000	-0.028702000
1	4.214601000	-2.898557000	-1.286283000
1	-1.909949000	-3.098531000	0.481880000
1	-2.956399000	3.094970000	-0.776431000
6	-4.944808000	0.730846000	-0.696344000
6	-5.936200000	-0.246529000	-0.696595000
1	-6.953191000	0.017123000	-0.970654000
6	-5.637445000	-1.578591000	-0.344874000
1	-6.430344000	-2.320409000	-0.349297000
6	-4.343888000	-1.957985000	0.006615000
1	-4.130165000	-2.989696000	0.268378000
1	-5.174278000	1.755375000	-0.973298000

E = – 1467.0503673 a. u.
H = – 1466.684921 a. u.
G = – 1466.756522 a. u.

Por_1a – Porphyrin akamptisomer (t_1)

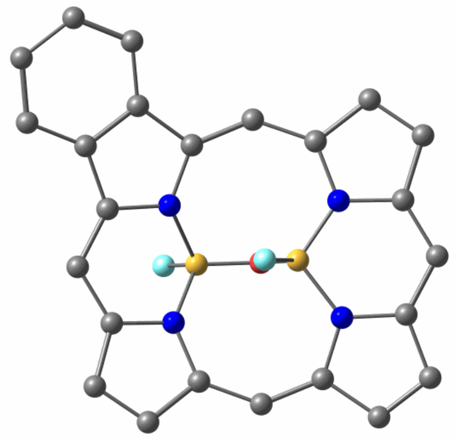

9	0.772541000	-0.047893000	-1.253377000
5	-0.149079000	0.882439000	1.093341000
5	1.435112000	-0.470708000	-0.067383000
8	0.923880000	0.027696000	1.146464000
9	-0.602119000	1.314771000	2.350825000
6	0.927792000	4.196362000	-0.437196000
6	1.305515000	2.851709000	-0.037617000
6	2.651978000	2.441515000	-0.097801000
6	3.371164000	1.229558000	-0.158524000
6	4.811402000	1.159865000	-0.284694000
6	5.171749000	-0.162251000	-0.367985000
6	3.958759000	-0.931984000	-0.299451000
6	3.738574000	-2.319962000	-0.292502000
6	2.450192000	-2.840077000	-0.135081000
6	1.972126000	-4.194037000	-0.013769000
6	0.616815000	-4.123770000	0.178733000
6	0.229893000	-2.724949000	0.182531000
6	-1.107836000	-2.290411000	0.304229000
6	-1.820624000	-1.079099000	0.272485000
6	-3.265340000	-1.056230000	-0.008722000
6	-3.651796000	0.297398000	-0.145437000
6	-2.454768000	1.084702000	0.072249000
6	-2.232465000	2.451932000	-0.083424000

6	-0.928343000	2.976860000	-0.076048000
6	-0.439009000	4.267450000	-0.477931000
7	0.135835000	2.173732000	0.226969000
7	2.912280000	-0.059664000	-0.185568000
7	1.370053000	-2.004516000	-0.022007000
7	-1.422259000	0.231379000	0.371805000
1	1.634038000	4.965309000	-0.721640000
1	5.466001000	2.021212000	-0.300686000
1	6.167996000	-0.573064000	-0.458688000
1	2.589830000	-5.080553000	-0.059699000
1	-0.071216000	-4.947857000	0.313520000
1	-1.052698000	5.101660000	-0.789411000
1	3.315389000	3.295035000	-0.219511000
1	4.582275000	-2.994637000	-0.378387000
1	-1.781392000	-3.142637000	0.335168000
1	-3.064010000	3.101703000	-0.329792000
6	-5.900351000	-0.373928000	-0.627314000
6	-4.967679000	0.647855000	-0.464026000
6	-5.520864000	-1.723699000	-0.493207000
1	-5.257402000	1.688293000	-0.576718000
1	-6.265962000	-2.502179000	-0.626921000
6	-4.204991000	-2.076139000	-0.197337000
1	-3.928872000	-3.122720000	-0.113451000
1	-6.931703000	-0.130220000	-0.863661000

E = – 1467.0618894 a. u.
H = – 1466.696063 a. u.
G = – 1466.767053 a. u.

Por_1a_TS – Porphyrin transition state (*TS*)

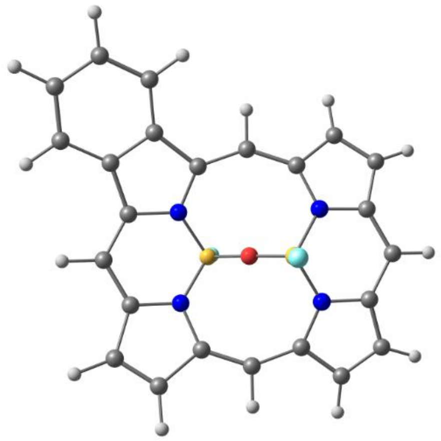

1	5.268264000	2.280245000	0.279848000
1	6.193545000	-0.235532000	0.307816000
1	3.093144000	3.394373000	-0.167709000
1	-3.324764000	3.113576000	0.008982000
1	4.756944000	-2.808551000	0.121345000
7	-0.072950000	2.270893000	0.273044000
7	1.502602000	-1.992689000	-0.210959000
7	2.966042000	0.015488000	-0.320233000
7	-1.557170000	0.267295000	0.375386000
6	0.678797000	4.305309000	-0.314464000
6	0.762714000	-4.007697000	0.442317000
6	-0.691957000	4.376724000	-0.249402000
6	2.133019000	-4.081045000	0.374494000
6	1.092990000	2.938150000	-0.030494000
6	0.349105000	-2.648128000	0.117923000
6	-1.164782000	3.058755000	0.106264000
6	2.605584000	-2.772438000	-0.016573000

6	2.450280000	2.516755000	-0.108114000
6	-2.457866000	2.478543000	0.149304000
6	3.886076000	-2.185006000	-0.046647000
6	3.265981000	1.339665000	-0.129275000
6	-1.840193000	-1.064923000	0.194372000
6	-2.633721000	1.086132000	0.217202000
6	4.054118000	-0.781059000	-0.134596000
6	4.701850000	1.376688000	0.095478000
6	-3.301908000	-1.111148000	0.001167000
6	-3.788601000	0.225313000	0.005663000
6	5.180258000	0.086384000	0.109412000
5	1.588393000	-0.542913000	-0.710230000
5	-0.169239000	0.822697000	0.768099000
8	0.704405000	0.130278000	0.034980000
9	1.435316000	-0.504642000	-2.115641000
9	-0.041999000	0.783080000	2.176297000
6	-5.142896000	0.500581000	-0.199451000
6	-6.014576000	-0.570000000	-0.395134000
6	-5.538252000	-1.893988000	-0.401852000
6	-4.184323000	-2.174010000	-0.215887000
1	-7.073103000	-0.382123000	-0.547147000
1	-6.236261000	-2.710606000	-0.559866000
1	-3.832386000	-3.200706000	-0.242674000
1	-5.509649000	1.522716000	-0.202190000
1	0.085041000	-4.796106000	0.742981000
1	2.757322000	-4.931712000	0.612124000
6	-1.019914000	-2.224116000	0.181251000
1	-1.650782000	-3.109215000	0.243736000
1	-1.315707000	5.234364000	-0.461673000
1	1.357171000	5.102639000	-0.589067000

E = – 1467.0201399 a. u.
H = – 1466.655923 a. u.
G = – 1466.726547 a. u.
f = 183.802 i

Por_1a – Porphyrin akamptisomer (t_2)

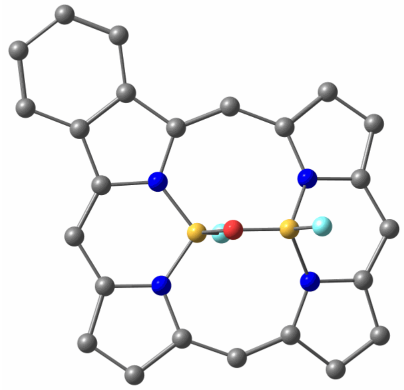

9	-2.199468000	-1.018712000	2.218118000
5	0.031181000	0.687644000	-0.083140000
5	-1.665815000	-0.609984000	0.983579000
8	-0.570723000	0.217168000	1.095964000
9	-0.525379000	0.240536000	-1.311163000
6	-0.751031000	4.363265000	0.009749000
6	-1.165479000	2.974562000	0.019747000
6	-2.509324000	2.570515000	0.055762000
6	-3.235982000	1.357015000	0.008835000
6	-4.629257000	1.347793000	-0.394738000

6	-5.026184000	0.043666000	-0.540660000
6	-3.891719000	-0.769440000	-0.204885000
6	-3.692172000	-2.154381000	-0.333953000
6	-2.414216000	-2.708767000	-0.218200000
6	-1.919013000	-4.010371000	-0.577719000
6	-0.557409000	-3.960653000	-0.445329000
6	-0.187796000	-2.619861000	-0.020613000
6	1.176448000	-2.236271000	0.036328000
6	1.927347000	-1.055600000	0.016344000
6	3.394379000	-1.023492000	0.011571000
6	3.791139000	0.333399000	-0.076509000
6	2.571232000	1.119384000	-0.122954000
6	2.370657000	2.498069000	-0.169251000
6	1.073626000	3.039534000	-0.126689000
6	0.616581000	4.404264000	-0.077156000
7	-0.017895000	2.224945000	-0.078280000
7	-2.854704000	0.048407000	0.164816000
7	-1.349748000	-1.923551000	0.158455000
7	1.516546000	0.250025000	-0.073656000
1	-1.431156000	5.202710000	0.069153000
1	-5.206652000	2.237798000	-0.607910000
1	-5.983317000	-0.325267000	-0.883404000
1	-2.523044000	-4.836045000	-0.928627000
1	0.154306000	-4.741912000	-0.677623000
1	1.254475000	5.277242000	-0.096808000
1	-3.177975000	3.427822000	0.019263000
1	-4.520187000	-2.783145000	-0.641027000
1	1.818543000	-3.111903000	-0.014544000
1	3.225286000	3.162848000	-0.203936000
6	4.358526000	-2.034819000	0.083666000
6	5.704872000	-1.673803000	0.062445000
1	6.466892000	-2.445351000	0.117404000
6	6.094792000	-0.323281000	-0.025648000
1	7.151167000	-0.072641000	-0.038383000
6	5.142562000	0.691657000	-0.094372000
1	5.444630000	1.732681000	-0.159777000
1	4.074759000	-3.079989000	0.159734000

E = – 1467.0624275 a. u.
H = – 1466.696604 a. u.
G = – 1466.767566 a. u.

Cor_2b – B_2OY_2-corrole, Y_1=Y_2=OH (c_1)

1	0.143192000	4.688853000	-0.688071000
1	-2.328744000	-4.281987000	-1.114959000
1	-2.427350000	4.565006000	-1.485189000
1	0.297218000	-4.871778000	-1.223102000

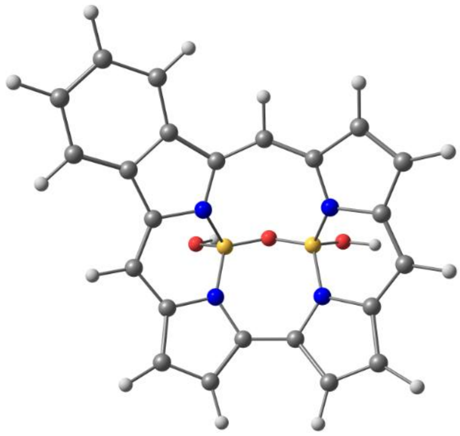

1	-4.268197000	-2.722769000	-1.351598000
1	-5.380138000	-0.328601000	-1.874134000
1	1.801610000	3.100335000	0.268681000
1	-4.328396000	2.373242000	-1.444632000
1	2.641604000	-3.207785000	-0.627168000
7	-1.349038000	1.850833000	0.099567000
7	-0.388686000	-1.999192000	0.272583000
7	1.310791000	-0.256069000	0.385022000
7	-2.671779000	-0.185944000	0.033572000
6	-0.560735000	3.884604000	-0.512049000
6	-1.513567000	-3.705221000	-0.698653000
6	-1.873416000	3.818354000	-0.929773000
6	-0.161062000	-4.007014000	-0.760269000
6	-0.214320000	2.618124000	0.092195000
6	-1.644931000	-2.415997000	-0.088730000
6	-2.360616000	2.520362000	-0.571701000
6	0.539977000	-2.898636000	-0.197224000
6	1.129308000	2.248413000	0.337791000
6	-3.556232000	1.852319000	-0.888170000
6	1.896503000	-2.507163000	-0.266376000
6	1.806452000	1.022282000	0.327884000
6	-2.753307000	-1.528606000	-0.213260000
6	-3.668655000	0.464623000	-0.666391000
6	2.250461000	-1.179335000	-0.041134000
6	3.223179000	0.903817000	-0.027840000
6	-3.945156000	-1.759138000	-0.981003000
6	-4.515471000	-0.527725000	-1.253621000
6	3.488713000	-0.471147000	-0.272120000
5	-1.832718000	0.691089000	1.082542000
5	0.140690000	-0.882405000	1.276134000
8	-0.805193000	0.023818000	1.725135000
6	5.765456000	0.060767000	-0.836318000
6	5.504578000	1.427188000	-0.597108000
6	4.236654000	1.855286000	-0.207421000
6	4.760571000	-0.891642000	-0.685416000
1	4.045415000	2.911270000	-0.036812000
1	6.303824000	2.153641000	-0.720162000
1	6.761856000	-0.248283000	-1.142041000
1	4.959543000	-1.942843000	-0.877149000
8	-2.726789000	1.235891000	2.090146000
1	-3.450248000	1.741595000	1.706672000
8	0.786850000	-1.549555000	2.399033000
1	0.334691000	-1.229042000	3.186811000

E = – 1380.3516321 a. u.

H = – 1379.982077 a. u.

G = – 1380.052897 a. u.

Cor_2b – B_2OY_2-corrole, Y_1=Y_2=OH (t_1)

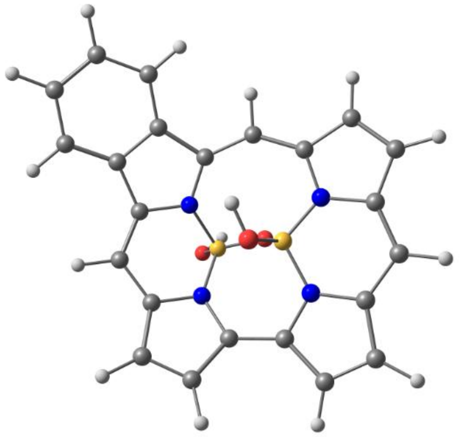

1	-0.150061000	4.988870000	0.237129000
1	-2.384104000	-4.201704000	-1.151719000
1	-2.786736000	4.934634000	-0.228846000
1	0.227583000	-4.854175000	-1.141898000
1	-4.752700000	-2.628853000	-0.463252000
1	-5.891273000	-0.213173000	-0.712519000
1	1.765413000	3.093165000	0.332406000
1	-4.658905000	2.558159000	-0.593828000
1	2.574306000	-3.264543000	-0.395410000
7	-1.385575000	1.978436000	-0.034451000
7	-0.469074000	-2.035130000	0.426744000
7	1.321241000	-0.294043000	0.598616000
7	-2.662280000	-0.133392000	-0.165684000
6	-0.764795000	4.105720000	0.112177000
6	-1.582236000	-3.663631000	-0.662703000
6	-2.130486000	4.078781000	-0.131489000
6	-0.231228000	-3.997103000	-0.665333000
6	-0.284073000	2.738627000	0.191361000
6	-1.725304000	-2.390014000	-0.028605000
6	-2.531310000	2.693167000	-0.219960000
6	0.466685000	-2.925400000	-0.024882000
6	1.059876000	2.264960000	0.366705000
6	-3.748028000	1.995996000	-0.416611000
6	1.841822000	-2.548914000	-0.035958000
6	1.743005000	1.009190000	0.403511000
6	-2.848001000	-1.478472000	-0.157341000
6	-3.807819000	0.573255000	-0.384668000
6	2.241521000	-1.226773000	0.188402000
6	3.158948000	0.882174000	0.012462000
6	-4.249267000	-1.671995000	-0.412579000
6	-4.841578000	-0.415824000	-0.540393000
6	3.458988000	-0.505556000	-0.129668000
5	-1.293677000	0.486203000	-0.024679000
5	0.018981000	-0.847505000	1.347290000
8	-0.878088000	0.195852000	1.334721000
6	5.700886000	0.039746000	-0.815198000
6	5.409381000	1.411967000	-0.676277000
6	4.141424000	1.837323000	-0.279524000
6	4.726894000	-0.922540000	-0.553864000
1	3.925255000	2.899049000	-0.197907000
1	6.181425000	2.147578000	-0.887403000
1	6.694172000	-0.268123000	-1.132109000
1	4.947643000	-1.980387000	-0.670808000
8	-0.590358000	0.046636000	-1.238998000
1	0.244215000	0.497722000	-1.385442000
8	0.385315000	-1.384546000	2.639176000
1	0.240273000	-0.684447000	3.285369000

$E = -1380.3255591$ a. u.

$H = -1379.956215$ a. u.

$G = -1380.027299$ a. u.

SUPPORTING INFORMATION MATERIAL

Cor_2c – B_2OY_2-corrole, Y_1=NH_2; Y_2=CO_2H (c_1)

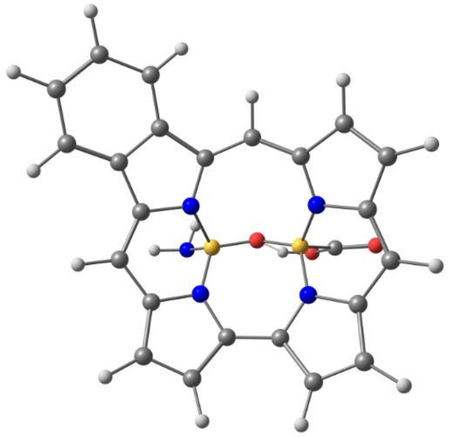

1	-0.074093000	-4.556476000	-1.130313000
1	1.719361000	4.564060000	-1.202343000
1	2.405832000	-4.194586000	-2.120215000
1	-0.941984000	4.960488000	-1.113165000
1	3.737814000	3.181884000	-1.638648000
1	4.973942000	0.915547000	-2.395609000
1	-1.750421000	-3.159317000	0.078774000
1	4.155356000	-1.877157000	-2.070531000
1	-3.120483000	3.096931000	-0.446820000
7	1.280476000	-1.676469000	-0.277901000
7	0.048772000	2.089958000	0.201099000
7	-1.500649000	0.219121000	0.333866000
7	2.435258000	0.456681000	-0.317574000
6	0.584647000	-3.714455000	-0.957548000
6	0.980099000	3.912137000	-0.756703000
6	1.849247000	-3.524070000	-1.478260000
6	-0.393758000	4.115559000	-0.716683000
6	0.204172000	-2.522493000	-0.240333000
6	1.246151000	2.614856000	-0.220207000
6	2.278147000	-2.224440000	-1.073777000
6	-0.973325000	2.936309000	-0.166196000
6	-1.137877000	-2.263069000	0.140162000
6	3.384776000	-1.446963000	-1.440771000
6	-2.303036000	2.441438000	-0.166936000
6	-1.899770000	-1.094150000	0.243422000
6	2.401531000	1.815575000	-0.477556000
6	3.421090000	-0.077214000	-1.131439000
6	-2.536528000	1.079480000	0.004307000
6	-3.345849000	-1.073148000	-0.005971000
6	3.511845000	2.176333000	-1.310741000
6	4.143929000	1.009936000	-1.707509000
6	-3.731234000	0.284231000	-0.174214000
5	1.723269000	-0.532033000	0.674577000
5	-0.316261000	0.909853000	1.182007000
8	0.715795000	0.011929000	1.475199000
6	-5.995313000	-0.402188000	-0.606529000
6	-5.616168000	-1.751005000	-0.441314000
6	-4.294915000	-2.093050000	-0.155997000
6	-5.056272000	0.619661000	-0.484555000
1	-4.013884000	-3.136513000	-0.043395000
1	-6.364636000	-2.532877000	-0.540649000
1	-7.030610000	-0.160872000	-0.833341000
1	-5.345653000	1.658201000	-0.622566000
6	2.878939000	-1.104868000	1.764600000
8	3.831995000	-1.841702000	1.567988000
8	2.670872000	-0.627288000	3.032197000
7	-0.824221000	1.486885000	2.489174000
1	-0.990412000	0.748150000	3.166762000

1	-1.663799000	2.053014000	2.430012000
1	1.838712000	-0.112706000	2.959903000

E = – 1473.7908152 a. u.
H = – 1473.396226 a. u.
G = – 1473.471880 a. u.

Cor_2c – B_2OY_2-corrole, Y1=NH_2; Y2=CO_2H (t_1)

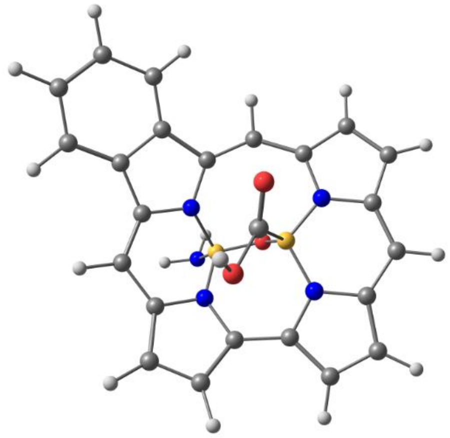

1	-0.121530000	4.980326000	0.586328000
1	-2.453952000	-4.246754000	-0.847651000
1	-2.755251000	4.956881000	0.092148000
1	0.147782000	-4.927866000	-0.840961000
1	-4.800898000	-2.550102000	-0.085120000
1	-5.896210000	-0.116301000	-0.380911000
1	1.800326000	3.090572000	0.563973000
1	-4.626868000	2.615061000	-0.381452000
1	2.539907000	-3.300333000	-0.220429000
7	-1.335633000	2.003496000	0.014512000
7	-0.488783000	-2.014427000	0.564708000
7	1.300512000	-0.292168000	0.682117000
7	-2.625772000	-0.105732000	-0.150996000
6	-0.730805000	4.110754000	0.372730000
6	-1.640583000	-3.691013000	-0.399189000
6	-2.092762000	4.100800000	0.114164000
6	-0.291091000	-4.038822000	-0.405509000
6	-0.243865000	2.746358000	0.338703000
6	-1.757999000	-2.379853000	0.152752000
6	-2.486219000	2.726518000	-0.093398000
6	0.430916000	-2.940252000	0.152993000
6	1.089734000	2.266478000	0.540176000
6	-3.709589000	2.044711000	-0.281369000
6	1.810537000	-2.567982000	0.110445000
6	1.753067000	1.004638000	0.527946000
6	-2.854918000	-1.445110000	-0.015034000
6	-3.778224000	0.621604000	-0.277041000
6	2.214342000	-1.237864000	0.284549000
6	3.168456000	0.859354000	0.155160000
6	-4.271262000	-1.608171000	-0.143866000
6	-4.840662000	-0.342276000	-0.296116000
6	3.447090000	-0.530872000	-0.008242000
5	-1.259943000	0.513207000	0.035340000
5	0.000697000	-0.818596000	1.470450000
8	-0.885844000	0.233500000	1.392037000
6	5.706231000	-0.012135000	-0.654270000
6	5.434723000	1.362422000	-0.497703000
6	4.169312000	1.802711000	-0.110732000
6	4.713380000	-0.962526000	-0.422176000
1	3.966021000	2.866070000	-0.021431000
1	6.220485000	2.088310000	-0.691051000
1	6.697900000	-0.330815000	-0.965571000
1	4.917650000	-2.021612000	-0.557455000
6	-0.404234000	0.177946000	-1.421935000

8	0.178799000	1.026882000	-2.085639000
8	-0.539737000	-1.065782000	-1.989211000
1	-0.079657000	-1.003735000	-2.844737000
7	0.306876000	-1.305100000	2.861699000
1	0.560591000	-0.531578000	3.469891000
1	1.040333000	-2.004967000	2.915482000

E = – 1473.7525908 a. u.
H = – 1473.359109 a. u.
G = – 1473.434397 a. u.

Cor_2d – B_2OY_2-corrole, Y_1=$N(^tBu)_2$; Y_2=CO_2H (c_1)

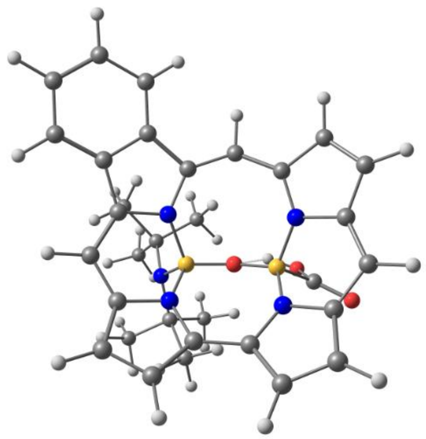

1	-0.649929000	-4.826004000	-1.890515000
1	-1.778475000	1.906312000	4.285608000
1	-3.222766000	-4.891941000	-1.095987000
1	0.897000000	2.161541000	4.499118000
1	-3.858970000	0.862852000	3.655896000
1	-5.371732000	-1.089170000	2.590796000
1	1.251343000	-3.276952000	-1.585948000
1	-4.791433000	-2.944199000	0.410505000
1	2.978550000	0.985426000	2.878999000
7	-1.717620000	-1.971082000	-0.641019000
7	-0.130556000	1.036812000	1.550771000
7	1.300836000	-0.429483000	0.283346000
7	-2.698923000	-0.306071000	0.801112000
6	-1.233141000	-4.007399000	-1.487599000
6	-1.041468000	1.714265000	3.518000000
6	-2.547340000	-4.046622000	-1.068754000
6	0.333989000	1.851685000	3.628331000
6	-0.692062000	-2.708176000	-1.164176000
6	-1.322731000	1.147858000	2.238781000
6	-2.842828000	-2.771534000	-0.495304000
6	0.893826000	1.346480000	2.421443000
6	0.699215000	-2.458566000	-1.131113000
6	-3.922475000	-2.311545000	0.270052000
6	2.171730000	0.804548000	2.177518000
6	1.529165000	-1.628816000	-0.365088000
6	-2.550116000	0.531188000	1.871589000
6	-3.802705000	-1.117068000	1.000341000
6	2.305934000	-0.181508000	1.211098000
6	2.854271000	-2.087745000	0.060576000
6	-3.695252000	0.350834000	2.717813000
6	-4.471351000	-0.657087000	2.174206000
6	3.322857000	-1.190331000	1.053744000
5	-1.958240000	-0.430555000	-0.558835000
5	0.275635000	0.774362000	0.009074000
8	-0.821118000	0.356798000	-0.766328000
6	5.319192000	-2.501436000	1.319257000
6	4.855596000	-3.399953000	0.333383000
6	3.623074000	-3.208967000	-0.286012000

6	4.553252000	-1.401096000	1.692108000
1	3.273746000	-3.917470000	-1.031661000
1	5.468814000	-4.253969000	0.057983000
1	6.282630000	-2.675190000	1.791397000
1	4.903849000	-0.713982000	2.457628000
6	-3.003531000	-0.025658000	-1.818571000
8	-4.215460000	-0.132332000	-1.893502000
8	-2.325852000	0.470319000	-2.907807000
7	0.979821000	2.034908000	-0.636626000
1	-1.404276000	0.569117000	-2.598337000
6	1.950283000	1.796443000	-1.783844000
6	0.139951000	3.295633000	-0.735044000
6	-0.592673000	3.497181000	-2.097302000
1	-1.374661000	2.743487000	-2.214861000
1	-1.078222000	4.481582000	-2.106919000
1	0.044095000	3.449435000	-2.977200000
6	0.988434000	4.548809000	-0.355825000
1	1.721354000	4.849094000	-1.099589000
1	0.329137000	5.411123000	-0.192667000
1	1.517069000	4.338025000	0.579523000
6	-1.028792000	3.359798000	0.277963000
1	-0.684721000	3.400317000	1.311023000
1	-1.581605000	4.285398000	0.084043000
1	-1.733271000	2.540434000	0.151247000
6	1.337144000	0.920631000	-2.912012000
1	0.967167000	-0.025189000	-2.515265000
1	0.508748000	1.425043000	-3.415293000
1	2.096532000	0.689624000	-3.671285000
6	3.252795000	1.091605000	-1.311863000
1	3.585805000	1.505964000	-0.358568000
1	3.151772000	0.016036000	-1.223226000
1	4.044550000	1.255850000	-2.051790000
6	2.536847000	3.075206000	-2.456271000
1	3.147957000	2.745290000	-3.303455000
1	1.812081000	3.780216000	-2.852322000
1	3.198895000	3.607056000	-1.766623000

E = – 1788.2951893 a. u.
H = – 1787.664131 a. u.
G = – 1787.758124 a. u.

Cor_2d – B_2OY_2-corrole, Y_1=N(^{t}Bu)$_2$; Y_2=CO_2H (t_1)

1	-0.216212000	-3.941984000	-3.502117000
1	-2.447484000	1.290536000	4.115639000
1	-2.870602000	-4.166852000	-3.222001000
1	0.144318000	1.855325000	4.600934000
1	-4.757497000	0.721562000	2.574632000
1	-5.929936000	-1.036351000	0.920136000

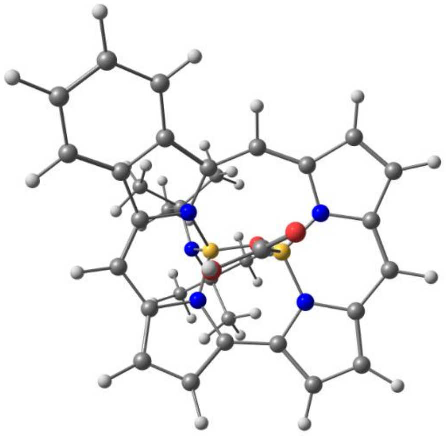

1	1.675619000	-2.673974000	-2.030958000
1	-4.747702000	-2.774202000	-1.255159000
1	2.476777000	1.205406000	3.038406000
7	-1.490933000	-1.925522000	-1.274900000
7	1.273165000	-0.032875000	0.128062000
7	-2.716591000	-0.644800000	0.415791000
6	-0.844300000	-3.377886000	-2.823950000
6	-1.640473000	1.321189000	3.395062000
6	-2.217537000	-3.496562000	-2.677776000
6	-0.304338000	1.613948000	3.645654000
6	-0.373261000	-2.362992000	-1.901517000
6	-1.754625000	0.909931000	2.036015000
6	-2.630290000	-2.565871000	-1.652520000
6	0.406266000	1.374096000	2.432599000
6	0.965486000	-2.005584000	-1.549136000
6	-3.836406000	-2.250189000	-0.988689000
6	1.769091000	1.031204000	2.234597000
6	1.622841000	-1.176342000	-0.588676000
6	-2.882927000	0.221715000	1.452546000
6	-3.873281000	-1.281420000	0.053252000
6	2.136250000	0.202381000	1.177257000
6	2.944414000	-1.551778000	-0.068444000
6	-4.271542000	0.144429000	1.798938000
6	-4.881538000	-0.766571000	0.934861000
6	3.252477000	-0.701193000	1.029932000
5	-1.360033000	-0.950420000	-0.162525000
5	0.054360000	0.970319000	-0.087387000
8	-0.875909000	0.231056000	-0.801578000
6	5.322727000	-1.873413000	1.378814000
6	5.021061000	-2.722922000	0.292729000
6	3.832761000	-2.579472000	-0.419361000
6	4.436879000	-0.869704000	1.760069000
1	3.603734000	-3.258509000	-1.235562000
1	5.722977000	-3.504703000	0.014277000
1	6.253581000	-2.009549000	1.923402000
1	4.660182000	-0.222488000	2.604113000
7	-0.501103000	1.073928000	1.449275000
7	0.453350000	2.357166000	-0.701854000
6	-0.692815000	3.311447000	-0.965991000
6	-1.895397000	3.079440000	-0.023166000
1	-2.669959000	3.811812000	-0.276245000
1	-1.629691000	3.228318000	1.023864000
1	-2.330364000	2.092911000	-0.158668000
6	-0.300340000	4.792187000	-0.664514000
1	0.292259000	5.279479000	-1.434930000
1	-1.208075000	5.395624000	-0.539839000
1	0.261256000	4.822883000	0.274803000
6	-1.313943000	3.169290000	-2.385642000
1	-2.188274000	3.827008000	-2.478043000
1	-0.634463000	3.426551000	-3.198103000

1	-1.638558000	2.133996000	-2.523667000
1	3.138290000	0.956529000	-0.993515000
6	2.947814000	2.023393000	-1.054768000
1	3.764951000	2.443175000	-1.652952000
1	2.999073000	2.453851000	-0.052635000
6	1.582833000	2.369591000	-1.719357000
6	1.885878000	3.753325000	-2.361275000
1	2.700980000	3.608730000	-3.078995000
1	2.230014000	4.472398000	-1.612103000
1	1.058839000	4.190108000	-2.916960000
6	1.352551000	1.380867000	-2.897108000
1	2.278245000	1.264904000	-3.478130000
1	1.047398000	0.399264000	-2.542648000
1	0.573826000	1.734793000	-3.576067000
6	-0.686274000	-1.968983000	1.072200000
8	-0.926484000	-3.171697000	1.033711000
8	-0.046358000	-1.499517000	2.186077000
1	0.099405000	-2.285380000	2.742548000

E = – 1788.2636279 a. u.
H = – 1787.633482 a. u.
G = – 1787.727830 a. u.

Cor_2e – B_2OY_2-corrole, Y_1=Y_2=Ph (c_1)

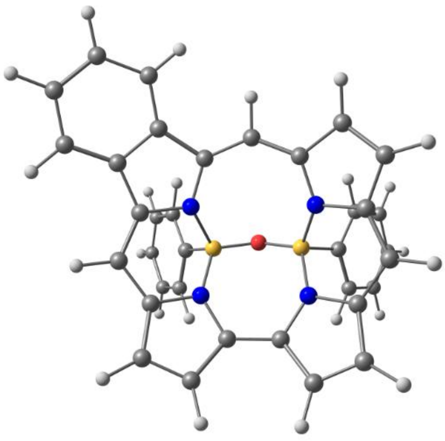

1	-0.716314000	-4.957412000	-0.228441000
1	-0.617762000	3.504867000	3.672575000
1	-3.060432000	-4.593291000	1.049351000
1	2.067684000	3.425413000	3.475784000
1	-2.884629000	2.434080000	3.726334000
1	-4.536207000	0.312097000	3.619570000
1	1.202173000	-3.514068000	-0.915065000
1	-4.295709000	-2.194269000	2.112518000
1	3.808135000	1.649627000	1.900507000
7	-1.451403000	-1.785394000	0.304085000
7	0.485953000	1.547958000	1.255706000
7	1.629074000	-0.346886000	0.232840000
7	-2.161208000	0.308946000	1.308858000
6	-1.188083000	-4.015396000	0.022489000
6	-0.034900000	2.965670000	2.937856000
6	-2.382174000	-3.830814000	0.687749000
6	1.350233000	2.919473000	2.842692000
6	-0.575131000	-2.721210000	-0.174330000
6	-0.570042000	2.063355000	1.966128000
6	-2.534809000	-2.423328000	0.892509000
6	1.666118000	1.980455000	1.818944000
6	0.783726000	-2.580019000	-0.548187000
6	-3.464038000	-1.683981000	1.638736000
6	2.867021000	1.309448000	1.483054000
6	1.763391000	-1.626966000	-0.246726000
6	-1.859572000	1.451442000	1.995468000
6	-3.224056000	-0.326489000	1.922995000
6	2.823499000	0.120317000	0.762508000

6	3.188975000	-1.956997000	-0.153577000
6	-2.865988000	1.629722000	3.003351000
6	-3.712424000	0.534390000	2.953450000
6	3.839924000	-0.870714000	0.490739000
5	-1.699115000	-0.275124000	-0.093824000
5	0.593180000	0.802984000	-0.140714000
8	-0.607045000	0.358123000	-0.681327000
6	5.933529000	-2.047250000	0.377308000
6	5.289638000	-3.129333000	-0.259917000
6	3.919828000	-3.097006000	-0.514860000
6	5.213702000	-0.919717000	0.764418000
1	3.433648000	-3.940578000	-0.997062000
1	5.871184000	-3.999041000	-0.554521000
1	7.002600000	-2.097274000	0.567000000
1	5.708091000	-0.089463000	1.261595000
6	-2.964786000	-0.196756000	-1.138612000
6	-3.845278000	0.897403000	-1.139268000
6	-3.152470000	-1.165470000	-2.137924000
6	-4.865958000	1.024860000	-2.087120000
6	-4.167420000	-1.053847000	-3.094225000
6	-5.032335000	0.045639000	-3.072598000
1	-3.730140000	1.666716000	-0.377747000
1	-2.490907000	-2.029115000	-2.166401000
1	-5.530885000	1.886270000	-2.058607000
1	-4.285149000	-1.823445000	-3.855075000
1	-5.823972000	0.138375000	-3.813174000
6	1.309322000	1.805244000	-1.229237000
6	1.042804000	3.184055000	-1.232780000
6	2.123631000	1.311493000	-2.261182000
6	1.561992000	4.034740000	-2.214256000
6	2.652375000	2.147567000	-3.250003000
6	2.373036000	3.518348000	-3.230633000
1	0.416654000	3.600792000	-0.446010000
1	2.352791000	0.247962000	-2.291748000
1	1.335547000	5.099122000	-2.187045000
1	3.281944000	1.731666000	-4.034521000
1	2.781071000	4.174331000	-3.996689000

$E = -1691.9691735$ a. u.
$H = -1691.440114$ a. u.
$G = -1691.529911$ a. u.

Cor_2e – B_2OY_2-corrole, $Y_1=Y_2=$Ph (t_1)

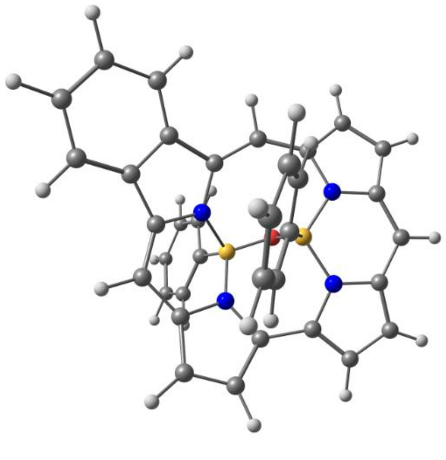

1	-0.374056000	-2.459045000	-4.507573000
1	-2.551710000	1.734823000	3.900888000
1	-3.018368000	-2.782584000	-4.202617000
1	0.060268000	2.055675000	4.456451000
1	-4.884867000	1.460469000	1.923555000
1	-6.032855000	-0.185489000	0.133548000
1	1.618518000	-1.661081000	-2.863219000
1	-4.819469000	-1.892760000	-1.970604000
1	2.467481000	1.419248000	2.799448000
7	-1.488600000	-1.566528000	-1.562801000

7	-0.567885000	1.289227000	1.317965000
7	1.199673000	0.351941000	-0.140488000
7	-2.757016000	-0.404126000	0.264432000
6	-0.955901000	-2.232886000	-3.622518000
6	-1.731616000	1.682252000	3.196377000
6	-2.321789000	-2.403482000	-3.465567000
6	-0.371423000	1.835135000	3.488462000
6	-0.425124000	-1.630935000	-2.416156000
6	-1.849892000	1.269731000	1.835016000
6	-2.669504000	-1.919680000	-2.149985000
6	0.355052000	1.532619000	2.299086000
6	0.925341000	-1.226466000	-2.144244000
6	-3.883030000	-1.607804000	-1.502225000
6	1.731281000	1.245949000	2.021821000
6	1.616467000	-0.548701000	-1.093767000
6	-2.962022000	0.592108000	1.179700000
6	-3.924603000	-0.803866000	-0.327572000
6	2.118412000	0.576667000	0.856759000
6	3.022485000	-0.814623000	-0.750814000
6	-4.380434000	0.752370000	1.279142000
6	-4.975410000	-0.100659000	0.349860000
6	3.326056000	-0.128454000	0.463108000
5	-1.374484000	-0.742557000	-0.300504000
5	-0.091217000	1.242608000	-0.174590000
8	-0.999747000	0.528375000	-0.917540000
6	5.545531000	-1.054657000	0.436951000
6	5.248223000	-1.737497000	-0.759369000
6	3.987454000	-1.635016000	-1.346891000
6	4.585241000	-0.258323000	1.059305000
1	3.759277000	-2.189419000	-2.252851000
1	6.008805000	-2.360222000	-1.223282000
1	6.531940000	-1.158009000	0.881768000
1	4.809198000	0.253300000	1.991579000
6	-0.570054000	-1.719395000	0.827027000
6	-0.631446000	-1.556531000	2.227761000
6	0.082781000	-2.905556000	0.417389000
6	-0.038454000	-2.441155000	3.133961000
6	0.689853000	-3.799494000	1.305165000
6	0.647444000	-3.568509000	2.680683000
1	-1.167454000	-0.724614000	2.656705000
1	0.128708000	-3.158411000	-0.633467000
1	-0.118120000	-2.238342000	4.200257000
1	1.194569000	-4.680210000	0.912526000
1	1.122204000	-4.254513000	3.378832000
6	0.256823000	2.723342000	-0.752240000
6	0.033462000	3.918608000	-0.050931000
6	0.774488000	2.833734000	-2.056336000
6	0.309812000	5.169172000	-0.618141000
6	1.056745000	4.073589000	-2.635527000
6	0.825213000	5.251987000	-1.914867000
1	-0.367147000	3.872094000	0.959235000
1	0.950360000	1.924045000	-2.626436000
1	0.123805000	6.077590000	-0.047906000
1	1.454621000	4.124460000	-3.647444000
1	1.042820000	6.220691000	-2.359909000

E = – 1691.9318173 a. u.
H = – 1691.402459 a. u.
G = – 1691.489944 a. u.

Cor_2f – B_2OY_2-corrole, Y_1=Y_2= C≡C–C≡C–Ph (c_1)

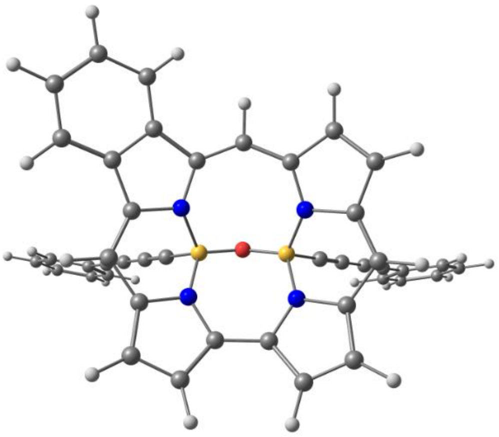

1	-1.604390000	-4.023806000	-3.606989000
1	1.038233000	-2.212526000	5.136484000
1	-3.625926000	-4.699207000	-1.960184000
1	3.579968000	-2.271301000	4.250779000
1	-1.400459000	-2.734117000	4.951165000
1	-3.536131000	-3.790479000	3.703708000
1	0.531085000	-2.746173000	-3.406649000
1	-4.100963000	-4.070342000	0.833995000
1	4.643928000	-2.150629000	1.506864000
7	-1.448226000	-2.560219000	-0.656167000
7	1.352521000	-1.480011000	1.936033000
7	1.863736000	-1.844316000	-0.415994000
7	-1.506819000	-2.081907000	1.720386000
6	-1.791538000	-3.646638000	-2.609347000
6	1.385181000	-1.972771000	4.140479000
6	-2.820640000	-4.003482000	-1.762022000
6	2.696010000	-2.011054000	3.683328000
6	-0.896866000	-2.770340000	-1.893047000
6	0.539174000	-1.682958000	3.026130000
6	-2.590573000	-3.339380000	-0.518162000
6	2.657400000	-1.756585000	2.283040000
6	0.404469000	-2.469794000	-2.362994000
6	-3.222455000	-3.442953000	0.730157000
6	3.608345000	-1.954843000	1.252804000
6	1.618541000	-2.211329000	-1.718990000
6	-0.850440000	-1.982545000	2.917065000
6	-2.622371000	-2.885361000	1.873990000
6	3.189192000	-2.070284000	-0.068721000
6	2.916348000	-2.566175000	-2.297966000
6	-1.664295000	-2.625962000	3.907886000
6	-2.760851000	-3.175524000	3.265396000
6	3.885593000	-2.492789000	-1.261925000
6	5.589241000	-3.224680000	-2.791110000
6	4.628293000	-3.301815000	-3.822088000
6	3.292483000	-2.989000000	-3.580489000
6	5.223914000	-2.830649000	-1.506948000
1	2.561031000	-3.060899000	-4.380490000
1	4.936737000	-3.609728000	-4.817652000
1	6.624652000	-3.475987000	-3.005325000
1	5.962073000	-2.777446000	-0.711324000
5	-1.340919000	-1.345838000	0.334464000
8	-0.208314000	-0.555589000	0.188792000
5	1.109637000	-0.821206000	0.525818000
6	1.936612000	0.574102000	0.466748000
6	2.525944000	1.647466000	0.400399000
6	3.175839000	2.843125000	0.325526000
6	3.761892000	3.915640000	0.258547000

6	4.429995000	5.167619000	0.178137000
6	3.700920000	6.378447000	0.185052000
1	2.618451000	6.337147000	0.252595000
6	4.359119000	7.604652000	0.105356000
1	3.779989000	8.524358000	0.111696000
6	5.754920000	7.656822000	0.016805000
1	6.264493000	8.614356000	-0.045746000
6	6.488607000	6.465101000	0.008680000
6	5.838776000	5.234295000	0.088135000
1	6.407289000	4.309677000	0.081262000
1	7.572956000	6.494803000	-0.060132000
6	-2.648338000	-0.410760000	0.111198000
6	-3.622138000	0.312339000	-0.070086000
6	-4.705897000	1.113764000	-0.271552000
6	-5.683825000	1.827463000	-0.452418000
6	-6.814301000	2.662885000	-0.662608000
6	-6.694874000	4.070249000	-0.610267000
1	-5.721850000	4.505730000	-0.406366000
6	-7.806138000	4.885981000	-0.817489000
1	-7.692806000	5.966031000	-0.773437000
6	-9.060501000	4.324024000	-1.081188000
1	-9.924283000	4.963035000	-1.242316000
1	-10.162064000	2.485189000	-1.340100000
6	-9.192404000	2.931825000	-1.135806000
6	-8.086560000	2.108354000	-0.929818000
1	-8.188378000	1.028518000	-0.972290000

E = – 1996.6325699 a. u.
H = – 1996.053263 a. u.
G = – 1996.166541 a. u.

Por_1b – B_2OY_2-porphyrin, Y_1=Y_2=OH (c_1)

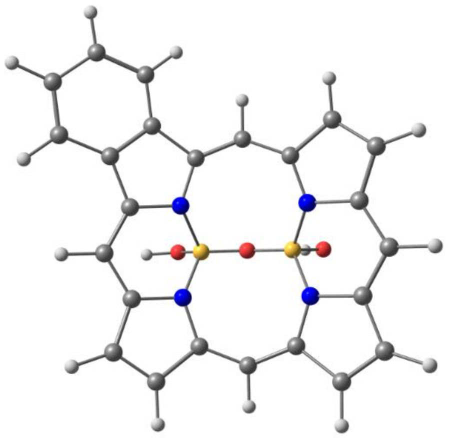

5	-0.198548000	0.956659000	1.065941000
5	1.783553000	-0.727175000	0.893992000
8	0.822297000	0.110982000	1.415741000
6	1.032662000	4.215628000	-0.430243000
6	1.371826000	2.873828000	-0.007362000
6	2.707261000	2.455766000	0.035723000
6	3.337232000	1.214634000	-0.154725000
6	4.680348000	1.136825000	-0.688004000
6	4.909993000	-0.163421000	-1.056934000
6	3.729348000	-0.900429000	-0.715761000
6	3.429961000	-2.256234000	-0.900903000
6	2.172500000	-2.755843000	-0.570872000
6	1.623219000	-4.070670000	-0.745373000
6	0.338599000	-4.024340000	-0.275957000
6	0.071699000	-2.665078000	0.156599000
6	-1.242798000	-2.244997000	0.434326000
6	-1.908642000	-1.023643000	0.322845000
6	-3.347542000	-0.962260000	0.036784000

6	-3.639455000	0.367361000	-0.341080000
6	-2.397480000	1.102972000	-0.227857000
6	-2.127266000	2.440692000	-0.494506000
6	-0.820476000	2.942645000	-0.391473000
6	-0.311757000	4.248283000	-0.694463000
7	0.206721000	2.140792000	0.050142000
7	2.807272000	-0.048836000	-0.149857000
7	1.216395000	-1.938019000	0.000344000
7	-1.408889000	0.249985000	0.215229000
1	1.755186000	5.005473000	-0.589107000
1	5.329971000	1.989706000	-0.835313000
1	5.787550000	-0.574446000	-1.537437000
1	2.140031000	-4.909846000	-1.191166000
1	-0.398344000	-4.816701000	-0.287956000
1	-0.894506000	5.071734000	-1.084803000
1	3.404760000	3.287825000	-0.020658000
1	4.170817000	-2.913064000	-1.342090000
1	-1.924893000	-3.086239000	0.515601000
1	-2.923395000	3.092573000	-0.834708000
6	-4.928907000	0.742608000	-0.735573000
6	-5.927495000	-0.228004000	-0.728481000
1	-6.939566000	0.037246000	-1.018891000
6	-5.641209000	-1.555092000	-0.349698000
1	-6.438714000	-2.292035000	-0.349409000
6	-4.353212000	-1.936353000	0.021205000
1	-4.148479000	-2.964880000	0.302168000
1	-5.148213000	1.762915000	-1.036027000
8	-0.771210000	1.519597000	2.260566000
1	-1.468270000	2.164180000	2.104675000
8	2.596638000	-1.326218000	1.927205000
1	2.288288000	-0.993468000	2.777049000

E = – 1418.9766686 a. u.
H = – 1418.586965 a. u.
G = – 1418.660407 a. u.

Por_1b – B_2OY_2-porphyrin, Y_1=Y_2=OH (t_1)

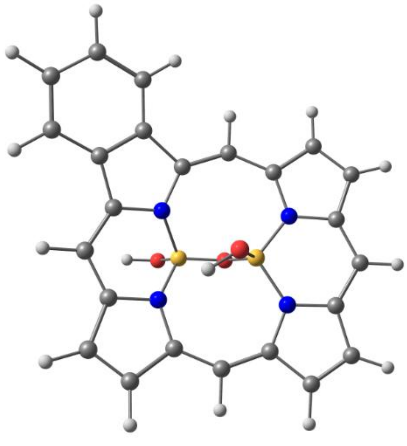

5	-0.132211000	0.878251000	1.127340000
5	1.406641000	-0.475294000	-0.140048000
8	0.936820000	0.025060000	1.102847000
6	0.944522000	4.212626000	-0.395161000
6	1.321114000	2.860069000	-0.033831000
6	2.666083000	2.438554000	-0.086305000
6	3.376326000	1.220455000	-0.159887000
6	4.821415000	1.147117000	-0.266769000
6	5.175921000	-0.174325000	-0.356885000
6	3.953887000	-0.937624000	-0.309636000

6	3.731466000	-2.326382000	-0.293899000
6	2.443406000	-2.847198000	-0.131587000
6	1.968180000	-4.201898000	0.022009000
6	0.614489000	-4.128161000	0.218052000
6	0.227513000	-2.726726000	0.190997000
6	-1.109897000	-2.288077000	0.319804000
6	-1.822967000	-1.076995000	0.281549000
6	-3.269179000	-1.054179000	0.002246000
6	-3.652871000	0.298967000	-0.150654000
6	-2.451665000	1.085547000	0.058205000
6	-2.226257000	2.454527000	-0.104393000
6	-0.919347000	2.980653000	-0.103665000
6	-0.424965000	4.282746000	-0.453075000
7	0.144990000	2.163694000	0.180033000
7	2.911608000	-0.064057000	-0.208541000
7	1.364653000	-2.014158000	-0.036170000
7	-1.421864000	0.233897000	0.369262000
1	1.650312000	4.994322000	-0.644628000
1	5.479873000	2.005799000	-0.262527000
1	6.171142000	-0.590439000	-0.434833000
1	2.587358000	-5.088275000	-0.002869000
1	-0.072004000	-4.948984000	0.378634000
1	-1.032681000	5.129119000	-0.743641000
1	3.337931000	3.289261000	-0.179358000
1	4.576258000	-3.001503000	-0.366687000
1	-1.783831000	-3.139650000	0.366852000
1	-3.058885000	3.105583000	-0.344615000
6	-5.903124000	-0.372373000	-0.626443000
6	-4.967102000	0.648979000	-0.475062000
6	-5.527229000	-1.720842000	-0.475230000
1	-5.253852000	1.688565000	-0.603489000
1	-6.274022000	-2.499144000	-0.600409000
6	-4.211874000	-2.073039000	-0.174380000
1	-3.937997000	-3.119210000	-0.079155000
1	-6.933411000	-0.128629000	-0.867470000
8	0.741719000	-0.163100000	-1.384239000
1	0.454951000	0.754167000	-1.439691000
8	-0.501606000	1.286015000	2.444534000
1	-1.241539000	1.900278000	2.474193000

E = – 1418.9859712 a. u.
H = – 1418.595925 a. u.
G = – 1418.668080 a. u.

Por_1c – B_2OY_2- porphyrin, Y_1=NH_2; Y_2=CO_2H (c_1)

5	-0.397045000	0.999303000	0.951475000
5	1.673466000	-0.554655000	0.542284000

8	0.696208000	0.175554000	1.204407000
6	0.507001000	4.376517000	-0.429161000
6	0.966689000	3.045495000	-0.095041000
6	2.324769000	2.722710000	-0.141069000
6	3.027879000	1.538963000	-0.438557000
6	4.327117000	1.587153000	-1.065315000
6	4.626313000	0.327799000	-1.523264000
6	3.536313000	-0.514556000	-1.147984000
6	3.324633000	-1.876807000	-1.384979000
6	2.134601000	-2.488380000	-1.001491000
6	1.656840000	-3.817669000	-1.221655000
6	0.405774000	-3.883837000	-0.663865000
6	0.082399000	-2.580291000	-0.127167000
6	-1.239227000	-2.262902000	0.253056000
6	-1.995806000	-1.093229000	0.230083000
6	-3.451911000	-1.128893000	0.034432000
6	-3.858565000	0.184996000	-0.288397000
6	-2.667776000	1.007078000	-0.222084000
6	-2.513850000	2.371204000	-0.443168000
6	-1.244360000	2.964389000	-0.376445000
6	-0.846409000	4.318350000	-0.633008000
7	-0.138781000	2.223490000	-0.027450000
7	2.593244000	0.237887000	-0.468762000
7	1.168838000	-1.771659000	-0.309800000
7	-1.596498000	0.220018000	0.138272000
1	1.160007000	5.226367000	-0.578989000
1	4.900848000	2.493388000	-1.207934000
1	5.497099000	0.010900000	-2.080399000
1	2.192631000	-4.593858000	-1.750349000
1	-0.278967000	-4.721502000	-0.680064000
1	-1.505883000	5.112939000	-0.954783000
1	2.961771000	3.602430000	-0.184984000
1	4.082843000	-2.453242000	-1.901386000
1	-1.853024000	-3.154947000	0.338319000
1	-3.372352000	2.975516000	-0.711074000
6	-5.191915000	0.476350000	-0.597619000
6	-6.118320000	-0.563266000	-0.560037000
1	-7.161786000	-0.364321000	-0.784691000
6	-5.717749000	-1.874326000	-0.234688000
1	-6.460054000	-2.666374000	-0.208319000
6	-4.385853000	-2.171345000	0.050643000
1	-4.093709000	-3.189039000	0.290899000
1	-5.499239000	1.485017000	-0.857360000
7	-0.905954000	1.542505000	2.254408000
1	-0.984686000	0.840109000	2.981770000
1	-1.749391000	2.103188000	2.244364000
6	2.696265000	-1.147837000	1.743337000
8	3.607265000	-1.946253000	1.610260000

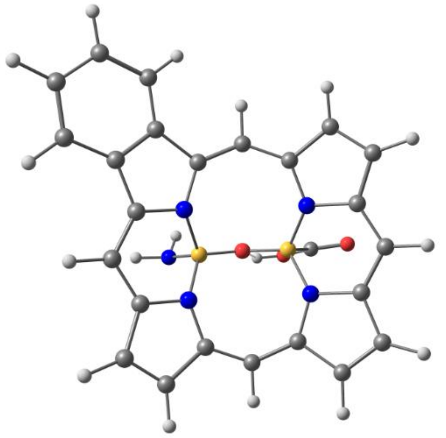

8	2.440930000	-0.625181000	2.975230000
1	1.671497000	-0.034455000	2.850998000

E = – 1512.4110618 a. u.
H = – 1511.996358 a. u.
G = – 1512.074151 a. u.

Por_1c – B_2OY_2- porphyrin, Y_1=NH_2; Y_2=CO_2H (t_1)

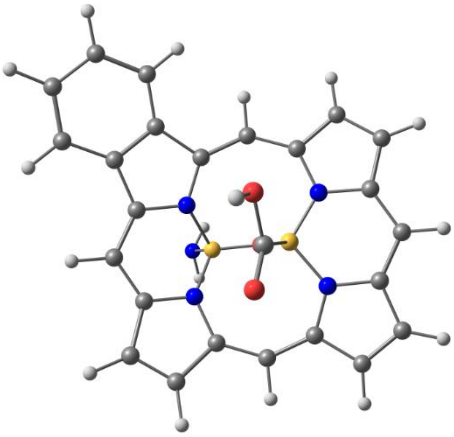

5	-0.168287000	0.900307000	1.272033000
5	1.387438000	-0.500949000	-0.009108000
8	0.885410000	-0.000812000	1.211699000
6	1.007646000	4.184434000	-0.240173000
6	1.344707000	2.838254000	0.183159000
6	2.681650000	2.390562000	0.153996000
6	3.365972000	1.159988000	0.047427000
6	4.807209000	1.056235000	-0.032989000
6	5.138381000	-0.269331000	-0.172331000
6	3.905199000	-1.009200000	-0.179511000
6	3.652939000	-2.393128000	-0.197902000
6	2.355959000	-2.888554000	-0.022724000
6	1.863070000	-4.227786000	0.176511000
6	0.518154000	-4.125610000	0.424708000
6	0.149859000	-2.723147000	0.378133000
6	-1.176715000	-2.262867000	0.535133000
6	-1.871357000	-1.040070000	0.458302000
6	-3.308281000	-0.996779000	0.148248000
6	-3.659004000	0.359702000	-0.056672000
6	-2.451320000	1.130258000	0.162459000
6	-2.196158000	2.488926000	-0.017645000
6	-0.885278000	2.997097000	0.042823000
6	-0.355949000	4.273626000	-0.351340000
7	0.152952000	2.184872000	0.405016000
7	2.878329000	-0.114833000	-0.066619000
7	1.290782000	-2.033470000	0.083446000
7	-1.447274000	0.264130000	0.515689000
1	1.737642000	4.937011000	-0.507835000
1	5.483709000	1.898957000	0.021301000
1	6.127061000	-0.701556000	-0.244634000
1	2.466287000	-5.125128000	0.152270000
1	-0.173847000	-4.931171000	0.632174000
1	-0.939209000	5.111711000	-0.707745000
1	3.373378000	3.227185000	0.082080000
1	4.483279000	-3.084677000	-0.279458000
1	-1.862398000	-3.102151000	0.618092000
1	-3.005230000	3.147459000	-0.311318000
6	-5.910713000	-0.282769000	-0.566231000
6	-4.958882000	0.724179000	-0.423003000

6	-5.566380000	-1.634099000	-0.365630000
1	-5.221615000	1.764865000	-0.587814000
1	-6.326201000	-2.400598000	-0.485518000
6	-4.266661000	-2.002748000	-0.022388000
1	-4.015251000	-3.050576000	0.110011000
1	-6.930046000	-0.026560000	-0.839029000
7	-0.642542000	1.351312000	2.625697000
1	0.020349000	1.975704000	3.076716000
1	-0.784502000	0.563942000	3.253096000
6	0.758421000	-0.029589000	-1.494201000
8	1.113928000	0.937878000	-2.148206000
8	-0.174127000	-0.881822000	-2.031864000
1	-0.411375000	-0.512755000	-2.901620000

E = – 1512.4145479 a. u.
H = – 1512.000385 a. u.
G = – 1512.078329 a. u.

Por_1d – B_2OY_2- porphyrin, Y_1=$N(^tBu)_2$; Y_2=CO_2H (c_1)

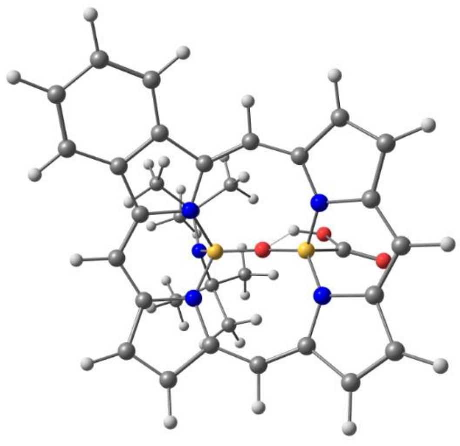

5	0.357382000	-0.775365000	0.003174000
5	-1.971563000	0.541228000	0.372356000
8	-0.844262000	-0.257304000	0.506810000
6	-0.395774000	-2.781453000	-3.257461000
6	-0.928541000	-1.927894000	-2.215452000
6	-2.306546000	-1.704148000	-2.182214000
6	-3.166329000	-0.711003000	-1.689476000
6	-4.491712000	-0.548727000	-2.241072000
6	-4.996739000	0.643532000	-1.792555000
6	-3.998630000	1.217387000	-0.947549000
6	-3.974195000	2.468998000	-0.323181000
6	-2.811440000	2.922829000	0.289328000
6	-2.470069000	4.209527000	0.814777000
6	-1.144473000	4.153346000	1.156044000
6	-0.648959000	2.832134000	0.826417000
6	0.734657000	2.585418000	0.762600000
6	1.565941000	1.702596000	0.074092000
6	2.892247000	2.160114000	-0.361273000
6	3.434026000	1.153221000	-1.185071000
6	2.458641000	0.089455000	-1.217264000
6	2.476642000	-1.072178000	-1.970797000
6	1.280905000	-1.762323000	-2.181473000
6	0.966650000	-2.675665000	-3.239033000
7	0.137521000	-1.394147000	-1.511230000
7	-2.914093000	0.361048000	-0.872990000
7	-1.698948000	2.097479000	0.360835000
7	1.381834000	0.425830000	-0.419688000
1	-1.007954000	-3.328161000	-3.962467000

1	-4.945446000	-1.235931000	-2.942883000
1	-5.945141000	1.102196000	-2.035473000
1	-3.139516000	5.056410000	0.876579000
1	-0.525268000	4.958706000	1.528550000
1	1.684346000	-3.111408000	-3.920536000
1	-2.832406000	-2.380036000	-2.851472000
1	-4.844169000	3.111945000	-0.379613000
1	1.290070000	3.435355000	1.147269000
1	3.358343000	-1.332951000	-2.543640000
6	4.684708000	1.303904000	-1.795025000
6	5.396075000	2.474964000	-1.551280000
1	6.375223000	2.615214000	-1.998985000
6	4.858191000	3.487283000	-0.730389000
1	5.432269000	4.392413000	-0.556718000
6	3.602797000	3.348055000	-0.143543000
1	3.200563000	4.145843000	0.472765000
1	5.093221000	0.527731000	-2.435040000
7	1.036531000	-1.819214000	0.952522000
6	-2.914635000	0.264878000	1.746408000
8	-4.103376000	0.482119000	1.896684000
8	-2.194515000	-0.226149000	2.794477000
1	-1.304519000	-0.406899000	2.439009000
6	1.981123000	-1.354189000	2.059499000
6	0.276187000	-3.105319000	1.255377000
6	1.295088000	-0.336729000	3.009865000
1	2.021706000	0.084286000	3.715945000
1	0.866128000	0.495073000	2.448917000
1	0.498461000	-0.795704000	3.599137000
6	3.274234000	-0.683566000	1.519296000
1	3.150334000	0.360710000	1.259679000
1	4.038776000	-0.697465000	2.301938000
1	3.665712000	-1.236116000	0.663632000
6	2.604065000	-2.483731000	2.939350000
1	3.144188000	-1.994421000	3.756111000
1	1.910422000	-3.178189000	3.399708000
1	3.336845000	-3.058962000	2.366712000
6	-0.431153000	-3.148620000	2.647482000
1	-0.735919000	-4.180285000	2.859535000
1	0.161577000	-2.815115000	3.492620000
1	-1.340426000	-2.542750000	2.630980000
6	1.199610000	-4.340202000	1.039095000
1	1.678555000	-4.250446000	0.058788000
1	1.980780000	-4.466561000	1.783087000
1	0.600574000	-5.258994000	1.044144000
6	-0.906113000	-3.390345000	0.301455000
1	-1.608882000	-2.561223000	0.248505000
1	-0.574984000	-3.670079000	-0.696886000
1	-1.454116000	-4.249085000	0.700698000

E = – 1826.9007182 a. u.
H = – 1826.249616 a. u.
G = – 1826.347994 a. u.

Por_1d – B_2OY_2- porphyrin, Y_1=N(^{t}Bu)$_2$; Y_2=CO_2H (t_1)

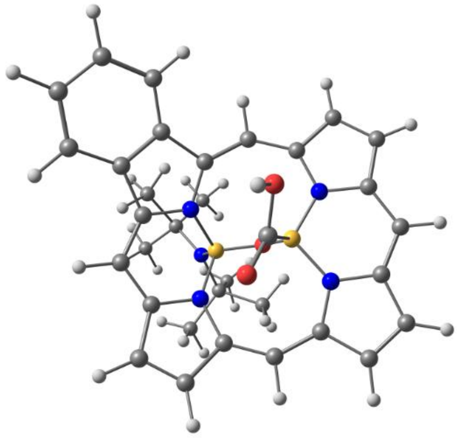

5	0.135846000	0.838675000	-0.197837000
5	-1.496202000	-1.044989000	0.104857000
8	-0.944880000	0.053233000	-0.581514000
6	-1.047101000	2.570732000	3.125337000
6	-1.396163000	1.750034000	1.976568000
6	-2.731618000	1.334473000	1.834387000
6	-3.442178000	0.306677000	1.178707000
6	-4.880745000	0.185384000	1.231876000
6	-5.247619000	-0.910110000	0.489225000
6	-4.039286000	-1.488822000	-0.026510000
6	-3.844381000	-2.575981000	-0.894088000
6	-2.565951000	-2.896834000	-1.359330000
6	-2.104427000	-3.851612000	-2.335542000
6	-0.747097000	-3.685267000	-2.446002000
6	-0.348088000	-2.625421000	-1.541059000
6	0.987900000	-2.254358000	-1.286048000
6	1.665038000	-1.385219000	-0.411247000
6	2.998190000	-1.740404000	0.086137000
6	3.360695000	-0.781359000	1.058417000
6	2.271910000	0.166536000	1.120709000
6	2.084186000	1.239191000	1.986484000
6	0.814086000	1.808830000	2.134490000
6	0.313328000	2.592528000	3.233468000
7	-0.220786000	1.415340000	1.330823000
7	-2.981748000	-0.738269000	0.424127000
7	-1.477927000	-2.201354000	-0.911009000
7	1.319955000	-0.190312000	0.198414000
1	-1.770913000	2.989471000	3.811999000
1	-5.531352000	0.871479000	1.757796000
1	-6.246128000	-1.279519000	0.299411000
1	-2.733100000	-4.546953000	-2.874880000
1	-0.067855000	-4.229464000	-3.088762000
1	0.917336000	3.031051000	4.015996000
1	-3.395818000	1.924095000	2.461958000
1	-4.702589000	-3.138613000	-1.242291000
1	1.676505000	-2.939689000	-1.773952000
1	2.873491000	1.512083000	2.676886000
6	5.418255000	-1.938433000	1.462732000
6	4.567651000	-0.877950000	1.760696000
6	5.060325000	-2.900645000	0.495806000
1	4.835464000	-0.144957000	2.515734000

1	5.739835000	-3.720935000	0.284806000
6	3.848066000	-2.821223000	-0.184831000
1	3.578758000	-3.584100000	-0.908439000
1	6.366637000	-2.032190000	1.983122000
7	0.510727000	1.976060000	-1.170348000
6	-0.848095000	-1.609819000	1.550037000
8	-0.883334000	-1.058076000	2.637942000
8	-0.312138000	-2.874196000	1.474641000
1	-0.027632000	-3.100980000	2.378406000
1	1.194691000	0.584556000	-3.406922000
6	1.550051000	1.619622000	-3.449589000
1	2.494377000	1.636273000	-4.007999000
6	1.770622000	2.124134000	-1.991296000
6	2.349508000	3.571144000	-1.949960000
1	1.670660000	4.361638000	-2.250988000
1	3.221608000	3.632157000	-2.610451000
6	2.995393000	1.302015000	-1.523996000
1	3.841328000	1.584404000	-2.157130000
1	3.281113000	1.527114000	-0.498617000
1	2.866865000	0.230892000	-1.652182000
1	2.681624000	3.795496000	-0.930414000
1	0.826449000	2.193887000	-4.018731000
1	-2.177148000	1.610477000	-1.928485000
6	-2.014939000	2.463040000	-1.268882000
1	-2.720735000	3.258385000	-1.533092000
6	-0.576934000	3.022271000	-1.420582000
6	-0.426883000	4.195118000	-0.411864000
1	-0.450146000	3.827338000	0.613996000
1	-1.247817000	4.914293000	-0.528560000
6	-0.660642000	3.625705000	-2.858930000
1	-1.406634000	4.426470000	-2.830764000
1	0.249196000	4.065827000	-3.255342000
1	-1.018690000	2.878851000	-3.572574000
1	0.510552000	4.735776000	-0.546272000
1	-2.255204000	2.155143000	-0.262878000

$E = -1826.9086727$ a. u.
$H = -1826.257890$ a. u.
$G = -1826.355203$ a. u.

Por_1e – B_2OY_2- porphyrin, Y_1=Y_2=Ph (c_1)

5	-0.652027000	0.809299000	0.070856000
5	1.706632000	-0.333511000	-0.047860000
8	0.589087000	0.327792000	0.426962000
6	-0.482101000	3.884807000	-2.137525000
6	0.241871000	2.798297000	-1.509967000
6	1.640544000	2.753918000	-1.558467000

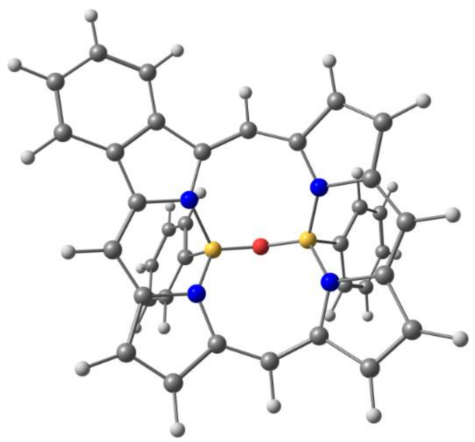

6	2.576380000	1.705932000	-1.600497000
6	3.839135000	1.873186000	-2.289265000
6	4.393533000	0.631344000	-2.459443000
6	3.499565000	-0.303317000	-1.843792000
6	3.559457000	-1.700220000	-1.781607000
6	2.509577000	-2.430506000	-1.229050000
6	2.298758000	-3.847911000	-1.167603000
6	1.090794000	-4.038905000	-0.551939000
6	0.524083000	-2.734989000	-0.262733000
6	-0.824690000	-2.612209000	0.123875000
6	-1.806091000	-1.640843000	-0.079837000
6	-3.228980000	-1.998003000	-0.155390000
6	-3.910026000	-0.882062000	-0.690291000
6	-2.912180000	0.147897000	-0.882919000
6	-3.039281000	1.424052000	-1.414607000
6	-1.913540000	2.239216000	-1.592975000
6	-1.801943000	3.525932000	-2.214340000
7	-0.672363000	1.833300000	-1.154976000
7	2.428537000	0.377057000	-1.301302000
7	1.429647000	-1.787568000	-0.646677000
7	-1.688511000	-0.327648000	-0.458517000
1	-0.015095000	4.772960000	-2.542670000
1	4.207380000	2.817883000	-2.667348000
1	5.306263000	0.374388000	-2.979625000
1	2.970205000	-4.596784000	-1.565207000
1	0.582023000	-4.977080000	-0.373267000
1	-2.617019000	4.073441000	-2.667521000
1	2.073773000	3.705341000	-1.856710000
1	4.406387000	-2.220499000	-2.213644000
1	-1.244103000	-3.569439000	0.419288000
1	-4.010444000	1.777904000	-1.739266000
6	-5.287150000	-0.921558000	-0.935855000
6	-5.977359000	-2.089237000	-0.620894000
1	-7.048842000	-2.144026000	-0.787581000
6	-5.302050000	-3.204559000	-0.085921000
1	-5.864609000	-4.101406000	0.155538000
6	-3.926704000	-3.175391000	0.138005000
1	-3.421873000	-4.046025000	0.545289000
1	-5.806447000	-0.064085000	-1.353114000
6	-1.361083000	1.501821000	1.375087000
6	-1.746305000	0.685723000	2.453044000
6	-1.532657000	2.885888000	1.530545000
6	-2.285186000	1.218130000	3.626211000
6	-2.066689000	3.435518000	2.702127000
6	-2.449724000	2.601716000	3.755018000
1	-1.610128000	-0.391232000	2.379839000
1	-1.239414000	3.560198000	0.729660000
1	-2.571317000	0.557824000	4.441350000

1	-2.182293000	4.513173000	2.791352000
1	-2.866195000	3.022807000	4.666287000
6	2.815918000	-0.446150000	1.152071000
6	3.112337000	-1.634559000	1.837149000
6	3.456928000	0.723439000	1.596789000
6	4.007922000	-1.662426000	2.912701000
6	4.355459000	0.711771000	2.665989000
6	4.636852000	-0.487234000	3.330070000
1	2.634733000	-2.564394000	1.537481000
1	3.239808000	1.668513000	1.103531000
1	4.212830000	-2.600455000	3.423420000
1	4.831950000	1.635470000	2.985652000
1	5.333294000	-0.502774000	4.164312000

E = – 1730.5885956 a. u.
H = – 1730.038996 a. u.
G = – 1730.130308 a. u.

Por_1e – B_2OY_2- porphyrin, Y_1=Y_2=Ph (t_1)

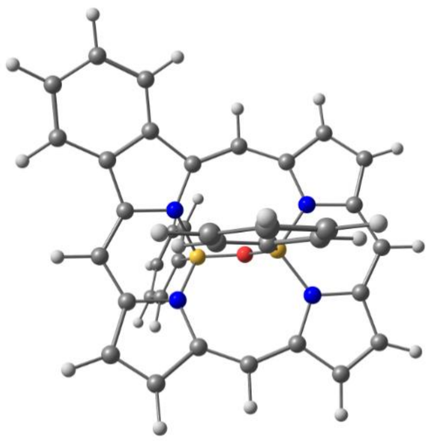

5	0.476491000	1.110517000	-0.187178000
5	-1.585371000	-0.277317000	-0.314007000
8	-0.690901000	0.712669000	-0.787134000
6	-0.436701000	2.737623000	3.121161000
6	-0.892196000	2.152815000	1.871786000
6	-2.256900000	2.202898000	1.504673000
6	-3.143358000	1.560246000	0.609496000
6	-4.526249000	1.957648000	0.422976000
6	-5.082663000	1.139148000	-0.528077000
6	-4.056282000	0.211313000	-0.933328000
6	-4.037560000	-0.787378000	-1.926869000
6	-2.856041000	-1.473284000	-2.236840000
6	-2.532659000	-2.410638000	-3.287060000
6	-1.192044000	-2.676488000	-3.184373000
6	-0.657507000	-1.899691000	-2.076587000
6	0.707271000	-1.918513000	-1.696341000
6	1.573320000	-1.282962000	-0.781953000
6	2.891066000	-1.854814000	-0.452920000
6	3.467894000	-1.054658000	0.563490000
6	2.511655000	-0.001740000	0.842722000
6	2.469392000	0.951835000	1.863058000
6	1.285504000	1.666906000	2.140908000
6	0.889222000	2.429908000	3.292224000
7	0.212443000	1.559806000	1.299755000
7	-2.936466000	0.484532000	-0.206566000
7	-1.711428000	-1.235402000	-1.529097000
7	1.449517000	-0.151467000	-0.013270000
1	-1.079702000	3.265013000	3.813607000

1	-5.000422000	2.783357000	0.937024000
1	-6.086979000	1.175859000	-0.927606000
1	-3.230189000	-2.796586000	-4.017945000
1	-0.600376000	-3.321839000	-3.820258000
1	1.520574000	2.665642000	4.138033000
1	-2.793735000	2.912743000	2.131013000
1	-4.936416000	-0.985274000	-2.499181000
1	1.232989000	-2.697692000	-2.242801000
1	3.313017000	1.045927000	2.536705000
6	5.386014000	-2.488469000	0.629992000
6	4.712905000	-1.369742000	1.116378000
6	4.815917000	-3.290120000	-0.377567000
1	5.147003000	-0.756686000	1.900501000
1	5.358317000	-4.159290000	-0.737278000
6	3.565372000	-2.989825000	-0.916360000
1	3.134763000	-3.630228000	-1.679889000
1	6.359665000	-2.749659000	1.033384000
6	1.273815000	2.261346000	-1.010787000
6	1.835862000	1.947269000	-2.261002000
6	1.382030000	3.591328000	-0.575546000
6	2.476173000	2.911303000	-3.043131000
6	2.018659000	4.569057000	-1.348757000
6	2.570545000	4.230980000	-2.586885000
1	1.764397000	0.927190000	-2.632932000
1	0.958727000	3.877200000	0.384924000
1	2.900035000	2.637435000	-4.006359000
1	2.084130000	5.591775000	-0.985093000
1	3.067700000	4.985634000	-3.190729000
6	-1.491965000	-1.250029000	1.032464000
6	-2.588099000	-2.133933000	1.170222000
6	-0.518607000	-1.346439000	2.040147000
6	-2.708633000	-3.040579000	2.223562000
6	-0.621175000	-2.250638000	3.105036000
6	-1.717095000	-3.105785000	3.206929000
1	-3.383492000	-2.120317000	0.430065000
1	0.351191000	-0.713588000	2.021324000
1	-3.575439000	-3.694929000	2.276246000
1	0.165642000	-2.279019000	3.855057000
1	-1.799539000	-3.807686000	4.032398000

E = – 1730.5904199 a. u.
H = – 1730.040100 a. u.
G = – 1730.130549 a. u.

Por_1f – B_2OY_2-corrole, Y_1=Y_2= C≡C–C≡C–Ph (c_1)

5	1.185142000	-0.955988000	0.605408000
5	-1.351059000	-1.483305000	0.386005000

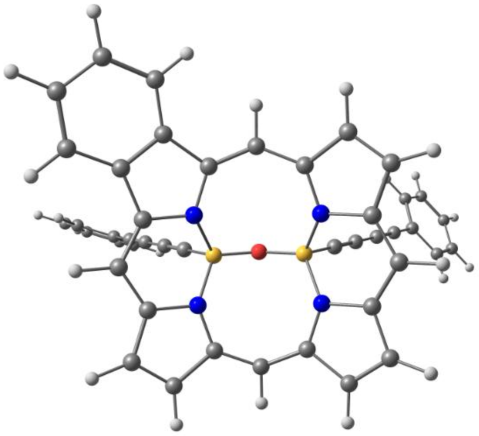

8	-0.166575000	-0.776939000	0.423275000
6	2.117483000	-1.591134000	4.208324000
6	1.053188000	-1.501494000	3.232533000
6	-0.287794000	-1.541921000	3.629632000
6	-1.458751000	-2.023217000	3.019119000
6	-2.537128000	-2.570193000	3.810390000
6	-3.380776000	-3.246132000	2.965182000
6	-2.854360000	-3.085911000	1.644976000
6	-3.313495000	-3.590268000	0.421708000
6	-2.586491000	-3.382578000	-0.747922000
6	-2.809907000	-3.864787000	-2.078831000
6	-1.782531000	-3.387592000	-2.848617000
6	-0.889866000	-2.636645000	-1.988818000
6	0.391664000	-2.262622000	-2.435907000
6	1.622098000	-2.097509000	-1.799482000
6	2.886648000	-2.360374000	-2.494968000
6	3.899193000	-2.422375000	-1.511324000
6	3.255990000	-2.146313000	-0.246282000
6	3.789645000	-2.095849000	1.034677000
6	2.963405000	-1.876289000	2.145067000
6	3.285129000	-1.852393000	3.540022000
7	1.615917000	-1.639014000	1.982543000
7	-1.707615000	-2.318155000	1.702887000
7	-1.430271000	-2.618268000	-0.733602000
7	1.912558000	-1.916314000	-0.469813000
1	1.966960000	-1.545476000	5.278941000
1	-2.591372000	-2.513844000	4.889621000
1	-4.260761000	-3.820013000	3.221950000
1	-3.626842000	-4.505561000	-2.381765000
1	-1.594700000	-3.587593000	-3.895304000
1	4.266294000	-2.031440000	3.958081000
1	-0.410260000	-1.388059000	4.698680000
1	-4.221931000	-4.180534000	0.392617000
1	0.489785000	-2.372346000	-3.511782000
1	4.849759000	-2.262623000	1.182991000
6	5.224950000	-2.715288000	-1.852179000
6	5.525895000	-2.928612000	-3.194428000
1	6.547881000	-3.145137000	-3.490163000
6	4.518809000	-2.866876000	-4.179123000
1	4.781734000	-3.032737000	-5.219477000
6	3.194712000	-2.597276000	-3.840215000
1	2.430992000	-2.559482000	-4.610808000
1	5.999467000	-2.769024000	-1.093127000
6	-6.640582000	3.539207000	-1.918636000
6	-6.620093000	2.721256000	-0.768362000
1	-5.806207000	3.496055000	-2.611314000
6	-5.522727000	1.849508000	-0.519545000
6	-4.577770000	1.102821000	-0.311760000
6	-3.525551000	0.264646000	-0.081299000
6	-2.578245000	-0.483651000	0.124260000
6	-7.707324000	2.785115000	0.129581000
1	-7.696309000	2.159812000	1.016639000
6	-8.778927000	3.641086000	-0.119438000
1	-9.608230000	3.680052000	0.581210000
6	-8.789745000	4.446933000	-1.263001000

1	-9.626293000	5.112871000	-1.453443000
6	-7.717013000	4.391349000	-2.159394000
1	-7.718413000	5.015267000	-3.048667000
6	1.939061000	0.456211000	0.516697000
6	2.505121000	1.540357000	0.456266000
6	3.119055000	2.757463000	0.391047000
6	3.666488000	3.848724000	0.336145000
6	4.283545000	5.130084000	0.277140000
6	3.536060000	6.297010000	0.546388000
1	2.484528000	6.203237000	0.797920000
6	4.141396000	7.551167000	0.488930000
1	3.553562000	8.440260000	0.698624000
6	5.496938000	7.668119000	0.162841000
1	5.964952000	8.647187000	0.118523000
6	6.246505000	6.517985000	-0.106550000
1	7.299222000	6.602241000	-0.361177000
6	5.650210000	5.259191000	-0.050886000
1	6.229586000	4.365511000	-0.259896000

E = – 2035.2465935 a. u.
H = – 2034.646771 a. u.
G = – 2034.762134 a. u.

Por_1f – B_2OY_2-corrole, Y_1=Y_2= C≡C–C≡C–Ph (t_1)

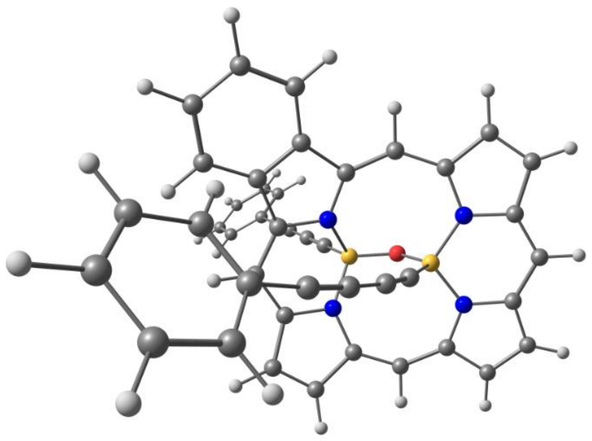

5	-0.748969000	0.715254000	-0.280382000
5	0.681803000	2.606977000	0.244743000
8	-0.604019000	2.005084000	0.188538000
6	0.088415000	0.702534000	-4.000523000
6	-0.083219000	1.473574000	-2.782521000
6	-0.005670000	2.881772000	-2.818612000
6	0.271511000	3.937865000	-1.924916000
6	0.306952000	5.331058000	-2.315442000
6	0.643204000	6.077695000	-1.213240000
6	0.826056000	5.157362000	-0.123088000
6	1.101652000	5.373742000	1.238288000
6	1.100587000	4.309425000	2.145429000
6	1.218478000	4.275306000	3.581447000
6	1.060608000	2.968741000	3.964927000
6	0.840298000	2.167871000	2.774758000
6	0.693338000	0.763182000	2.798810000
6	0.534209000	-0.292332000	1.880622000
6	0.942796000	-1.662926000	2.226786000
6	0.842278000	-2.447829000	1.054302000
6	0.357468000	-1.564691000	0.014934000
6	0.237250000	-1.773481000	-1.357526000
6	0.053060000	-0.693865000	-2.237624000
6	0.192295000	-0.621509000	-3.664805000
7	-0.162146000	0.565592000	-1.748072000
7	0.612515000	3.894599000	-0.600142000
7	0.894804000	3.025456000	1.714552000

7	0.135867000	-0.326896000	0.565939000
1	0.197605000	1.140237000	-4.984074000
1	0.085860000	5.692404000	-3.311023000
1	0.741431000	7.152565000	-1.146105000
1	1.383114000	5.136568000	4.214451000
1	1.078435000	2.573230000	4.971861000
1	0.388867000	-1.460388000	-4.318206000
1	-0.094859000	3.249699000	-3.838472000
1	1.265268000	6.383396000	1.596070000
1	0.857964000	0.384484000	3.804185000
1	0.425924000	-2.760243000	-1.763106000
6	1.701054000	-4.350243000	2.228662000
6	1.227177000	-3.792222000	1.043477000
6	1.806569000	-3.573115000	3.398971000
1	1.162566000	-4.384853000	0.136057000
1	2.183387000	-4.029962000	4.309084000
6	1.443333000	-2.227245000	3.406035000
1	1.552177000	-1.639996000	4.312463000
1	1.997954000	-5.394468000	2.252308000
6	-2.250400000	0.188983000	-0.263133000
6	-3.400157000	-0.232171000	-0.246690000
6	-4.685146000	-0.691204000	-0.224971000
6	-5.834609000	-1.105654000	-0.203472000
6	-7.174939000	-1.583797000	-0.172279000
6	-8.060857000	-1.170065000	0.845880000
1	-7.706629000	-0.480708000	1.605575000
6	-9.372421000	-1.640997000	0.873675000
1	-10.043020000	-1.314019000	1.663237000
6	-9.826116000	-2.529046000	-0.107561000
1	-10.848876000	-2.893569000	-0.082660000
6	-8.956203000	-2.944979000	-1.121268000
1	-9.302478000	-3.633954000	-1.886434000
6	-7.642750000	-2.479700000	-1.157545000
1	-6.966042000	-2.800416000	-1.943121000
6	1.951677000	1.737746000	-0.195460000
6	2.855447000	0.942062000	-0.419417000
6	3.739715000	-0.074867000	-0.621183000
6	4.472191000	-1.042203000	-0.767522000
6	5.246172000	-2.231199000	-0.871898000
6	4.900686000	-3.358348000	-0.093324000
6	5.644727000	-4.532547000	-0.189221000
1	5.369665000	-5.391976000	0.415794000
6	6.740806000	-4.607302000	-1.056263000
1	7.318708000	-5.524060000	-1.127515000
6	7.090206000	-3.495299000	-1.829931000
1	7.940785000	-3.547402000	-2.503494000
1	6.621811000	-1.451206000	-2.341380000
6	6.352562000	-2.315254000	-1.742340000

1	4.049355000	-3.297404000	0.577113000

E = – 2035.2635414 a. u.
H = – 2034.664131 a. u.
G = – 2034.778105 a. u.
